# Comprehensive Analysis of Competing Endogenous RNA Network Focusing on Long Noncoding RNA Involved in Cirrhotic Hepatocellular Carcinoma

**DOI:** 10.1155/2021/5510111

**Published:** 2021-06-22

**Authors:** Yuli Zhang, Dinggui Chen, Miaomiao Yang, Xianfeng Qian, Chunmei Long, Zhongwei Zheng

**Affiliations:** ^1^Department of Pharmacy, The Third People's Hospital of Changzhou, Changzhou City, Jiangsu Province 213001, China; ^2^Department of Gastroenterology, The Third People's Hospital of Changzhou, 300 Lanling North Road, Changzhou City, Jiangsu 213001, China

## Abstract

The role of long noncoding RNAs- (lncRNAs-) associated competing endogenous RNA (ceRNA) in the field of hepatocellular carcinoma (HCC) biology is well established, but the involvement of lncRNAs competing interactions in the progression of liver cirrhosis to HCC is still unclear. We aimed to explore the differential expression profiles of lncRNAs, microRNAs (miRNA), and messenger RNAs (mRNAs) to construct a functional ceRNA network in cirrhotic HCC. The lncRNA, miRNA, and mRNA expression datasets were obtained from Gene Expression Omnibus and The Cancer Genome Atlas. Based on miRanda and TargetScan, the HCC-specific ceRNA network was constructed to illustrate the coexpression regulatory relationship of lncRNAs, miRNAs, and mRNAs. The potential prognostic indicators in the network were confirmed by survival analysis and validated by qRT-PCR. A total of 74 lncRNAs, 36 intersection miRNAs, and 949 mRNAs were differentially expressed in cirrhotic HCC samples compared with cirrhosis samples. We constructed a ceRNA network, including 47 lncRNAs, 35 miRNAs, and 168 mRNAs. Survival analysis demonstrated that 2 lncRNAs (EGOT and SERHL), 4 miRNAs, and 40 mRNAs were significantly associated with the overall survival of HCC patients. Two novel regulatory pathways, EGOT-miR-32-5p-XYLT2 axis and SERHL-miR-1269a/miR-193b-3p-BCL2L1/SYK/ARNT/CHST3/LPCAT1 axis, were built up and contribute to the underlying mechanism of HCC pathogenesis. The higher-expressed SERHL was associated with a higher risk of all-cause death. The expressions of SERHL-miR-1269a-BCL2L1 were significantly different using qRT-PCR in vitro studies. lncRNAs EGOT and SERHL might serve as effective prognostic biomarkers and potential therapeutic targets in cirrhotic HCC treatment.

## 1. Introduction

Liver cancer is the sixth most common incident carcinoma and the fourth most common cause of malignant tumor mortality [1], being estimated to be 18.1 million new patients and 9.6 million cancer-related deaths throughout the world in 2018 [2]. Accounting for over 85% of primary liver cancers, hepatocellular carcinoma (HCC) is not evenly distributed among geographic regions, being accompanied by the increasing overall disease burden of HCC [3]. According to the etiology of cirrhosis, the risk factors for HCC mainly included hepatitis B virus infection, hepatitis C virus infection, alcohol, primary biliary cholangitis, autoimmune hepatitis, primary sclerosing cholangitis, and cryptogenic cirrhosis [4]. Therefore, liver cirrhosis plays a vital role in the pathogenesis of HCC. Based on the cirrhosis affecting progression to HCC, specific molecular biomarkers and potential regulatory mechanisms are essential and meaningful to the early diagnosis, treatment strategies, and the evaluation of prognosis.

Long noncoding RNAs (lncRNAs) are endogenous noncoding RNA molecules more than 200 nucleotides in length and can be subclassified into exonic, intronic, overlapping, and intergenic lncRNAs in nuclear or cytosolic fractions [5]. Given their regulation of cell proliferation, apoptosis, autophagy, epithelial-mesenchymal transition, and angiogenesis, cancer-specific lncRNAs are involved in the initiation, aggravation, and metastasis of HCC [6] and can serve as detectable diagnostic markers and effective therapeutic targets for HCC [7]. In the competing endogenous RNA (ceRNA) hypothesis, lncRNAs could act as competitive binding sites for the target microRNA (miRNA) response elements and then regulate the expression of corresponding messenger RNAs (mRNAs) [8]. The HCC-related deregulated ceRNA network was built to reveal the candidate prognostic cytoplasmic lncRNAs by integrated analysis in 371 HCC tissues and 50 healthy tissues from The Cancer Genome Atlas (TCGA) database [9]. However, the comprehensive analysis of the lncRNA-miRNA-mRNA ceRNA regulatory network in the progression of liver cirrhosis to HCC remains poorly characterized and be worth being further researched.

Given this context, we compared the differential expression of lncRNAs, miRNAs, and mRNAs between cirrhosis tissues and cirrhotic HCC tissues in the National Center for Biotechnology Information Gene Expression Omnibus (GEO) database and constructed the lncRNA-miRNA-mRNA ceRNA coexpression network. Finally, two lncRNAs were significantly correlated with the overall survival of cirrhotic HCC by the univariate Cox regression analysis, and their pair subnetworks were related to HCC-related signaling pathways. The previous correlational studies obtained the ceRNA expression in HCC samples and adjacent normal liver samples and explored the etiopathogenesis of normal liver to HCC. Hence, an integrated analysis of the ceRNA network to elaborate the biological processes and pathways on cancer-specific lncRNAs in cirrhotic HCC is still lacking. Our present study enrolled cirrhosis with and without HCC samples to focus on the pathologic process of cirrhosis to HCC, illuminating the underlying HCC pathogenesis of ceRNA coexpression network in patients with cirrhosis.

## 2. Materials and Methods

### 2.1. Microarray Datasets and Preprocessing

The lncRNAs, miRNAs, and mRNAs expression datasets were retrieved and downloaded from the GEO database by searching liver cirrhosis with and without HCC. GSE17967 (63 patients, 16 cirrhotic tissues with HCC, and 47 cirrhotic tissues without HCC, http://www.ncbi.nlm.nih.gov/geo/query/acc.cgi?acc=GSE17967) was included in our study and be used to analyze the differentially expressed lncRNAs and mRNAs [10]. Annotation pipeline of lncRNA transcripts was developed and obtained with Affymetrix Human Genome U133A 2.0 microarray. GSE21362 (73 patients, http://www.ncbi.nlm.nih.gov/geo/query/acc.cgi?acc=GSE21362) and GSE63046 (15 patients, http://www.ncbi.nlm.nih.gov/geo/query/acc.cgi?acc=GSE63046) were enrolled to profile and overlap the differentially expressed intersection miRNAs [11, 12]. The characteristics of each dataset were shown in Table S1. The expressions of specific lncRNAs, miRNAs, and mRNAs in cirrhotic HCC patients were downloaded from the National Institutes of Health TCGA Data Portal. TCGA-LIHC (377 patients, https://portal.gdc.cancer.gov/projects/TCGA-LIHC) was obtained for survival analysis. Differentially expressed lncRNAs, miRNAs, and mRNAs were extracted with restricted criteria, which were set at fold change (FC) > 1.2 and *P* < 0.05. Figure S1 depicts the flowchart for bioinformatics analysis, pointing out a cluster of lncRNA, miRNA, and mRNA.

### 2.2. Construction of the ceRNA Network

The regulatory network of lncRNAs, miRNAs, and mRNAs was constructed to demonstrate the regulatory relation. The ceRNA hypothesis is that lncRNAs, acting as the specific miRNA sponges, can bind to target miRNAs and then regulate corresponding mRNAs expression. We performed the lncRNAs-mRNAs network to construct the coexpression regulatory relation by estimating across the array (Fig. S2). The miRanda (http://www.microrna.org/) and TargetScan (http://www.targetscan.org/) were performed to predict the target miRNA, which play a negative role in regulating the expression of lncRNA and mRNA. Next, we overlapped the intersection datasets both in miRanda and TargetScan. Finally, we combined the lncRNA-miRNA and miRNA-mRNA pairs to build the ceRNA network, which was visualized by using Cytoscape v2.8.2 [13].

### 2.3. Survival Analysis

To investigate the prognostic significance of differentially expressed lncRNAs, miRNAs, and mRNAs, we performed the survival analysis by collecting the ceRNA expression and clinical information in 377 cirrhotic HCC samples from TCGA-LIHC database. We used the survival data to evaluate the association between overall survival in cirrhotic HCC and candidate corresponding RNA expression, which split into high-expression and low-expression groups using the median. Kaplan-Meier survival analysis with log-rank test was applied and compared to estimate the significant difference by a cutoff of *P* < 0.05. The statistically significant lncRNAs in the survival analysis were used to reconstruct a new ceRNA network by Cytoscape v2.8.2. Baseline characteristics for lncRNAs EGOT and SERHL were depicted in Table S2. To clarify the associations with overall survival and lncRNAs EGOT and SERHL, we analyzed the clinical characteristics of enrolled patients, including age, sex, race, pathologic stage, Child-Pugh score, neoplasm histologic grade, Ishak fibrosis score, vascular invasion, residual tumor grade, radiation therapy, neoadjuvant therapy, embolization therapy, family history of cancer, risk factors, and biomarkers (AFP and albumin) (Table S3).

### 2.4. Cell Culture and Quantitative Real-Time Polymerase Chain Reaction (qRT-PCR)

We obtained the Huh7 human hepatoma cell line and LX-2 human hepatic fibrosis cell line from the Shanghai Institute of Biochemistry and Cell Biology (Shanghai, China). Huh7 cells were cultured with the Dulbecco's Modified Eagle's Medium (Thermo Fisher Scientific, Waltham, MA) containing 10% fetal bovine serum (FBS), penicillin (100 U/mL), L-glutamine (2 mM), and streptomycin (100 *μ*g/mL) at 37°C in a humidified incubator (5% CO_2_). LX-2 cells were cultured in Medium 199 (Sigma-Aldrich, St. Louis, Missouri, USA) containing 10% FBS in a 5% CO2 atmosphere at 37°C. All the cell lines were seeded in microplates and maintained for 14 days. Total RNA was collected by a miRNeasy Mini Kit (Qiagen, Hilden, Germany) under the manufacturer's instructions. Then, the quantity, integrity, and purity were assessed using a NanoDrop 2000 (Thermo Fisher Scientific, MA, USA). 50 ng of RNA was reverse-transcribed to cDNA by an iScript cDNA Synthesis Kit (170-8891, Bio-RAD). Quantitative PCR was performed using the 7900HT Rapid Real-Time Biosystems using SYBR-GREEN (170-8882AP, BIO-RAD) technology. Primers are listed in Table S4.

### 2.5. Statistical Analyses

Categorical variables were expressed as frequencies and proportions and compared using chi-square, Pearson's chi-squared, or Fisher exact test. Continuous variables were expressed as means with standard deviation (SD) or medians with interquartile ranges (IQRs) and compared using Student's *t*-tests or Wilcoxon rank-sum test. The absolute values of fold change > 1.2 and *P* < 0.05 were considered to indicate significant difference. The univariate and multivariate Cox proportional hazard regression models were conducted for discovering the connections between ceRNAs and overall survival. Backward stepwise selection was applied by using Akaike's information criterion to identify variables for multivariable Cox proportional hazard regression analysis. Then, we constructed a nomogram based on the multivariable Cox regression analyses to visualize the probability of 5-year survival in HCC patients. Variance inflation factor (VIF) assessed the multicollinearity of independent variables in the Cox regression model (VIF ≥ 10 suggests multicollinearity). Hazard ratios (HRs) were presented with their 95% confidence intervals (CIs). All analyses were implemented using the SPSS software (version 21.0, IBM, Chicago, Illinois, USA) and the R software (version 3.6.2, R Development Core Team, Vienna, Austria).

## 3. Results

### 3.1. Differential Expression of lncRNAs, miRNAs, and mRNAs in Cirrhotic HCC

**Figure 1:**
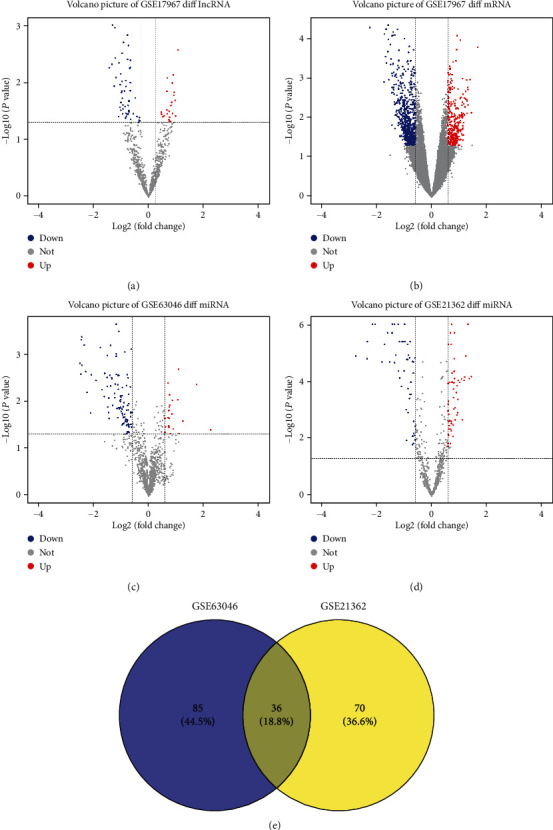
Differential analysis of lncRNAs, miRNAs, and mRNAs in cirrhotic patients with HCC compared with those in cirrhotic patients without HCC (fold change > 1.2; *P* < 0.05). (a, b) Volcano picture showing the upregulated (in red), downregulated (in blue), and undifferentiated (in gray) lncRNAs and mRNAs in their expression of GSE17967. (c, d) Volcano picture showing the upregulated (in red), downregulated (in blue), and undifferentiated (in gray) miRNAs in their expression of GSE63046 and GSE21362. (e) Venn diagram analysis of differentially expressed miRNA obtained from GSE63046 and GSE21362. The number in the overlapping portion indicates the common miRNAs. lncRNA: long noncoding RNA; miRNA: microRNA; mRNA: messenger RNA; HCC: hepatocellular carcinoma.

With the tumorigenesis of HCC in cirrhotic patients, a total of 74 lncRNAs from GSE17967, 36 intersection miRNAs from GSE21362 and GSE63046, and 949 mRNAs from GSE17967 were identified with the restricted criteria of FC > 1.2 and *P* < 0.05 by bioinformatics analysis (Figure 1). The 74 HCC-specific differentially expressed lncRNAs were composed of 22 upregulated and 52 downregulated lncRNAs in GSE17967 (Figure 1(a); Table 1; Table S5). For 949 differentially expressed mRNAs in the GSE17967 dataset, there were 291 upregulated and 658 downregulated HCC-specific mRNAs (Figure 1(b); Table S6). We initially identified 121 miRNAs from GSE63046 (Figure 1(c)) and 106 miRNAs from the GSE21362 data set (Figure 1(d)), respectively. The intersections of the two datasets of differentially expressed miRNAs consisted of 36 (18.8%) miRNAs (5 upregulated and 31 downregulated, Figure 1(e); Table S7). These data provided a new clue to reveal the essential genes involved in the sequential progression from liver cirrhosis to HCC.

### 3.2. Construction of ceRNA Network

**Figure 2:**
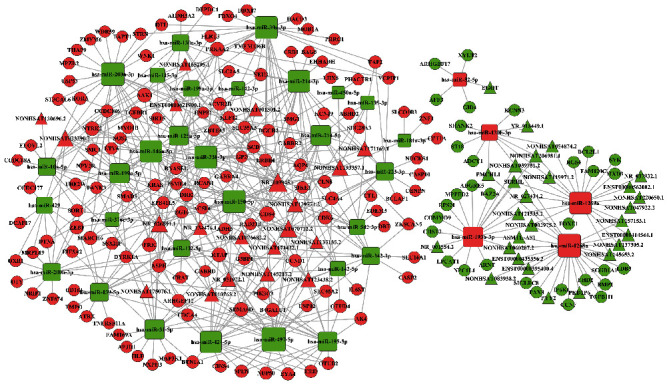
The ceRNA network of lncRNA-miRNA-mRNA. Triangles represent lncRNAs; rectangles with rounded corners represent miRNA; balls represent mRNAs. Red represents upregulated RNAs, and green represents downregulated RNAs. lncRNA: long noncoding RNA; miRNA: microRNA; mRNA: messenger RNA.

The lncRNA-mRNA coexpression network was built by differentially expressed lncRNAs and mRNAs to investigate the critical regulatory relations. A total of 16 lncRNAs (6 upregulated lncRNAs; 10 downregulated lncRNAs) and 219 mRNAs (96 upregulated mRNAs; 123 downregulated mRNAs) were enrolled in this network for further analysis. Next, based on miRanda and TargetScan analysis, the intersection differentially expressed miRNAs were used to predict the miRNAs negative targeted mRNAs, identifying 35 miRNAs negatively correlated to 360 mRNAs. Subsequently, we confirmed 36 miRNAs and the corresponding negative regulation to 69 lncRNAs. The lncRNA-miRNA-mRNA ceRNA coexpression network was built up based on the targeted interactions. We identified that 47 lncRNAs had regulatory effects on 35 intersection miRNAs (Table 2). Next, 35 miRNAs were interacting with 168 specific mRNAs (Table 3). Finally, the numbers of lncRNAs, miRNAs, and mRNAs were decreased to 47, 35, and 168, respectively (Figure 2).

### 3.3. Prognosis Prediction for Differentially Expressed lncRNAs, miRNAs, and mRNAs

**Figure 3:**
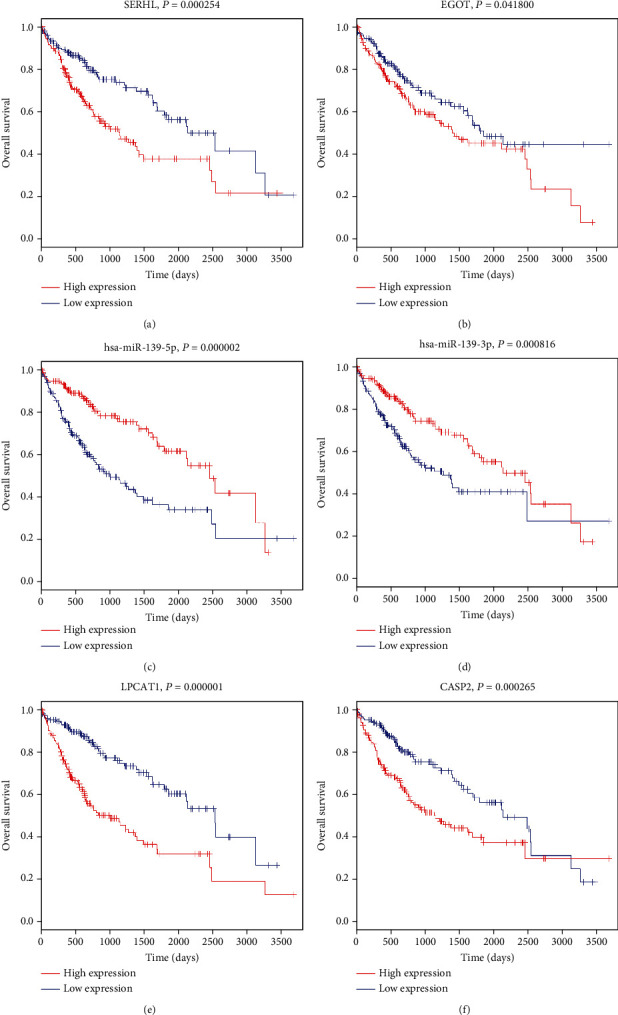
Prognosis prediction for differentially expressed ceRNAs. Kaplan-Meier survival curves for top two lncRNAs (a, b), miRNAs (c, d), and mRNAs (e, f) associated with overall survival. Horizontal axis, overall survival time, days; vertical axis, survival function. Red represents high expression samples, and blue represents low expression samples. ceRNA: competing endogenous RNA; lncRNA: long noncoding RNA; miRNA: microRNA; mRNA: messenger RNA.

To identify the HCC-specific ceRNAs with prognostic characteristics, we enrolled 377 cirrhotic HCC patients from the TCGA-LIHC database, and the univariate Cox regression model was implemented based on the expression of these lncRNAs, miRNAs, and mRNAs, which illustrated the relationship between key ceRNAs and overall survival of HCC patients. Our results demonstrated that 2 lncRNAs, 4 miRNAs, and 40 mRNAs were significantly correlated to the overall survival in patients with cirrhotic HCC (Fig. S3). The expressions of top two lncRNAs (EGOT and SERHL), top two miRNAs (hsa-miR-139-5p and hsa-miR-139-3p), and top two mRNAs (LPCAT1 and CASP2) were depicted in Figure 3 (*P* < 0.05). And Kaplan-Meier survival analyses revealed that these two lncRNAs, EGOT and SERHL, had negative correlation with overall survival in the progression of HCC. According to pathway analysis, we further demonstrated the expression pattern of EGOT and SERHL lncRNAs as well as their pair subnetworks (EGOT-miR-32-5p-XYLT2 axis and SERHL-miR-1269a/miR-193b-3p-BCL2L1/SYK/ARNT/CHST3/LPCAT1 axis) (Fig. S4).

### 3.4. The Associations between Key lncRNAs and Clinical Characteristics

Multivariate Cox analysis was implemented to investigate the predictors of a clinical prognosis of cirrhotic HCC (Table S8). Due to less than 10 points of all VIF in the multivariate Cox model, there was no evidence of multicollinearity in independent variables. Compared with stage I, a later pathologic stage of stages III and IV was associated with a lower overall survival rate (HR 1.59, 95% CI 1.05-2.43, and *P* = 0.029 and HR 4.80, 95% CI 1.49-15.45, and *P* = 0.009, respectively), while there was no significant different in the pathologic stage of stage II (HR 0.93, 95% CI 0.58-1.50, *P* = 0.766). In patients with cirrhotic HCC, a higher alpha-fetoprotein (AFP) (HR 1.73, 95% CI 1.18-2.55, *P* = 0.005) and a higher albumin (HR 2.45, 95% CI 1.61-3.72, *P* < 0.001) were related to a lower overall survival rate. In Cox hazard modeling for survival free from total mortality, the unadjusted HR for high-expressed EGOT group was 1.48 (95% CI 1.05-2.09, *P* = 0.027), which decreased to 1.35 (95% CI 0.95-1.92, *P* = 0.091) in a multivariable model that included pathologic stage, AFP, and albumin (Table S3). The unadjusted HR for high-expressed SERHL group was 1.97 (95% CI 1.39-2.81, *P* < 0.001), which decreased to 1.74 (95% CI 1.22-2.49, *P* = 0.002) in the adjusted multivariable model. A nomogram was then constructed by using AFP, serum albumin, pathologic stage, and lncRNA EGOT (Fig. S5A) or SERHL (Fig. S5B) to visualize the prediction model of 5-year survival probability.

### 3.5. qRT-PCR Verification

**Figure 4:**
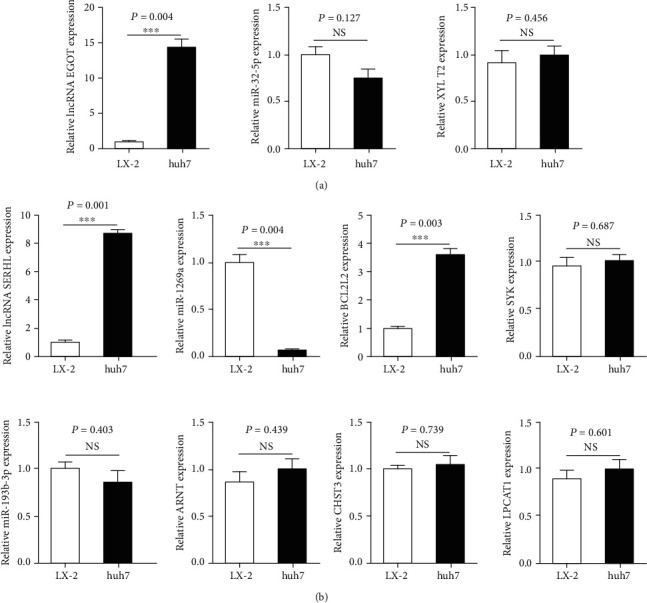
qRT-PCR validation of two key lncRNAs and their pair subnetworks in vitro studies. (a) Comparison of the expression levels of lncRNA EGOT-miR-32-5p-XYLT2 axis between Huh7 cells and LX-2 cells. (b) Comparison of the expression levels of lncRNA SERHL-miR-1269a/miR-193b-3p-BCL2L1/SYK/ARNT/CHST3/LPCAT1 axis between Huh7 cells and LX-2 cells. qRT-PCR: quantitative real-time polymerase chain reaction; lncRNA: long noncoding RNA.

lncRNA EGOT-miR-32-5p-XYLT2 axis and lncRNA SERHL-miR-1269a/miR-193b-3p-BCL2L1/SYK/ARNT/CHST3/LPCAT1 axis were selected to establish the validity and reliability of our results. The expressions of two key lncRNAs and their pair subnetworks were evaluated in Huh7 cells compared to LX-2 cells. As shown in Figure 4(a), lncRNA EGOT was significantly higher in Huh7 cells than in LX-2 cells (*P* = 0.004), suggesting that lncRNA EGOT was upregulated in human hepatoma cells compared with human hepatic fibrosis cells in vitro studies. However, the expressions of miR-32-5p and XYLT2 were not significantly different in Huh7 cells and LX-2 cells (*P* = 0.127 and *P* = 0.456, respectively). Similarly, lncRNA SERHL and BCL2L1 were significantly higher expressed (*P* = 0.001 and *P* = 0.003, respectively), and miR-1269a was significantly lower expressed (*P* = 0.004) in Huh7 cells than in LX-2 cells (Figure 4(b)). However, no significant differences were found in the other subnetwork's expression, including miR-193b-3p, ARNT, CHST3, LPCAT1, and SYK (Figure 4(b)).

## 4. Discussion and Conclusions

Our study demonstrated the differential expression profiling of lncRNAs, miRNAs, and mRNAs in cirrhotic HCC. Next, based on miRanda and TargetScan, we explored the coexpression regulatory relationships between lncRNA, miRNA, and mRNA. Then, we built a functional ceRNA network to provide a global view of HCC-associated differentially expressed RNAs with interregulated 47 lncRNAs, 35 miRNAs, and 168 mRNAs. We also uncovered that two differentially expressed lncRNAs (EGOT and SERHL), and their pair subnetworks could affect the prognosis of cirrhotic HCC and predict the overall survival of patients with HCC. This finding provides a novel insight into the initiation and development of cirrhotic HCC.

Emerging evidences indicate that the aberrant expression of lncRNAs, developing into a highly active research hotspot, plays significant roles in tumorigenesis and acts as novel molecular biomarkers for the diagnosis and prognosis of HCC. More recently, a growing body of research shows that the construction of the ceRNA network reveals its diagnostic and prognostic value in HCC by comprehensive analysis [14–17]. For example, lncRNA SSTR5-AS1, acting as a ceRNA, sponge mir-15b-5p to regulate carbonic anhydrase 2 (CA2) function in the progression of hepatitis B virus-related hepatocellular carcinoma [18].

In the present study, we highlighted lncRNAs EGOT and SERHL might be two critical lncRNAs that act as diagnostic and prognostic biomarkers in the progression of liver cirrhosis to HCC. Previous work has demonstrated that lncRNA EGOT is involved in the progress of breast cancer [19], gastric cancer [20], head and neck squamous cell carcinomas [21], renal cell carcinoma [22], and hepatitis C virus-induced liver tumors [23]. Moreover, higher expression of lncRNA SERHL is significantly correlated with patient's shorter overall survival in HCC, not involving in further research on the functional implications of SERHL [24]. Our results suggest that lncRNA EGOT-miR-32-5p-XYLT2 axis and lncRNA SERHL-miR-1269a/miR-193b-3p-BCL2L1/SYK/ARNT/CHST3/LPCAT1 axis could contribute to the regulatory mechanism underlying the pathogenetic process of HCC. lncRNA EGOT synergistically increased the expression of hsa-miR-32-5p and then decreased the expression of XYLT2, relating to significant regulation of key HCC-related signaling pathways such as glycosaminoglycan biosynthesis and metabolic pathways (Fig. S4). lncRNA SERHL synergistically upregulated the expression of hsa-miR-1269a and hsa-miR-193b-3p and then downregulated the expression of BCL2L1, SYK, ARNT, CHST3, and LPCAT1, which are associated with the significant regulation of key HCC-related signaling pathways such as Ras signaling pathway, PI3K-Akt signaling pathway, NF-kappa B signaling pathway, and JAK-STAT signaling pathway (Fig. S4). Consistent with previous research, lncRNAs EGOT and SERHL are negatively associated with the overall survival of patients with cirrhotic HCC. These two lncRNAs may be exploited as potential effective biomarkers in the diagnosis and prognosis of HCC.

In conclusion, we identified the differential expression of cancer-specific lncRNAs, miRNAs, and mRNAs in the progression of liver cirrhosis to HCC and constructed the ceRNA network to reveal their genetic interactions. The higher-expressed SERHL was associated with a higher risk of all-cause death. The expression of SERHL-miR-1269a-BCL2L1 was significantly related to cirrhotic HCC in vitro studies. Importantly, as candidate biomarkers for HCC prognosis prediction, two potential unexplored lncRNAs EGOT and SERHL showed significantly negative associations with overall survival, and their pair subnetworks could provide a novel clue to future study into the underlying regulatory mechanism of cirrhotic HCC.

## Figures and Tables

**Table 1:** Differentially expressed lncRNAs between cirrhosis and cirrhotic HCC samples.

lncRNAs	Regulation	Fold change	*P* value
BTF3P11	Down	-2.663	0.006
NONHSAT060287.2	Down	-2.494	0.005
XR_250850.2	Down	-2.462	0.001
NR_027259.1	Down	-2.361	0.012
NR_001284.2	Down	-2.320	0.001
EGOT	Down	-2.299	0.004
NONHSAT039751.2	Down	-2.256	0.008
NONHSAT126168.2	Down	-2.113	0.040
NR_001554.2	Down	-2.095	0.005
NONHSAT004151.2	Down	-2.032	0.027
PMCHL1	Down	-2.027	0.011
NR_001559.2	Down	-2.010	0.023
ENST00000414544.1	Down	-2.005	0.007
SERHL	Down	-2.002	0.031
ASMTL-AS1	Down	-1.991	0.023
MAPKAPK5-AS1	Down	-1.974	0.015
NONHSAT047922.2	Down	-1.947	0.014
TMEM92-AS1	Down	-1.937	0.010
NONHSAT121535.2	Down	-1.929	0.003
NONHSAT145653.2	Down	-1.925	0.019
NR_027434.2	Down	-1.910	0.035
NONHSAT220650.1	Down	-1.901	0.025
ENST00000562082.1	Down	-1.898	0.029
XR_934561.1	Down	-1.871	0.002
ENST00000420452.5	Down	-1.827	0.014
PYY2	Down	-1.805	0.027
NONHSAT157153.1	Down	-1.775	0.042
NONHSAT041143.2	Down	-1.762	0.009
NONHSAT220262.1	Down	-1.759	0.035
ENST00000395400.4	Down	-1.757	0.012
MLLT10P1	Down	-1.745	0.039
ENST00000435356.2	Down	-1.707	0.036
PKD1P6	Down	-1.691	0.001
NONHSAT054874.2	Down	-1.687	0.004
NONHSAT206981.1	Down	-1.680	0.036
NONHSAT137305.2	Down	-1.666	0.025
NACAP1	Down	-1.655	0.002
LOC105376196	Down	-1.637	0.031
ENST00000497872.3	Down	-1.636	0.033
NONHSAT094826.2	Down	-1.632	0.022
NONHSAT192084.1	Down	-1.629	0.021
NONHSAT185078.1	Down	-1.617	0.023
TNXA	Down	-1.587	0.005
NONHSAT002975.2	Down	-1.581	0.014
NONHSAT119971.2	Down	-1.561	0.044
NONHSAT212252.1	Down	-1.465	0.035
NR_002174.2	Down	-1.395	0.019
XR_918449.1	Down	-1.337	0.039
NONHSAT083938.2	Down	-1.278	0.050
NR_135506.1	Down	-1.268	0.046
NR_037932.1	Down	-1.258	0.041
NONHSAT047228.2	Down	-1.240	0.047
NONHSAT135135.2	Up	2.094	0.003
NONHSAT070712.2	Up	1.960	0.039
NONHSAT129571.2	Up	1.944	0.021
NONHSAT145215.2	Up	1.894	0.015
NONHSAT123438.2	Up	1.880	0.017
NONHSAT001301.2	Up	1.849	0.007
NONHSAT171169.1	Up	1.824	0.035
NONHSAT175076.1	Up	1.809	0.010
NONHSAT076682.2	Up	1.782	0.022
LOC105370914	Up	1.723	0.048
NONHSAT193357.1	Up	1.711	0.029
ENST00000621900.1	Up	1.693	0.041
NONHSAT010763.2	Up	1.677	0.048
NR_134476.1	Up	1.668	0.045
NONHSAT171432.1	Up	1.601	0.008
NR_026891.1	Up	1.598	0.035
NONHSAT023390.2	Up	1.579	0.014
NONHSAT165295.1	Up	1.575	0.031
NR_051972.1	Up	1.477	0.038
COX7A2P2	Up	1.430	0.040
NONHSAT130696.2	Up	1.384	0.036
NR_003945.1	Up	1.373	0.033

lncRNA: long noncoding RNA; HCC: hepatocellular carcinoma.

**Table 2:** miRNAs targeting specific key lncRNAs in cirrhotic HCC.

Key lncRNAs	miRNAs
ASMTL-AS1	hsa-miR-1268a, hsa-miR-193b-3p
EGOT	hsa-miR-32-5p
PKD1P6	hsa-miR-1268a
PMCHL1	hsa-miR-1268a, hsa-miR-130b-3p
PYY2	hsa-miR-1268a
SERHL	hsa-miR-1269a, hsa-miR-193b-3p
XR_918449.1	hsa-miR-130b-3p
NR_134476.1	hsa-miR-139-5p, hsa-miR-150-5p, hsa-miR-199a-3p, hsa-miR-200a-3p, hsa-miR-214-5p, hsa-miR-497-5p
NR_051972.1	hsa-miR-125a-5p, hsa-miR-150-5p, hsa-miR-195-5p, hsa-miR-199a-5p, hsa-miR-214-3p, hsa-miR-424-5p, hsa-miR-497-5p
NR_037932.1	hsa-miR-1269a
NR_027434.2	hsa-miR-1268a, hsa-miR-1269a, hsa-miR-193b-3p
NR_026891.1	hsa-miR-10a-5p, hsa-miR-125a-5p, hsa-miR-139-5p, hsa-miR-145-3p, hsa-miR-150-5p, hsa-miR-200a-3p, hsa-miR-200b-3p, hsa-miR-214-5p, hsa-miR-31-5p, hsa-miR-429, hsa-miR-542-3p
NR_003945.1	hsa-miR-125a-5p, hsa-miR-130a-3p, hsa-miR-139-5p, hsa-miR-142-3p, hsa-miR-146a-5p, hsa-miR-150-5p, hsa-miR-199a-3p, hsa-miR-214-3p, hsa-miR-214-5p, hsa-miR-223-3p, hsa-miR-30a-3p, hsa-miR-31-5p, hsa-miR-338-3p, hsa-miR-542-3p
NR_001554.2	hsa-miR-193b-3p
NONHSAT220650.1	hsa-miR-1269a
NONHSAT206981.1	hsa-miR-1269a, hsa-miR-130b-3p, hsa-miR-193b-3p
NONHSAT193357.1	hsa-miR-125a-5p, hsa-miR-132-5p, hsa-miR-139-3p, hsa-miR-146a-5p, hsa-miR-150-5p, hsa-miR-181a-3p, hsa-miR-214-3p, hsa-miR-214-5p, hsa-miR-342-3p, hsa-miR-450a-5p
NONHSAT175076.1	hsa-miR-132-5p, hsa-miR-31-5p, hsa-miR-376c-3p, hsa-miR-424-5p
NONHSAT171432.1	hsa-miR-199a-5p, hsa-miR-214-5p, hsa-miR-31-5p, hsa-miR-342-3p
NONHSAT171169.1	hsa-miR-139-3p, hsa-miR-150-5p, hsa-miR-214-3p, hsa-miR-214-5p, hsa-miR-223-3p, hsa-miR-338-3p, hsa-miR-542-3p
NONHSAT165295.1	hsa-miR-145-3p, hsa-miR-199a-3p
NONHSAT157153.1	hsa-miR-1268a, hsa-miR-1269a
NONHSAT145653.2	hsa-miR-1268a, hsa-miR-1269a
NONHSAT145215.2	hsa-miR-132-5p, hsa-miR-142-5p, hsa-miR-146a-5p, hsa-miR-30a-3p, hsa-miR-338-3p, hsa-miR-424-5p, hsa-miR-497-5p
NONHSAT137305.2	hsa-miR-1268a, hsa-miR-1269a
NONHSAT135135.2	hsa-miR-150-5p, hsa-miR-214-3p, hsa-miR-497-5p
NONHSAT130696.2	hsa-miR-10a-5p
NONHSAT129571.2	hsa-miR-10a-5p, hsa-miR-145-3p, hsa-miR-146a-5p, hsa-miR-199a-5p, hsa-miR-30a-3p, hsa-miR-342-3p, hsa-miR-497-5p, hsa-miR-542-3p
NONHSAT123438.2	hsa-miR-150-5p, hsa-miR-195-5p, hsa-miR-199a-3p, hsa-miR-214-3p, hsa-miR-338-3p, hsa-miR-342-3p, hsa-miR-497-5p
NONHSAT121535.2	hsa-miR-1269a, hsa-miR-193b-3p
NONHSAT119971.2	hsa-miR-1269a, hsa-miR-130b-3p
NONHSAT083938.2	hsa-miR-1268a
NONHSAT076682.2	hsa-miR-132-5p, hsa-miR-200b-3p, hsa-miR-214-5p, hsa-miR-223-3p
NONHSAT070712.2	hsa-miR-10a-5p, hsa-miR-130a-3p, hsa-miR-139-3p, hsa-miR-142-5p, hsa-miR-150-5p, hsa-miR-195-5p, hsa-miR-214-5p, hsa-miR-223-3p, hsa-miR-30a-3p, hsa-miR-338-3p, hsa-miR-424-5p, hsa-miR-497-5p
NONHSAT060287.2	hsa-miR-1268a, hsa-miR-193b-3p
NONHSAT054874.2	hsa-miR-1269a, hsa-miR-130b-3p
NONHSAT047922.2	hsa-miR-1269a
NONHSAT039751.2	hsa-miR-1268a, hsa-miR-130b-3p, hsa-miR-193b-3p
NONHSAT023390.2	hsa-miR-125a-5p, hsa-miR-130a-3p, hsa-miR-132-5p, hsa-miR-145-3p, hsa-miR-146a-5p, hsa-miR-150-5p, hsa-miR-199a-5p, hsa-miR-200a-3p, hsa-miR-200b-3p, hsa-miR-429
NONHSAT010763.2	hsa-miR-125a-5p, hsa-miR-195-5p, hsa-miR-199a-5p, hsa-miR-200a-3p, hsa-miR-424-5p, hsa-miR-497-5p
NONHSAT002975.2	hsa-miR-1268a, hsa-miR-1269a, hsa-miR-193b-3p
NONHSAT001301.2	hsa-miR-125a-5p, hsa-miR-150-5p, hsa-miR-214-3p, hsa-miR-338-3p
ENST00000621900.1	hsa-miR-145-3p, hsa-miR-338-3p
ENST00000562082.1	hsa-miR-1269a
ENST00000435356.2	hsa-miR-1268a
ENST00000414544.1	hsa-miR-1268a, hsa-miR-1269a
ENST00000395400.4	hsa-miR-1268a

miRNA: microRNA; lncRNA: long noncoding RNA; HCC: hepatocellular carcinoma.

**Table 3:** miRNAs targeting specific mRNAs in cirrhotic HCC.

miRNAs	mRNAs
hsa-miR-497-5p	ARHGEF12, ASPH, B4GALT1, BTN1A1, CCND1, CD84, CDCA4, EED, EYA4, GINS4, MTAP, NUP50, OTUB2, OTUD4, SEMA6D, SLC45A2, SPEN, USP12
hsa-miR-1268a	BMP1, CCNF, EHD2, IP6K1, LDB3, PAX8, SCGB1A1, SIGLEC8, TGFB1I1
hsa-miR-200a-3p	AAK1, AQP4,DTL, FRK, G3BP1, KLF12, MARCH6, MPZL2, PANK3, RORA, SLC35A3, ST3GAL6, STRN, TAPT1, THAP9, USP53, WDR59, WNK1, ZEB1, ZMYM6
hsa-miR-150-5p	ACSL6, ADH6, B4GALT1, CD84, DIO2, MARCH6, NTRK2, PRKAA2, RAD23B, RCAN1, SLC4A4, ZEB1
hsa-miR-338-3p	AAK1, ACVR2B, CLN8, DGCR2, DTNA, FRK, GABRA4, GP2, MARCH6, NTRK2, PRKAA2, RNASEL, SEMA6D, SSX2IP, ZBTB43, ZG16
hsa-miR-30a-3p	AQP4, ASPH, CCDC186, CDK6, DDX17, DTL, ERBB4, FBXO4, FLRT3, HACD3, MOB1A, MTAP, NTRK2, PRKAA2, PRRC1, STRN, TAP2, TMEM106B, VCPIP1
hsa-miR-146a-5p	AAK1, CD84, DTNA, ERBB4, ETV6, FRK, KLF12, MTAP, MYO1B, NTRK2, PRKAA2, RNASEL, SCD, SIKE1, SORT1, SSX2IP, UBE2W
hsa-miR-1269a	BCL2L1, FAM120C, FOXE1, MXD3, RGS4, SYK
hsa-miR-214-3p	ACVR2B, BAG5, CD84, CLN8, CRB1, DGCR2, EHHADH, FRK, KCNJ9, KRAS, LHX6, NEU3, SEMA6D, SLC2A5, TMEM106B
hsa-miR-424-5p	ASPH, B4GALT1, BTN1A1, CD84, CDCA4, EED, EYA4, GINS4, MTAP, NUP50, OTUB2, OTUD4, SEMA6D, SLC45A2, SLC4A4, SPEN
hsa-miR-214-5p	ABHD2, CCND1, CD84, DGCR2, GABBR2, KLF12, SCD, SLC28A3, ZKSCAN5
hsa-miR-193b-3p	ADGRE5, ARNT, BAZ2A, CHST3, COMMD9, LPCAT1, MPPED2, NPC1L1, RPS21
hsa-miR-31-5p	AK4, AP3D1, BTN1A1, DYRK1A, EIF5A2, EPB41L5, FAM169A, FRK, G3BP1, HLF, MAP3K1, NXPH3, SEMA6D
hsa-miR-199a-5p	ACVR2B, ARHGEF12, DYRK1A, ETV6, FLRT3, MARCH6, NEU3, NPY2R, SLC35A3, SMC4, SOS2, ST3GAL6
hsa-miR-195-5p	AK4, ARHGEF12, B4GALT1, EED, EYA4, MTAP, NUP50, OTUB2, OTUD4, SEMA6D, SLC4A4, SPEN
hsa-miR-125a-5p	AAK1, ASPH, ENPP1, FRK, PRKAA2, RORA, SIRT5, SLC2A5, SMG1
hsa-miR-139-5p	ASPH, ATRX, CD164, CDCA4, MARCH6, PSME4, SSX2IP, TMPO, TNFRSF11A, ZEB1
hsa-miR-223-3p	BCLAF1, CASP10, CENPN, KCNJ9, NUCKS1, PIK3C3, SLC4A4, TAP2, VCPIP1
hsa-miR-200b-3p	DTNA, MRPL19, MTAP, NRIP1, OXR1, PIK3C3, SORT1, UTY, ZEB1, ZNF674
hsa-miR-10a-5p	AAK1, ARHGEF12, CCDC177, CCDC186, CCDC88A, ELOVL2, RORA, SORT1, TGFBR1
hsa-miR-342-3p	AK4, AQP4, CASP2, CDK6, CLN8, G3BP1, SLC16A1, SLC45A2, ZKSCAN5
hsa-miR-130a-3p	AAK1, ALDH3A2, DEPDC1, EPB41L5, NEU3, PRKAA2, VCPIP1, WNK1
hsa-miR-429	CCDC177, DCAF17, DTNA, EIF5A2, MRPL19, NTRK2, OXR1, UBE2W, ZEB1
hsa-miR-132-5p	CCND1, CRAT, DIO2, GABRD, SCD, ZG16
hsa-miR-199a-3p	ACVR2B, DIO2, EPB41L5, ERBB4, NTRK2, TMEM106B
hsa-miR-130b-3p	ADCY1, KCNN3, SHANK2, ST18
hsa-miR-542-3p	B4GALT1, CDK6, DBT, EDEM3, UFL1
hsa-miR-142-5p	CDK6, ERBB4, IL6ST, OTUD4, PIK3C3, SIKE1
hsa-miR-145-3p	CD84
hsa-miR-376c-3p	CCDC186, DIO2, KRAS, PANK3, SMAD5
hsa-miR-142-3p	CD84, PSME4, SMG1, TGFBR1
hsa-miR-139-3p	ABHD2, TAP2
hsa-miR-32-5p	AFF3, ARHGEF17, GID4, XYLT2
hsa-miR-181a-3p	CPT1A, SLCO1B3, ZNF3
hsa-miR-450a-5p	PHACTR1, SMG1

miRNA: microRNA; mRNA: messenger RNA; HCC: hepatocellular carcinoma.

## Data Availability

The datasets of lncRNAs, miRNAs, and mRNAs have been contributed by GSE17967, GSE21362, GSE63046, and TCGA-LIHC. The data of the current study are available from the corresponding author on reasonable request.

## Abbreviations

HCC: Hepatocellular carcinoma
lncRNAs: Long noncoding RNAs
ceRNA: Competing endogenous RNA
miRNA: MicroRNA
mRNA: Messenger RNA
TCGA: The Cancer Genome Atlas
GEO: Gene Expression Omnibus
FC: Fold change
FBS: Fetal bovine serum
SD: Standard deviation
IQR: Interquartile range
VIF: Variance inflation factor
HR: Hazard ratios
CI: Confidence interval.

## Supplementary Materials

Supplementary Materials The file of Supplementary Materials.
