# Supplementary material for: Comprehensive Analysis of Competing Endogenous RNA Network Focusing on Long Noncoding RNA Involved in Cirrhotic Hepatocellular Carcinoma
Source: Anal Cell Pathol (Amst). 2021 Jun 22;2021:5510111. doi: 10.1155/2021/5510111 (PMC8245234; doi:10.1155/2021/5510111)
Supplement: Supplementary Materials — The file of Supplementary Materials. [file 5510111.f1.docx]

**SUPPLEMENTAL MATERIAL**

Including 5 supplemental figures and 8 supplemental tables.

**Fig. S1 The flowchart of ceRNA network construction and bioinformatics analysis.** ceRNA, competing endogenous RNA; lncRNA, long noncoding RNA; miRNA, microRNA; mRNA, messenger RNA.

**Fig. S2 LncRNAs-mRNAs network.** Triangles represent lncRNAs; balls represent mRNAs. Red represents upregulated RNAs, and green represents downregulated RNAs. lncRNA, long noncoding RNA.

**Fig. S3 All diﬀerentially expressed lncRNAs (A), miRNAs (B) and mRNAs (C) associated with overall survival from TCGA-LIHC.** Horizontal axis, overall survival time, days; vertical axis, survival function. Red represents high expression samples and blue represents low expression samples. lncRNA, long noncoding RNA; miRNA, microRNA; mRNA, messenger RNA.

**Fig. S4 Pathway analysis of regulatory networks constructed for SERHL and EGOT target lncRNAs.** Triangles represent lncRNAs; rectangles with rounded corners represent miRNA; balls represent mRNAs; arrows represent pathways. Red represents upregulated RNAs, green represents downregulated RNAs and yellow represents different pathways. lncRNA, long noncoding RNA; miRNA, microRNA; mRNA, messenger RNA.

**Fig. S5** **Nomogram to predict the probability of 5-year survival in HCC patients based on AFP, serum albumin, pathologic stage, and lncRNA.** The prediction nomograms consisted of lncRNA EGOT (A) and SERHL (B). Points are assigned for each variable by drawing a line upward from the corresponding variable to the points line. The sum of the points plotted on the total points line corresponds with the prediction of 5-year survival probability.

**Table S1** (GSE21362); (GSE17967); (GSE63046)

**Table S2** Baseline Characteristics for lncRNA EGOT and SERHL

**Table S3** Univariate and multivariate Cox proportional hazards regression analysis of cirrhotic HCC

**Table S4** Primers for qRT-PCR analysis

**Table S5** HCC-specific differentially expressed lncRNAs from GSE17967

**Table S6** HCC-specific differentially expressed mRNAs from GSE17967

**Table S7** HCC-specific differentially expressed intersection miRNAs from GSE21362 and GSE63046

**Table S8** Long-term outcomes for lncRNA EGOT and SERHL

**Fig. S1**


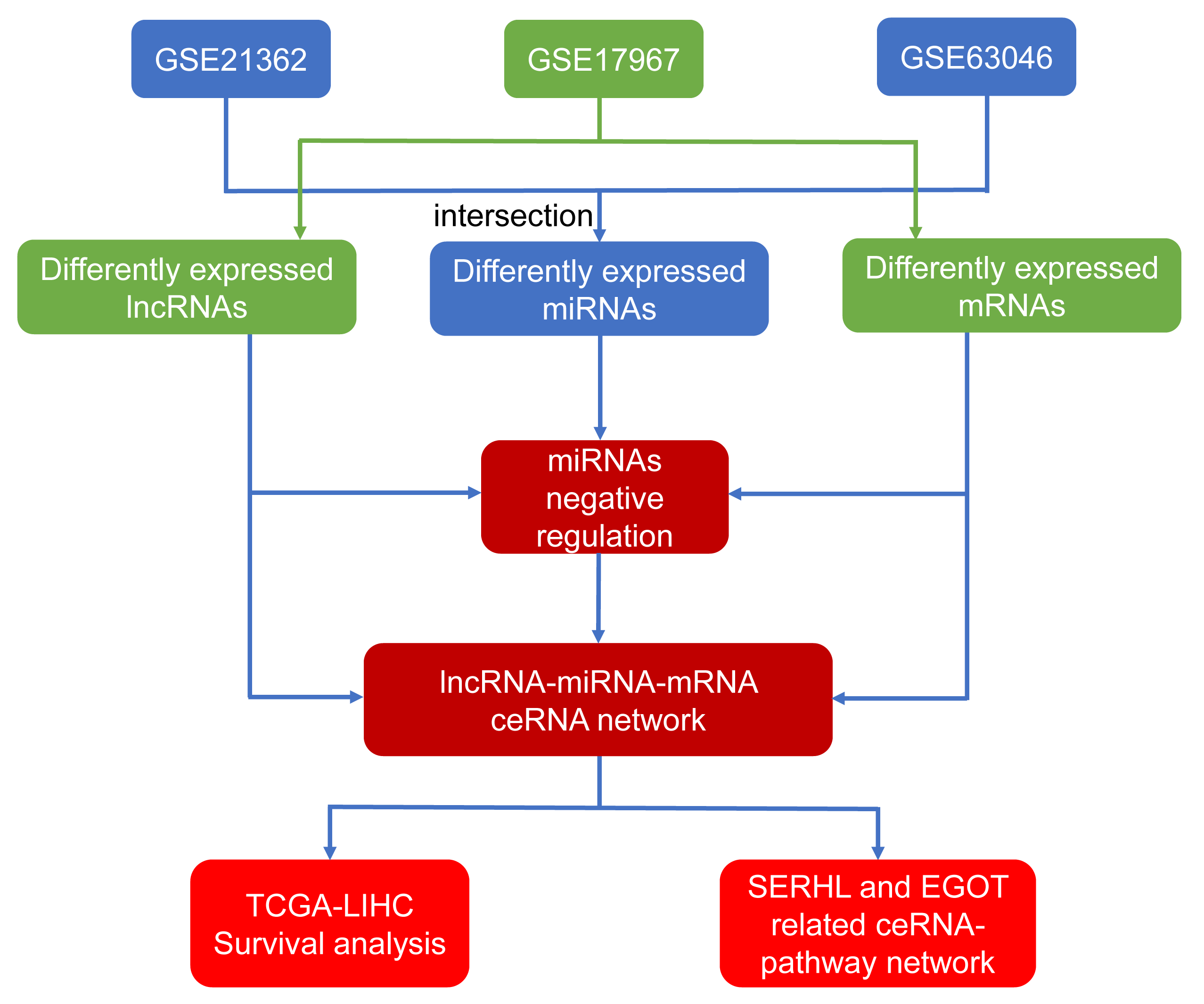


**Fig. S2**


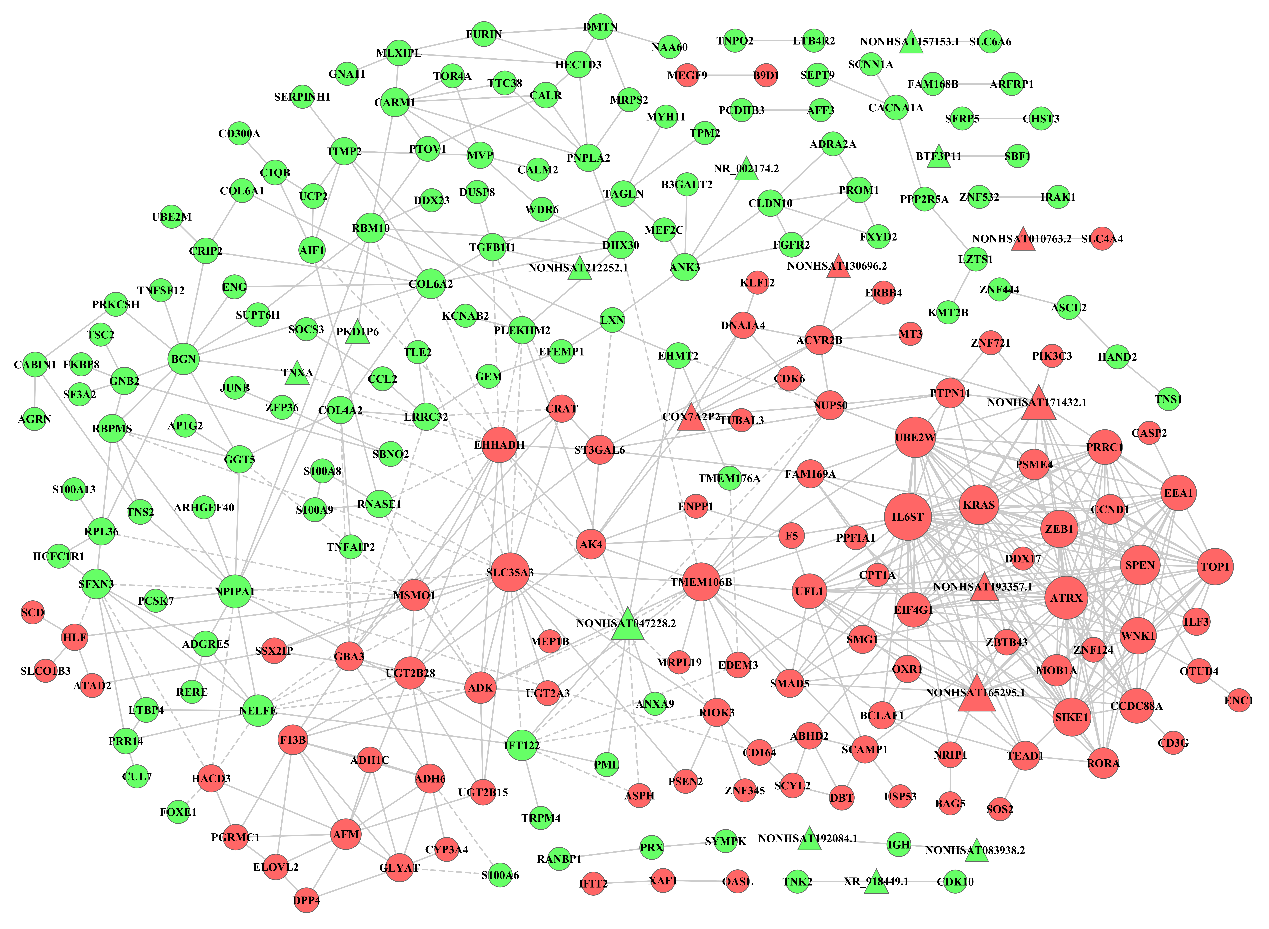


**Fig. S3**


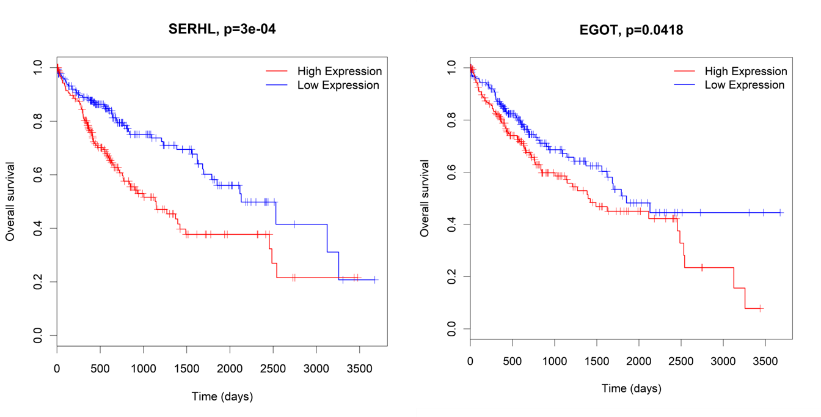
**LncRNAs**

**miRNAs**


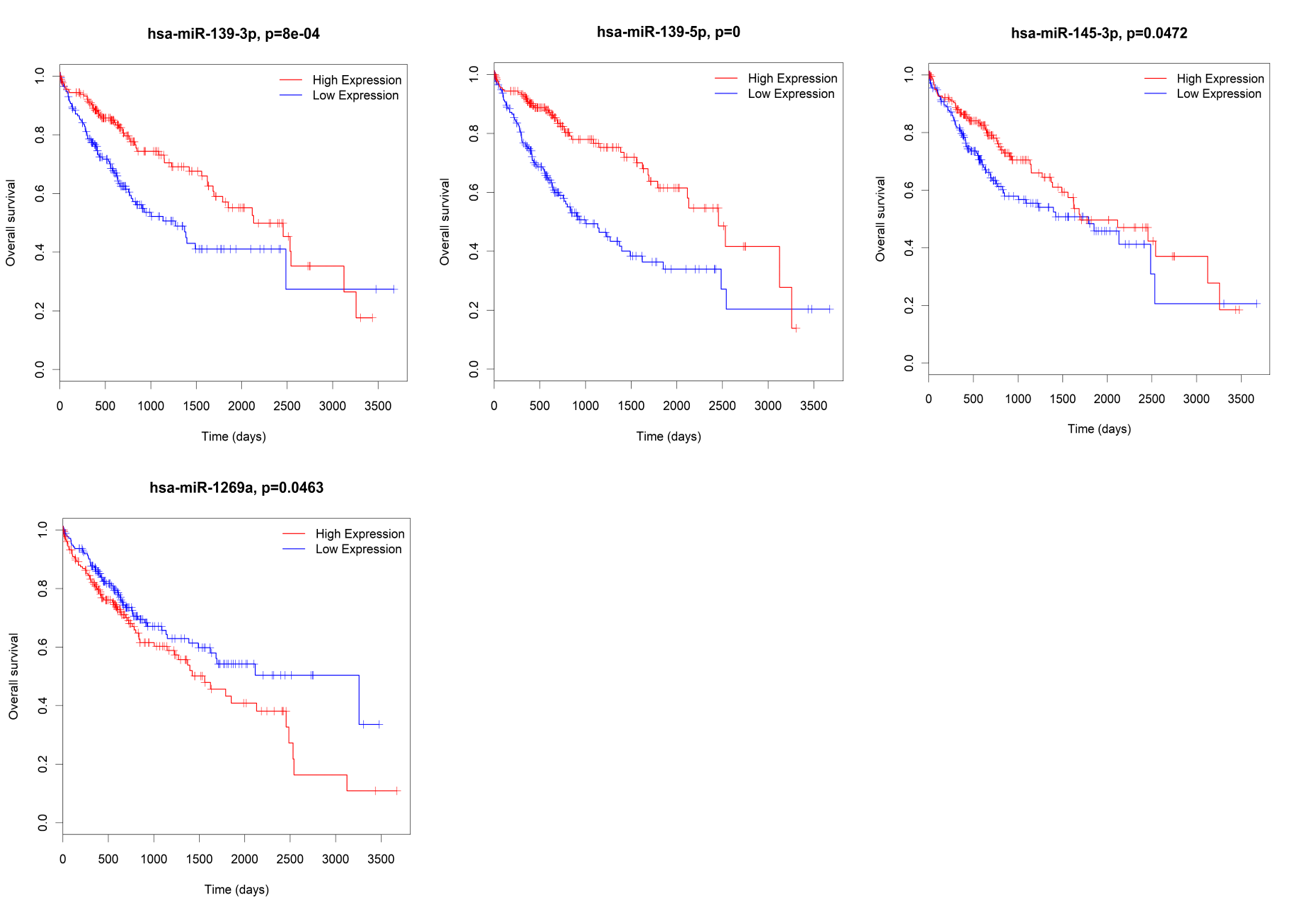


**mRNAs**


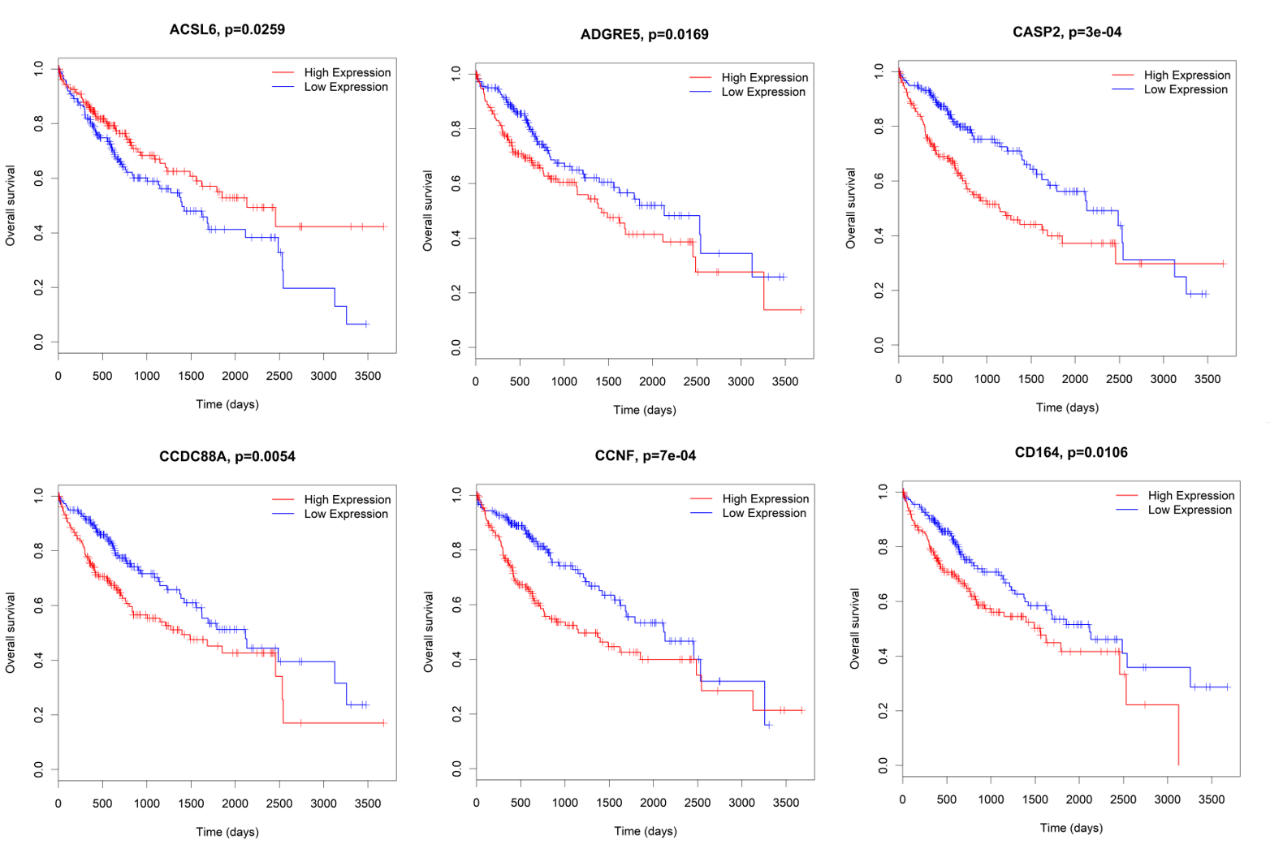


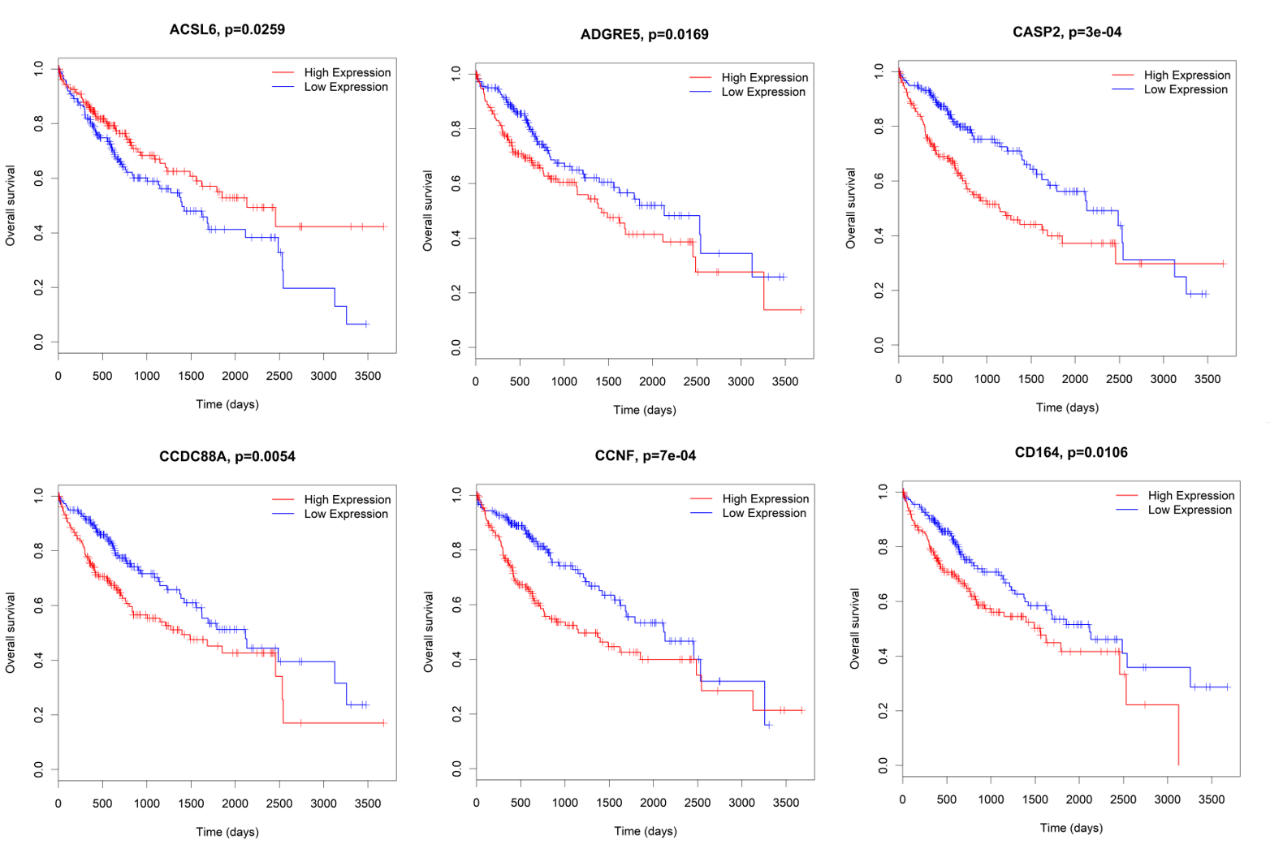


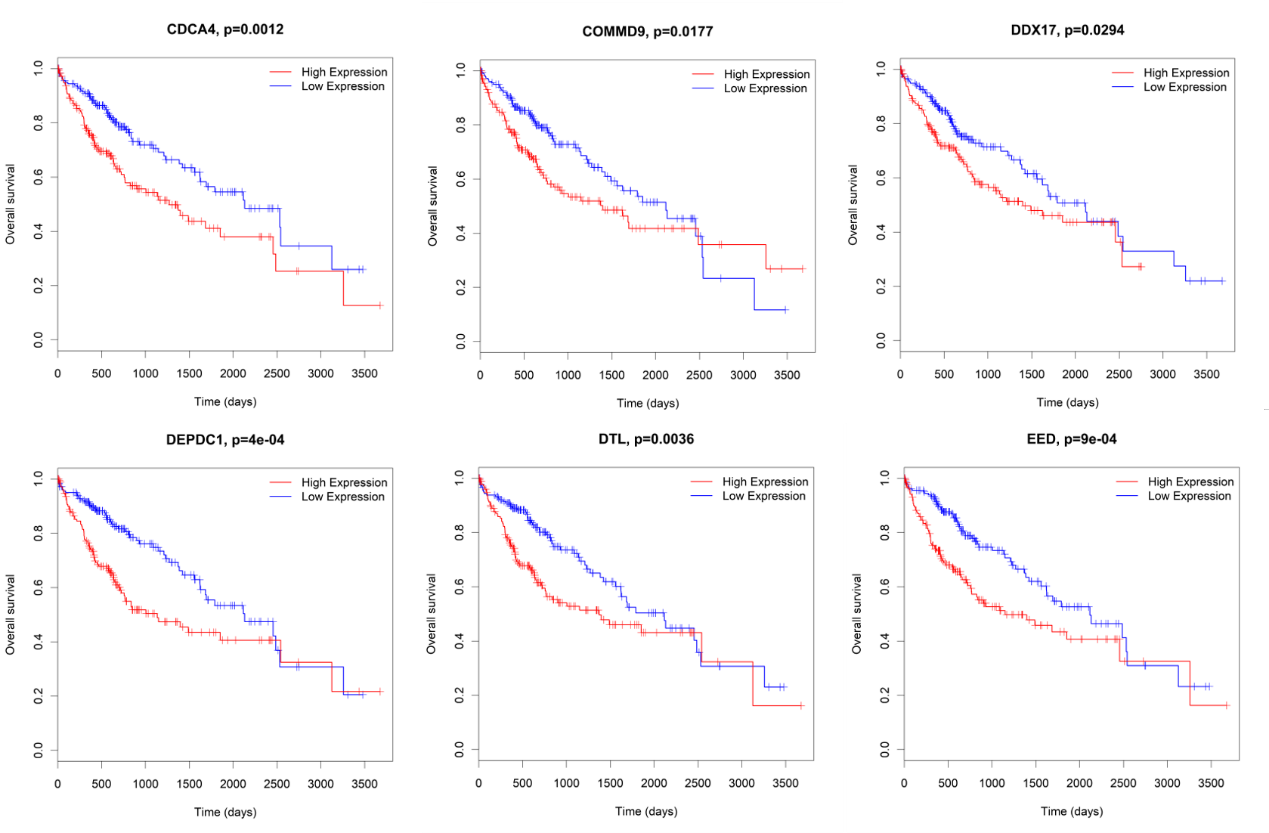


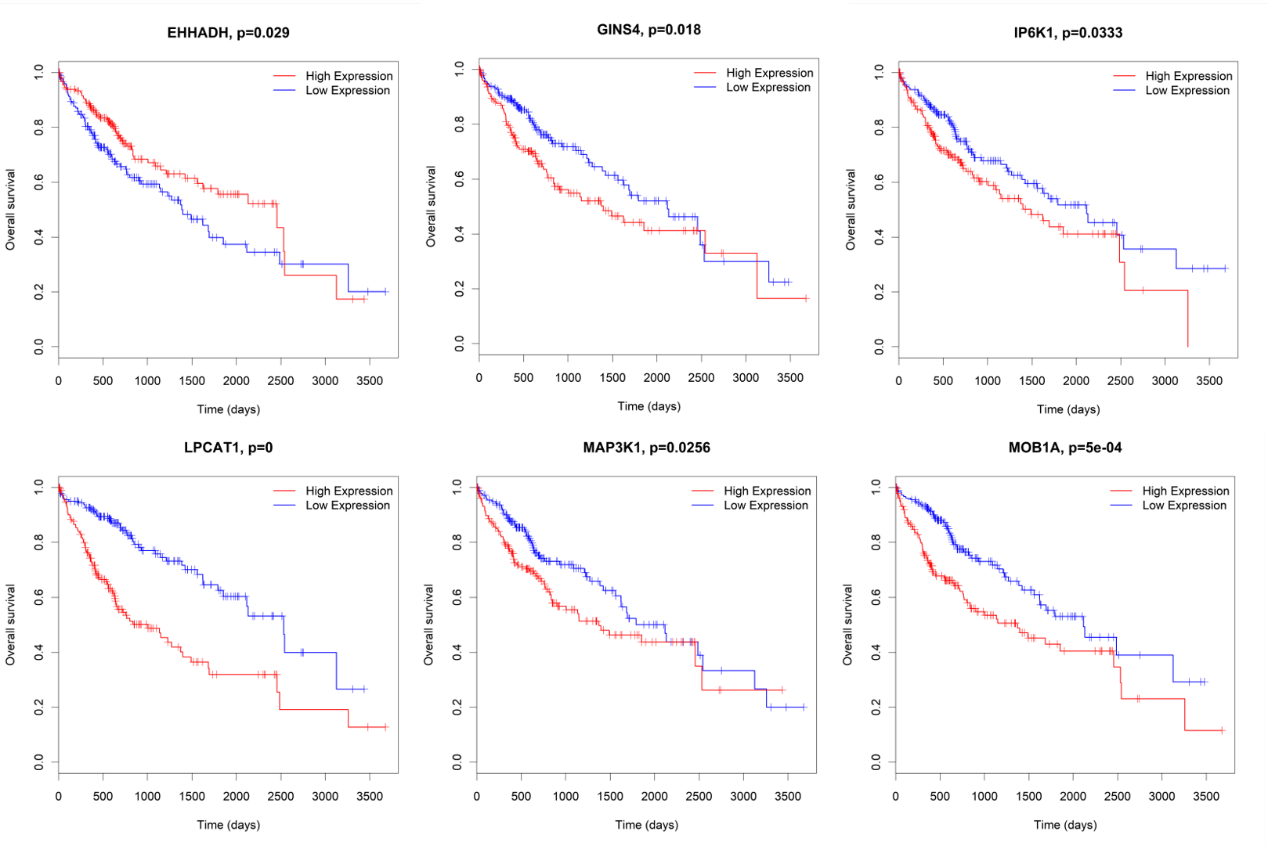


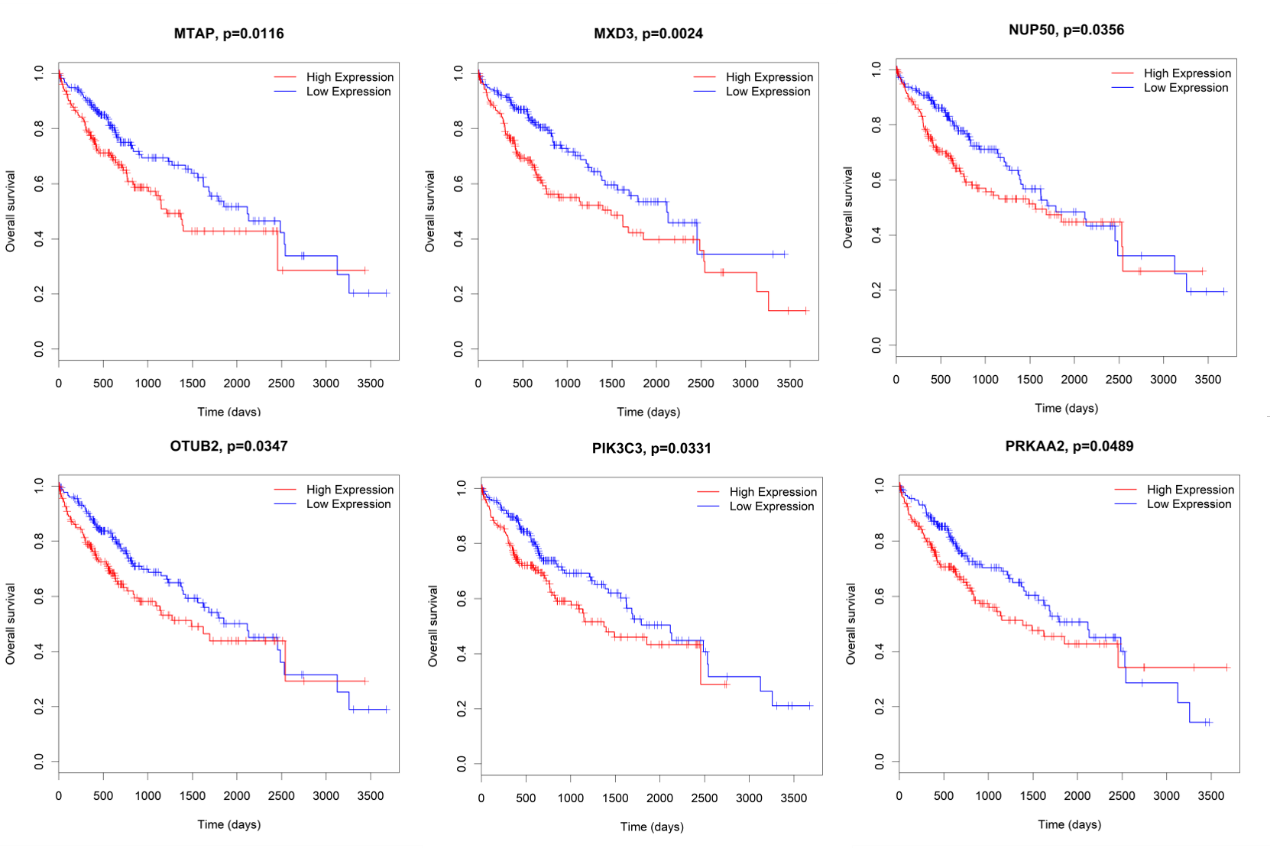


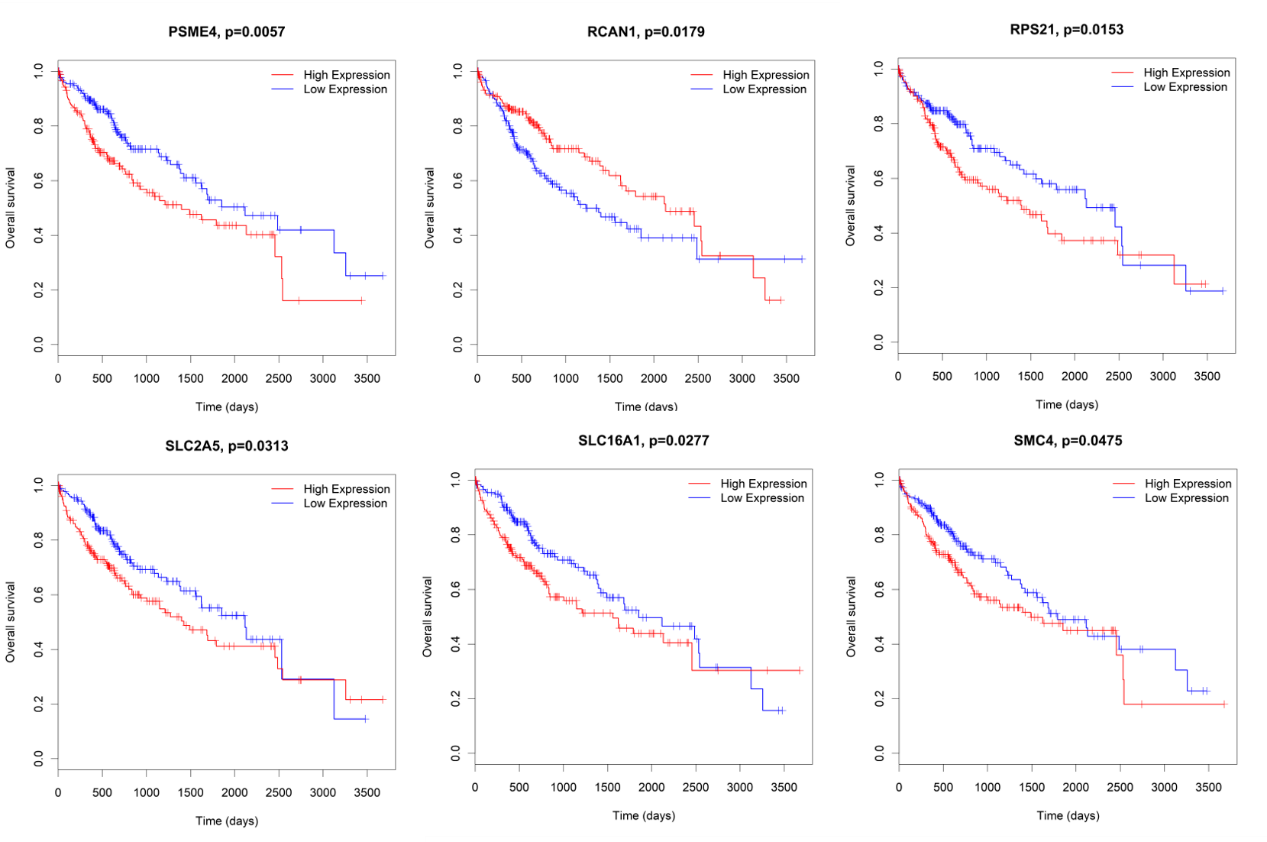


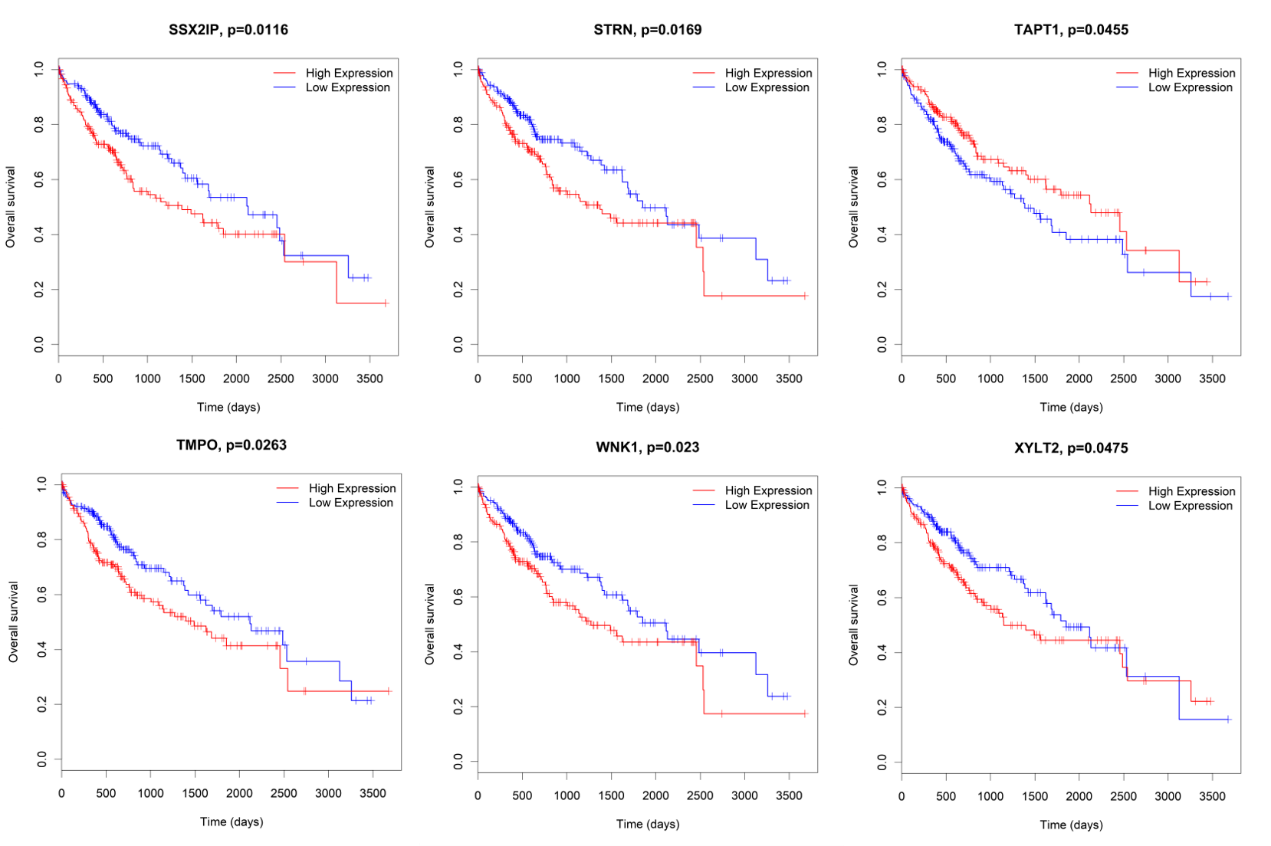


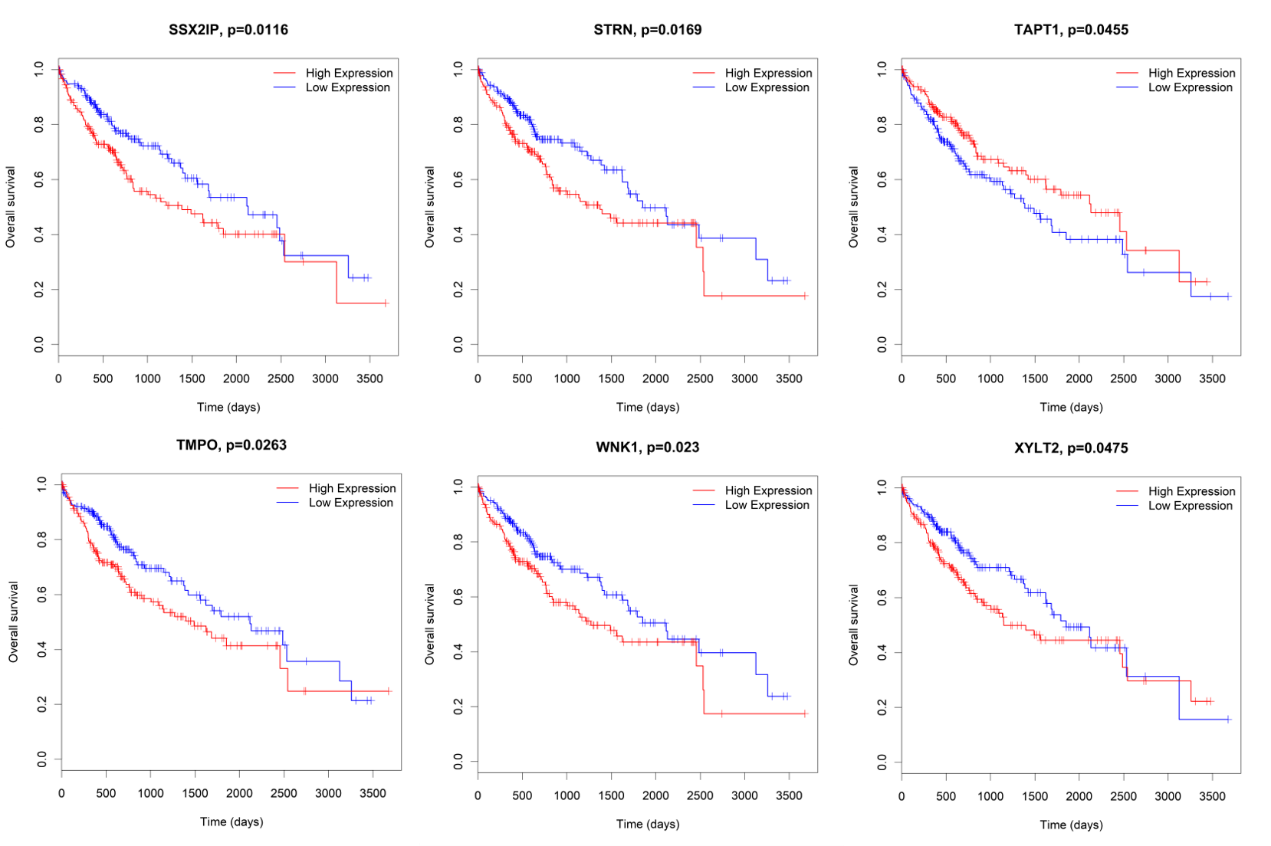


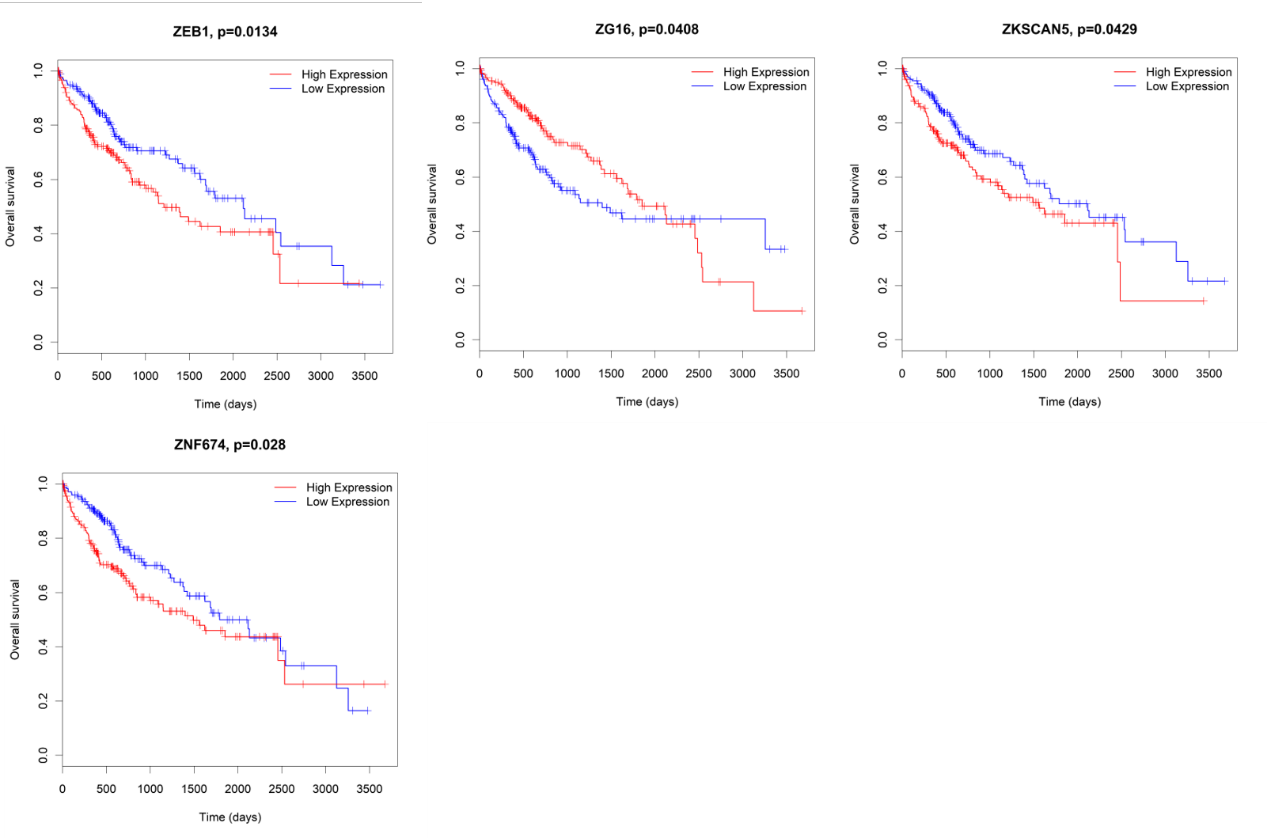


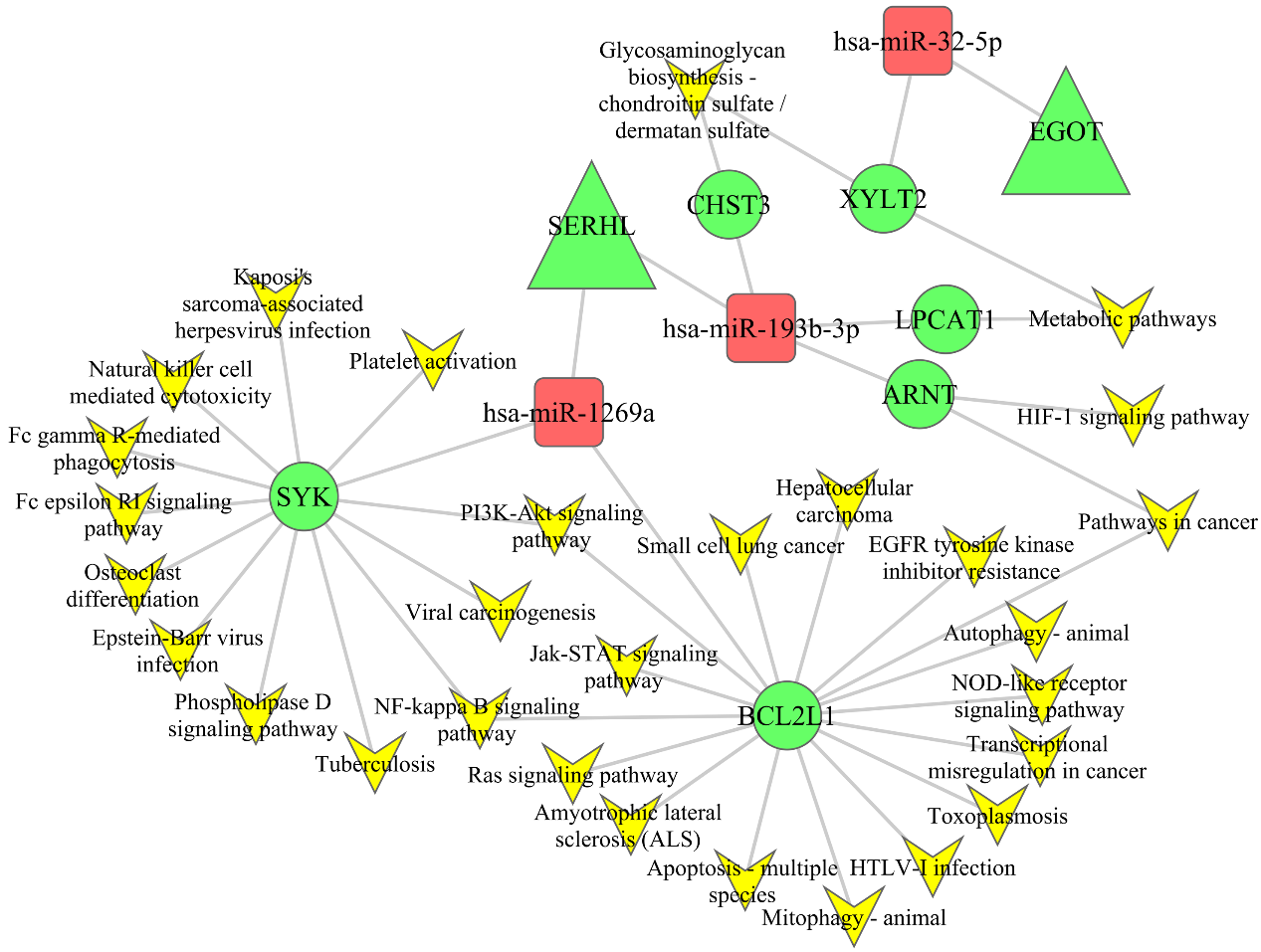
**Fig. S4**


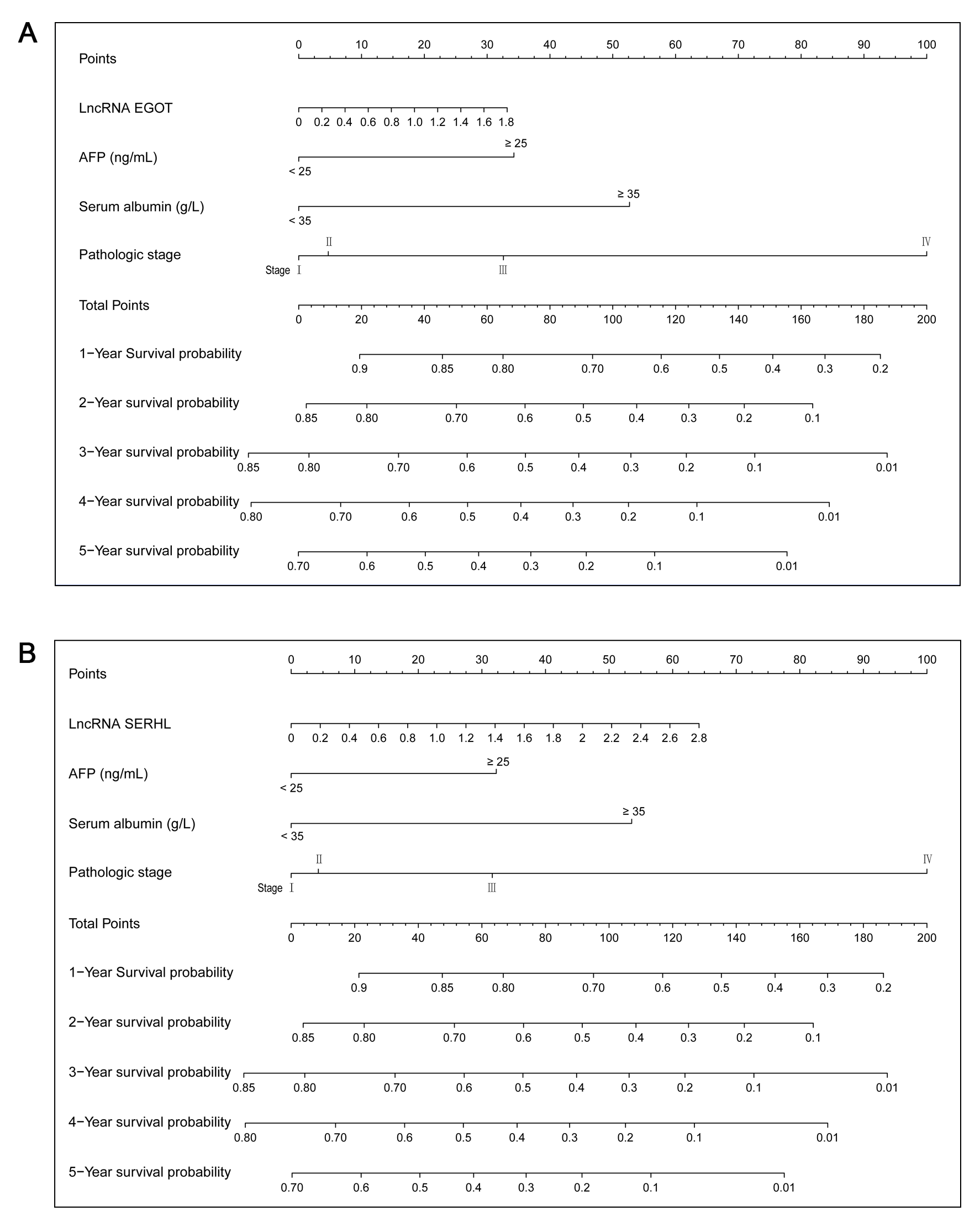
**Fig. S5**

**Table S1 (GSE21362)**

| **!Sample_title** | **!Sample_geo_accession** | **!Sample_source_name_ch1** | **!Sample_characteristics_ch1** | **!Sample_characteristics_ch1** | **ID_REF** |
| --- | --- | --- | --- | --- | --- |
| HCC1-T | GSM533698 | Hepatocellular Carcinoma in Milan Criteria Cases with Mild Liver Cirrhosis | tissue type: Tumor | sample id: HCC1 | GSM533698 |
| HCC2-T | GSM533699 | Hepatocellular Carcinoma in Milan Criteria Cases with Mild Liver Cirrhosis | tissue type: Tumor | sample id: HCC2 | GSM533699 |
| HCC3-T | GSM533700 | Hepatocellular Carcinoma in Milan Criteria Cases with Mild Liver Cirrhosis | tissue type: Tumor | sample id: HCC3 | GSM533700 |
| HCC4-T | GSM533701 | Hepatocellular Carcinoma in Milan Criteria Cases with Mild Liver Cirrhosis | tissue type: Tumor | sample id: HCC4 | GSM533701 |
| HCC5-T | GSM533702 | Hepatocellular Carcinoma in Milan Criteria Cases with Mild Liver Cirrhosis | tissue type: Tumor | sample id: HCC5 | GSM533702 |
| HCC6-T | GSM533703 | Hepatocellular Carcinoma in Milan Criteria Cases with Mild Liver Cirrhosis | tissue type: Tumor | sample id: HCC6 | GSM533703 |
| HCC7-T | GSM533704 | Hepatocellular Carcinoma in Milan Criteria Cases with Mild Liver Cirrhosis | tissue type: Tumor | sample id: HCC7 | GSM533704 |
| HCC8-T | GSM533705 | Hepatocellular Carcinoma in Milan Criteria Cases with Mild Liver Cirrhosis | tissue type: Tumor | sample id: HCC8 | GSM533705 |
| HCC9-T | GSM533706 | Hepatocellular Carcinoma in Milan Criteria Cases with Mild Liver Cirrhosis | tissue type: Tumor | sample id: HCC9 | GSM533706 |
| HCC10-T | GSM533707 | Hepatocellular Carcinoma in Milan Criteria Cases with Mild Liver Cirrhosis | tissue type: Tumor | sample id: HCC10 | GSM533707 |
| HCC11-T | GSM533708 | Hepatocellular Carcinoma in Milan Criteria Cases with Mild Liver Cirrhosis | tissue type: Tumor | sample id: HCC11 | GSM533708 |
| HCC12-T | GSM533709 | Hepatocellular Carcinoma in Milan Criteria Cases with Mild Liver Cirrhosis | tissue type: Tumor | sample id: HCC12 | GSM533709 |
| HCC13-T | GSM533710 | Hepatocellular Carcinoma in Milan Criteria Cases with Mild Liver Cirrhosis | tissue type: Tumor | sample id: HCC13 | GSM533710 |
| HCC14-T | GSM533711 | Hepatocellular Carcinoma in Milan Criteria Cases with Mild Liver Cirrhosis | tissue type: Tumor | sample id: HCC14 | GSM533711 |
| HCC15-T | GSM533712 | Hepatocellular Carcinoma in Milan Criteria Cases with Mild Liver Cirrhosis | tissue type: Tumor | sample id: HCC15 | GSM533712 |
| HCC16-T | GSM533713 | Hepatocellular Carcinoma in Milan Criteria Cases with Mild Liver Cirrhosis | tissue type: Tumor | sample id: HCC16 | GSM533713 |
| HCC17-T | GSM533714 | Hepatocellular Carcinoma in Milan Criteria Cases with Mild Liver Cirrhosis | tissue type: Tumor | sample id: HCC17 | GSM533714 |
| HCC18-T | GSM533715 | Hepatocellular Carcinoma in Milan Criteria Cases with Mild Liver Cirrhosis | tissue type: Tumor | sample id: HCC18 | GSM533715 |
| HCC19-T | GSM533716 | Hepatocellular Carcinoma in Milan Criteria Cases with Mild Liver Cirrhosis | tissue type: Tumor | sample id: HCC19 | GSM533716 |
| HCC20-T | GSM533717 | Hepatocellular Carcinoma in Milan Criteria Cases with Mild Liver Cirrhosis | tissue type: Tumor | sample id: HCC20 | GSM533717 |
| HCC21-T | GSM533718 | Hepatocellular Carcinoma in Milan Criteria Cases with Mild Liver Cirrhosis | tissue type: Tumor | sample id: HCC21 | GSM533718 |
| HCC22-T | GSM533719 | Hepatocellular Carcinoma in Milan Criteria Cases with Mild Liver Cirrhosis | tissue type: Tumor | sample id: HCC22 | GSM533719 |
| HCC23-T | GSM533720 | Hepatocellular Carcinoma in Milan Criteria Cases with Mild Liver Cirrhosis | tissue type: Tumor | sample id: HCC23 | GSM533720 |
| HCC24-T | GSM533721 | Hepatocellular Carcinoma in Milan Criteria Cases with Mild Liver Cirrhosis | tissue type: Tumor | sample id: HCC24 | GSM533721 |
| HCC25-T | GSM533722 | Hepatocellular Carcinoma in Milan Criteria Cases with Mild Liver Cirrhosis | tissue type: Tumor | sample id: HCC25 | GSM533722 |
| HCC26-T | GSM533723 | Hepatocellular Carcinoma in Milan Criteria Cases with Mild Liver Cirrhosis | tissue type: Tumor | sample id: HCC26 | GSM533723 |
| HCC27-T | GSM533724 | Hepatocellular Carcinoma in Milan Criteria Cases with Mild Liver Cirrhosis | tissue type: Tumor | sample id: HCC27 | GSM533724 |
| HCC28-T | GSM533725 | Hepatocellular Carcinoma in Milan Criteria Cases with Mild Liver Cirrhosis | tissue type: Tumor | sample id: HCC28 | GSM533725 |
| HCC29-T | GSM533726 | Hepatocellular Carcinoma in Milan Criteria Cases with Mild Liver Cirrhosis | tissue type: Tumor | sample id: HCC29 | GSM533726 |
| HCC30-T | GSM533727 | Hepatocellular Carcinoma in Milan Criteria Cases with Mild Liver Cirrhosis | tissue type: Tumor | sample id: HCC30 | GSM533727 |
| HCC31-T | GSM533728 | Hepatocellular Carcinoma in Milan Criteria Cases with Mild Liver Cirrhosis | tissue type: Tumor | sample id: HCC31 | GSM533728 |
| HCC32-T | GSM533729 | Hepatocellular Carcinoma in Milan Criteria Cases with Mild Liver Cirrhosis | tissue type: Tumor | sample id: HCC32 | GSM533729 |
| HCC33-T | GSM533730 | Hepatocellular Carcinoma in Milan Criteria Cases with Mild Liver Cirrhosis | tissue type: Tumor | sample id: HCC33 | GSM533730 |
| HCC34-T | GSM533731 | Hepatocellular Carcinoma in Milan Criteria Cases with Mild Liver Cirrhosis | tissue type: Tumor | sample id: HCC34 | GSM533731 |
| HCC35-T | GSM533732 | Hepatocellular Carcinoma in Milan Criteria Cases with Mild Liver Cirrhosis | tissue type: Tumor | sample id: HCC35 | GSM533732 |
| HCC36-T | GSM533733 | Hepatocellular Carcinoma in Milan Criteria Cases with Mild Liver Cirrhosis | tissue type: Tumor | sample id: HCC36 | GSM533733 |
| HCC37-T | GSM533734 | Hepatocellular Carcinoma in Milan Criteria Cases with Mild Liver Cirrhosis | tissue type: Tumor | sample id: HCC37 | GSM533734 |
| HCC38-T | GSM533735 | Hepatocellular Carcinoma in Milan Criteria Cases with Mild Liver Cirrhosis | tissue type: Tumor | sample id: HCC38 | GSM533735 |
| HCC39-T | GSM533736 | Hepatocellular Carcinoma in Milan Criteria Cases with Mild Liver Cirrhosis | tissue type: Tumor | sample id: HCC39 | GSM533736 |
| HCC40-T | GSM533737 | Hepatocellular Carcinoma in Milan Criteria Cases with Mild Liver Cirrhosis | tissue type: Tumor | sample id: HCC40 | GSM533737 |
| HCC41-T | GSM533738 | Hepatocellular Carcinoma in Milan Criteria Cases with Mild Liver Cirrhosis | tissue type: Tumor | sample id: HCC41 | GSM533738 |
| HCC42-T | GSM533739 | Hepatocellular Carcinoma in Milan Criteria Cases with Mild Liver Cirrhosis | tissue type: Tumor | sample id: HCC42 | GSM533739 |
| HCC43-T | GSM533740 | Hepatocellular Carcinoma in Milan Criteria Cases with Mild Liver Cirrhosis | tissue type: Tumor | sample id: HCC43 | GSM533740 |
| HCC44-T | GSM533741 | Hepatocellular Carcinoma in Milan Criteria Cases with Mild Liver Cirrhosis | tissue type: Tumor | sample id: HCC44 | GSM533741 |
| HCC45-T | GSM533742 | Hepatocellular Carcinoma in Milan Criteria Cases with Mild Liver Cirrhosis | tissue type: Tumor | sample id: HCC45 | GSM533742 |
| HCC46-T | GSM533743 | Hepatocellular Carcinoma in Milan Criteria Cases with Mild Liver Cirrhosis | tissue type: Tumor | sample id: HCC46 | GSM533743 |
| HCC47-T | GSM533744 | Hepatocellular Carcinoma in Milan Criteria Cases with Mild Liver Cirrhosis | tissue type: Tumor | sample id: HCC47 | GSM533744 |
| HCC48-T | GSM533745 | Hepatocellular Carcinoma in Milan Criteria Cases with Mild Liver Cirrhosis | tissue type: Tumor | sample id: HCC48 | GSM533745 |
| HCC49-T | GSM533746 | Hepatocellular Carcinoma in Milan Criteria Cases with Mild Liver Cirrhosis | tissue type: Tumor | sample id: HCC49 | GSM533746 |
| HCC50-T | GSM533747 | Hepatocellular Carcinoma in Milan Criteria Cases with Mild Liver Cirrhosis | tissue type: Tumor | sample id: HCC50 | GSM533747 |
| HCC51-T | GSM533748 | Hepatocellular Carcinoma in Milan Criteria Cases with Mild Liver Cirrhosis | tissue type: Tumor | sample id: HCC51 | GSM533748 |
| HCC52-T | GSM533749 | Hepatocellular Carcinoma in Milan Criteria Cases with Mild Liver Cirrhosis | tissue type: Tumor | sample id: HCC52 | GSM533749 |
| HCC53-T | GSM533750 | Hepatocellular Carcinoma in Milan Criteria Cases with Mild Liver Cirrhosis | tissue type: Tumor | sample id: HCC53 | GSM533750 |
| HCC54-T | GSM533751 | Hepatocellular Carcinoma in Milan Criteria Cases with Mild Liver Cirrhosis | tissue type: Tumor | sample id: HCC54 | GSM533751 |
| HCC55-T | GSM533752 | Hepatocellular Carcinoma in Milan Criteria Cases with Mild Liver Cirrhosis | tissue type: Tumor | sample id: HCC55 | GSM533752 |
| HCC56-T | GSM533753 | Hepatocellular Carcinoma in Milan Criteria Cases with Mild Liver Cirrhosis | tissue type: Tumor | sample id: HCC56 | GSM533753 |
| HCC57-T | GSM533754 | Hepatocellular Carcinoma in Milan Criteria Cases with Mild Liver Cirrhosis | tissue type: Tumor | sample id: HCC57 | GSM533754 |
| HCC58-T | GSM533755 | Hepatocellular Carcinoma in Milan Criteria Cases with Mild Liver Cirrhosis | tissue type: Tumor | sample id: HCC58 | GSM533755 |
| HCC59-T | GSM533756 | Hepatocellular Carcinoma in Milan Criteria Cases with Mild Liver Cirrhosis | tissue type: Tumor | sample id: HCC59 | GSM533756 |
| HCC60-T | GSM533757 | Hepatocellular Carcinoma in Milan Criteria Cases with Mild Liver Cirrhosis | tissue type: Tumor | sample id: HCC60 | GSM533757 |
| HCC61-T | GSM533758 | Hepatocellular Carcinoma in Milan Criteria Cases with Mild Liver Cirrhosis | tissue type: Tumor | sample id: HCC61 | GSM533758 |
| HCC62-T | GSM533759 | Hepatocellular Carcinoma in Milan Criteria Cases with Mild Liver Cirrhosis | tissue type: Tumor | sample id: HCC62 | GSM533759 |
| HCC63-T | GSM533760 | Hepatocellular Carcinoma in Milan Criteria Cases with Mild Liver Cirrhosis | tissue type: Tumor | sample id: HCC63 | GSM533760 |
| HCC64-T | GSM533761 | Hepatocellular Carcinoma in Milan Criteria Cases with Mild Liver Cirrhosis | tissue type: Tumor | sample id: HCC64 | GSM533761 |
| HCC65-T | GSM533762 | Hepatocellular Carcinoma in Milan Criteria Cases with Mild Liver Cirrhosis | tissue type: Tumor | sample id: HCC65 | GSM533762 |
| HCC66-T | GSM533763 | Hepatocellular Carcinoma in Milan Criteria Cases with Mild Liver Cirrhosis | tissue type: Tumor | sample id: HCC66 | GSM533763 |
| HCC67-T | GSM533764 | Hepatocellular Carcinoma in Milan Criteria Cases with Mild Liver Cirrhosis | tissue type: Tumor | sample id: HCC67 | GSM533764 |
| HCC68-T | GSM533765 | Hepatocellular Carcinoma in Milan Criteria Cases with Mild Liver Cirrhosis | tissue type: Tumor | sample id: HCC68 | GSM533765 |
| HCC69-T | GSM533766 | Hepatocellular Carcinoma in Milan Criteria Cases with Mild Liver Cirrhosis | tissue type: Tumor | sample id: HCC69 | GSM533766 |
| HCC70-T | GSM533767 | Hepatocellular Carcinoma in Milan Criteria Cases with Mild Liver Cirrhosis | tissue type: Tumor | sample id: HCC70 | GSM533767 |
| HCC71-T | GSM533768 | Hepatocellular Carcinoma in Milan Criteria Cases with Mild Liver Cirrhosis | tissue type: Tumor | sample id: HCC71 | GSM533768 |
| HCC72-T | GSM533769 | Hepatocellular Carcinoma in Milan Criteria Cases with Mild Liver Cirrhosis | tissue type: Tumor | sample id: HCC72 | GSM533769 |
| HCC73-T | GSM533770 | Hepatocellular Carcinoma in Milan Criteria Cases with Mild Liver Cirrhosis | tissue type: Tumor | sample id: HCC73 | GSM533770 |
| HCC1-N | GSM533771 | Hepatocellular Carcinoma in Milan Criteria Cases with Mild Liver Cirrhosis | tissue type: Non-tumor | sample id: HCC1 | GSM533771 |
| HCC2-N | GSM533772 | Hepatocellular Carcinoma in Milan Criteria Cases with Mild Liver Cirrhosis | tissue type: Non-tumor | sample id: HCC2 | GSM533772 |
| HCC3-N | GSM533773 | Hepatocellular Carcinoma in Milan Criteria Cases with Mild Liver Cirrhosis | tissue type: Non-tumor | sample id: HCC3 | GSM533773 |
| HCC4-N | GSM533774 | Hepatocellular Carcinoma in Milan Criteria Cases with Mild Liver Cirrhosis | tissue type: Non-tumor | sample id: HCC4 | GSM533774 |
| HCC5-N | GSM533775 | Hepatocellular Carcinoma in Milan Criteria Cases with Mild Liver Cirrhosis | tissue type: Non-tumor | sample id: HCC5 | GSM533775 |
| HCC6-N | GSM533776 | Hepatocellular Carcinoma in Milan Criteria Cases with Mild Liver Cirrhosis | tissue type: Non-tumor | sample id: HCC6 | GSM533776 |
| HCC7-N | GSM533777 | Hepatocellular Carcinoma in Milan Criteria Cases with Mild Liver Cirrhosis | tissue type: Non-tumor | sample id: HCC7 | GSM533777 |
| HCC8-N | GSM533778 | Hepatocellular Carcinoma in Milan Criteria Cases with Mild Liver Cirrhosis | tissue type: Non-tumor | sample id: HCC8 | GSM533778 |
| HCC9-N | GSM533779 | Hepatocellular Carcinoma in Milan Criteria Cases with Mild Liver Cirrhosis | tissue type: Non-tumor | sample id: HCC9 | GSM533779 |
| HCC10-N | GSM533780 | Hepatocellular Carcinoma in Milan Criteria Cases with Mild Liver Cirrhosis | tissue type: Non-tumor | sample id: HCC10 | GSM533780 |
| HCC11-N | GSM533781 | Hepatocellular Carcinoma in Milan Criteria Cases with Mild Liver Cirrhosis | tissue type: Non-tumor | sample id: HCC11 | GSM533781 |
| HCC12-N | GSM533782 | Hepatocellular Carcinoma in Milan Criteria Cases with Mild Liver Cirrhosis | tissue type: Non-tumor | sample id: HCC12 | GSM533782 |
| HCC13-N | GSM533783 | Hepatocellular Carcinoma in Milan Criteria Cases with Mild Liver Cirrhosis | tissue type: Non-tumor | sample id: HCC13 | GSM533783 |
| HCC14-N | GSM533784 | Hepatocellular Carcinoma in Milan Criteria Cases with Mild Liver Cirrhosis | tissue type: Non-tumor | sample id: HCC14 | GSM533784 |
| HCC15-N | GSM533785 | Hepatocellular Carcinoma in Milan Criteria Cases with Mild Liver Cirrhosis | tissue type: Non-tumor | sample id: HCC15 | GSM533785 |
| HCC16-N | GSM533786 | Hepatocellular Carcinoma in Milan Criteria Cases with Mild Liver Cirrhosis | tissue type: Non-tumor | sample id: HCC16 | GSM533786 |
| HCC17-N | GSM533787 | Hepatocellular Carcinoma in Milan Criteria Cases with Mild Liver Cirrhosis | tissue type: Non-tumor | sample id: HCC17 | GSM533787 |
| HCC18-N | GSM533788 | Hepatocellular Carcinoma in Milan Criteria Cases with Mild Liver Cirrhosis | tissue type: Non-tumor | sample id: HCC18 | GSM533788 |
| HCC19-N | GSM533789 | Hepatocellular Carcinoma in Milan Criteria Cases with Mild Liver Cirrhosis | tissue type: Non-tumor | sample id: HCC19 | GSM533789 |
| HCC20-N | GSM533790 | Hepatocellular Carcinoma in Milan Criteria Cases with Mild Liver Cirrhosis | tissue type: Non-tumor | sample id: HCC20 | GSM533790 |
| HCC21-N | GSM533791 | Hepatocellular Carcinoma in Milan Criteria Cases with Mild Liver Cirrhosis | tissue type: Non-tumor | sample id: HCC21 | GSM533791 |
| HCC22-N | GSM533792 | Hepatocellular Carcinoma in Milan Criteria Cases with Mild Liver Cirrhosis | tissue type: Non-tumor | sample id: HCC22 | GSM533792 |
| HCC23-N | GSM533793 | Hepatocellular Carcinoma in Milan Criteria Cases with Mild Liver Cirrhosis | tissue type: Non-tumor | sample id: HCC23 | GSM533793 |
| HCC24-N | GSM533794 | Hepatocellular Carcinoma in Milan Criteria Cases with Mild Liver Cirrhosis | tissue type: Non-tumor | sample id: HCC24 | GSM533794 |
| HCC25-N | GSM533795 | Hepatocellular Carcinoma in Milan Criteria Cases with Mild Liver Cirrhosis | tissue type: Non-tumor | sample id: HCC25 | GSM533795 |
| HCC26-N | GSM533796 | Hepatocellular Carcinoma in Milan Criteria Cases with Mild Liver Cirrhosis | tissue type: Non-tumor | sample id: HCC26 | GSM533796 |
| HCC27-N | GSM533797 | Hepatocellular Carcinoma in Milan Criteria Cases with Mild Liver Cirrhosis | tissue type: Non-tumor | sample id: HCC27 | GSM533797 |
| HCC28-N | GSM533798 | Hepatocellular Carcinoma in Milan Criteria Cases with Mild Liver Cirrhosis | tissue type: Non-tumor | sample id: HCC28 | GSM533798 |
| HCC29-N | GSM533799 | Hepatocellular Carcinoma in Milan Criteria Cases with Mild Liver Cirrhosis | tissue type: Non-tumor | sample id: HCC29 | GSM533799 |
| HCC30-N | GSM533800 | Hepatocellular Carcinoma in Milan Criteria Cases with Mild Liver Cirrhosis | tissue type: Non-tumor | sample id: HCC30 | GSM533800 |
| HCC31-N | GSM533801 | Hepatocellular Carcinoma in Milan Criteria Cases with Mild Liver Cirrhosis | tissue type: Non-tumor | sample id: HCC31 | GSM533801 |
| HCC32-N | GSM533802 | Hepatocellular Carcinoma in Milan Criteria Cases with Mild Liver Cirrhosis | tissue type: Non-tumor | sample id: HCC32 | GSM533802 |
| HCC33-N | GSM533803 | Hepatocellular Carcinoma in Milan Criteria Cases with Mild Liver Cirrhosis | tissue type: Non-tumor | sample id: HCC33 | GSM533803 |
| HCC34-N | GSM533804 | Hepatocellular Carcinoma in Milan Criteria Cases with Mild Liver Cirrhosis | tissue type: Non-tumor | sample id: HCC34 | GSM533804 |
| HCC35-N | GSM533805 | Hepatocellular Carcinoma in Milan Criteria Cases with Mild Liver Cirrhosis | tissue type: Non-tumor | sample id: HCC35 | GSM533805 |
| HCC36-N | GSM533806 | Hepatocellular Carcinoma in Milan Criteria Cases with Mild Liver Cirrhosis | tissue type: Non-tumor | sample id: HCC36 | GSM533806 |
| HCC37-N | GSM533807 | Hepatocellular Carcinoma in Milan Criteria Cases with Mild Liver Cirrhosis | tissue type: Non-tumor | sample id: HCC37 | GSM533807 |
| HCC38-N | GSM533808 | Hepatocellular Carcinoma in Milan Criteria Cases with Mild Liver Cirrhosis | tissue type: Non-tumor | sample id: HCC38 | GSM533808 |
| HCC39-N | GSM533809 | Hepatocellular Carcinoma in Milan Criteria Cases with Mild Liver Cirrhosis | tissue type: Non-tumor | sample id: HCC39 | GSM533809 |
| HCC40-N | GSM533810 | Hepatocellular Carcinoma in Milan Criteria Cases with Mild Liver Cirrhosis | tissue type: Non-tumor | sample id: HCC40 | GSM533810 |
| HCC41-N | GSM533811 | Hepatocellular Carcinoma in Milan Criteria Cases with Mild Liver Cirrhosis | tissue type: Non-tumor | sample id: HCC41 | GSM533811 |
| HCC42-N | GSM533812 | Hepatocellular Carcinoma in Milan Criteria Cases with Mild Liver Cirrhosis | tissue type: Non-tumor | sample id: HCC42 | GSM533812 |
| HCC43-N | GSM533813 | Hepatocellular Carcinoma in Milan Criteria Cases with Mild Liver Cirrhosis | tissue type: Non-tumor | sample id: HCC43 | GSM533813 |
| HCC44-N | GSM533814 | Hepatocellular Carcinoma in Milan Criteria Cases with Mild Liver Cirrhosis | tissue type: Non-tumor | sample id: HCC44 | GSM533814 |
| HCC45-N | GSM533815 | Hepatocellular Carcinoma in Milan Criteria Cases with Mild Liver Cirrhosis | tissue type: Non-tumor | sample id: HCC45 | GSM533815 |
| HCC46-N | GSM533816 | Hepatocellular Carcinoma in Milan Criteria Cases with Mild Liver Cirrhosis | tissue type: Non-tumor | sample id: HCC46 | GSM533816 |
| HCC47-N | GSM533817 | Hepatocellular Carcinoma in Milan Criteria Cases with Mild Liver Cirrhosis | tissue type: Non-tumor | sample id: HCC47 | GSM533817 |
| HCC48-N | GSM533818 | Hepatocellular Carcinoma in Milan Criteria Cases with Mild Liver Cirrhosis | tissue type: Non-tumor | sample id: HCC48 | GSM533818 |
| HCC49-N | GSM533819 | Hepatocellular Carcinoma in Milan Criteria Cases with Mild Liver Cirrhosis | tissue type: Non-tumor | sample id: HCC49 | GSM533819 |
| HCC50-N | GSM533820 | Hepatocellular Carcinoma in Milan Criteria Cases with Mild Liver Cirrhosis | tissue type: Non-tumor | sample id: HCC50 | GSM533820 |
| HCC51-N | GSM533821 | Hepatocellular Carcinoma in Milan Criteria Cases with Mild Liver Cirrhosis | tissue type: Non-tumor | sample id: HCC51 | GSM533821 |
| HCC52-N | GSM533822 | Hepatocellular Carcinoma in Milan Criteria Cases with Mild Liver Cirrhosis | tissue type: Non-tumor | sample id: HCC52 | GSM533822 |
| HCC53-N | GSM533823 | Hepatocellular Carcinoma in Milan Criteria Cases with Mild Liver Cirrhosis | tissue type: Non-tumor | sample id: HCC53 | GSM533823 |
| HCC54-N | GSM533824 | Hepatocellular Carcinoma in Milan Criteria Cases with Mild Liver Cirrhosis | tissue type: Non-tumor | sample id: HCC54 | GSM533824 |
| HCC55-N | GSM533825 | Hepatocellular Carcinoma in Milan Criteria Cases with Mild Liver Cirrhosis | tissue type: Non-tumor | sample id: HCC55 | GSM533825 |
| HCC56-N | GSM533826 | Hepatocellular Carcinoma in Milan Criteria Cases with Mild Liver Cirrhosis | tissue type: Non-tumor | sample id: HCC56 | GSM533826 |
| HCC57-N | GSM533827 | Hepatocellular Carcinoma in Milan Criteria Cases with Mild Liver Cirrhosis | tissue type: Non-tumor | sample id: HCC57 | GSM533827 |
| HCC58-N | GSM533828 | Hepatocellular Carcinoma in Milan Criteria Cases with Mild Liver Cirrhosis | tissue type: Non-tumor | sample id: HCC58 | GSM533828 |
| HCC59-N | GSM533829 | Hepatocellular Carcinoma in Milan Criteria Cases with Mild Liver Cirrhosis | tissue type: Non-tumor | sample id: HCC59 | GSM533829 |
| HCC60-N | GSM533830 | Hepatocellular Carcinoma in Milan Criteria Cases with Mild Liver Cirrhosis | tissue type: Non-tumor | sample id: HCC60 | GSM533830 |
| HCC61-N | GSM533831 | Hepatocellular Carcinoma in Milan Criteria Cases with Mild Liver Cirrhosis | tissue type: Non-tumor | sample id: HCC61 | GSM533831 |
| HCC62-N | GSM533832 | Hepatocellular Carcinoma in Milan Criteria Cases with Mild Liver Cirrhosis | tissue type: Non-tumor | sample id: HCC62 | GSM533832 |
| HCC63-N | GSM533833 | Hepatocellular Carcinoma in Milan Criteria Cases with Mild Liver Cirrhosis | tissue type: Non-tumor | sample id: HCC63 | GSM533833 |
| HCC64-N | GSM533834 | Hepatocellular Carcinoma in Milan Criteria Cases with Mild Liver Cirrhosis | tissue type: Non-tumor | sample id: HCC64 | GSM533834 |
| HCC65-N | GSM533835 | Hepatocellular Carcinoma in Milan Criteria Cases with Mild Liver Cirrhosis | tissue type: Non-tumor | sample id: HCC65 | GSM533835 |
| HCC66-N | GSM533836 | Hepatocellular Carcinoma in Milan Criteria Cases with Mild Liver Cirrhosis | tissue type: Non-tumor | sample id: HCC66 | GSM533836 |
| HCC67-N | GSM533837 | Hepatocellular Carcinoma in Milan Criteria Cases with Mild Liver Cirrhosis | tissue type: Non-tumor | sample id: HCC67 | GSM533837 |
| HCC68-N | GSM533838 | Hepatocellular Carcinoma in Milan Criteria Cases with Mild Liver Cirrhosis | tissue type: Non-tumor | sample id: HCC68 | GSM533838 |
| HCC69-N | GSM533839 | Hepatocellular Carcinoma in Milan Criteria Cases with Mild Liver Cirrhosis | tissue type: Non-tumor | sample id: HCC69 | GSM533839 |
| HCC70-N | GSM533840 | Hepatocellular Carcinoma in Milan Criteria Cases with Mild Liver Cirrhosis | tissue type: Non-tumor | sample id: HCC70 | GSM533840 |
| HCC71-N | GSM533841 | Hepatocellular Carcinoma in Milan Criteria Cases with Mild Liver Cirrhosis | tissue type: Non-tumor | sample id: HCC71 | GSM533841 |
| HCC72-N | GSM533842 | Hepatocellular Carcinoma in Milan Criteria Cases with Mild Liver Cirrhosis | tissue type: Non-tumor | sample id: HCC72 | GSM533842 |
| HCC73-N | GSM533843 | Hepatocellular Carcinoma in Milan Criteria Cases with Mild Liver Cirrhosis | tissue type: Non-tumor | sample id: HCC73 | GSM533843 |

**Table S1(GSE17967)**

| **!Sample_title** | **!Sample_geo_accession** | **!Sample_source_name_ch1** | **!Sample_characteristics_ch1** | **!Sample_characteristics_ch1** | **ID_REF** |
| --- | --- | --- | --- | --- | --- |
| HCV+cirrhosis with HCC 1 | GSM449731 | liver tissue | disease state: HCV + cirrhosis with HCC | tissue: liver | GSM449731 |
| HCV+cirrhosis with HCC 2 | GSM449732 | liver tissue | disease state: HCV + cirrhosis with HCC | tissue: liver | GSM449732 |
| HCV+cirrhosis without HCC 1 | GSM449733 | liver tissue | disease state: HCV + cirrhosis without HCC | tissue: liver | GSM449733 |
| HCV+cirrhosis with HCC 3 | GSM449734 | liver tissue | disease state: HCV + cirrhosis with HCC | tissue: liver | GSM449734 |
| HCV+cirrhosis without HCC 2 | GSM449735 | liver tissue | disease state: HCV + cirrhosis without HCC | tissue: liver | GSM449735 |
| HCV+cirrhosis with HCC 4 | GSM449736 | liver tissue | disease state: HCV + cirrhosis with HCC | tissue: liver | GSM449736 |
| HCV+cirrhosis without HCC 3 | GSM449737 | liver tissue | disease state: HCV + cirrhosis without HCC | tissue: liver | GSM449737 |
| HCV+cirrhosis without HCC 4 | GSM449738 | liver tissue | disease state: HCV + cirrhosis without HCC | tissue: liver | GSM449738 |
| HCV+cirrhosis without HCC 5 | GSM449739 | liver tissue | disease state: HCV + cirrhosis without HCC | tissue: liver | GSM449739 |
| HCV+cirrhosis without HCC 6 | GSM449740 | liver tissue | disease state: HCV + cirrhosis without HCC | tissue: liver | GSM449740 |
| HCV+cirrhosis without HCC 7 | GSM449741 | liver tissue | disease state: HCV + cirrhosis without HCC | tissue: liver | GSM449741 |
| HCV+cirrhosis without HCC 8 | GSM449742 | liver tissue | disease state: HCV + cirrhosis without HCC | tissue: liver | GSM449742 |
| HCV+cirrhosis without HCC 9 | GSM449743 | liver tissue | disease state: HCV + cirrhosis without HCC | tissue: liver | GSM449743 |
| HCV+cirrhosis without HCC 10 | GSM449744 | liver tissue | disease state: HCV + cirrhosis without HCC | tissue: liver | GSM449744 |
| HCV+cirrhosis without HCC 11 | GSM449745 | liver tissue | disease state: HCV + cirrhosis without HCC | tissue: liver | GSM449745 |
| HCV+cirrhosis without HCC 12 | GSM449746 | liver tissue | disease state: HCV + cirrhosis without HCC | tissue: liver | GSM449746 |
| HCV+cirrhosis with HCC 5 | GSM449747 | liver tissue | disease state: HCV + cirrhosis with HCC | tissue: liver | GSM449747 |
| HCV+cirrhosis without HCC 13 | GSM449748 | liver tissue | disease state: HCV + cirrhosis without HCC | tissue: liver | GSM449748 |
| HCV+cirrhosis without HCC 14 | GSM449749 | liver tissue | disease state: HCV + cirrhosis without HCC | tissue: liver | GSM449749 |
| HCV+cirrhosis with HCC 6 | GSM449750 | liver tissue | disease state: HCV + cirrhosis with HCC | tissue: liver | GSM449750 |
| HCV+cirrhosis without HCC 15 | GSM449751 | liver tissue | disease state: HCV + cirrhosis without HCC | tissue: liver | GSM449751 |
| HCV+cirrhosis with HCC 7 | GSM449752 | liver tissue | disease state: HCV + cirrhosis with HCC | tissue: liver | GSM449752 |
| HCV+cirrhosis with HCC 8 | GSM449753 | liver tissue | disease state: HCV + cirrhosis with HCC | tissue: liver | GSM449753 |
| HCV+cirrhosis without HCC 16 | GSM449754 | liver tissue | disease state: HCV + cirrhosis without HCC | tissue: liver | GSM449754 |
| HCV+cirrhosis without HCC 17 | GSM449755 | liver tissue | disease state: HCV + cirrhosis without HCC | tissue: liver | GSM449755 |
| HCV+cirrhosis without HCC 18 | GSM449756 | liver tissue | disease state: HCV + cirrhosis without HCC | tissue: liver | GSM449756 |
| HCV+cirrhosis with HCC 9 | GSM449757 | liver tissue | disease state: HCV + cirrhosis with HCC | tissue: liver | GSM449757 |
| HCV+cirrhosis with HCC 10 | GSM449758 | liver tissue | disease state: HCV + cirrhosis with HCC | tissue: liver | GSM449758 |
| HCV+cirrhosis without HCC 19 | GSM449759 | liver tissue | disease state: HCV + cirrhosis without HCC | tissue: liver | GSM449759 |
| HCV+cirrhosis without HCC 20 | GSM449760 | liver tissue | disease state: HCV + cirrhosis without HCC | tissue: liver | GSM449760 |
| HCV+cirrhosis without HCC 21 | GSM449761 | liver tissue | disease state: HCV + cirrhosis without HCC | tissue: liver | GSM449761 |
| HCV+cirrhosis without HCC 22 | GSM449762 | liver tissue | disease state: HCV + cirrhosis without HCC | tissue: liver | GSM449762 |
| HCV+cirrhosis without HCC 23 | GSM449763 | liver tissue | disease state: HCV + cirrhosis without HCC | tissue: liver | GSM449763 |
| HCV+cirrhosis without HCC 24 | GSM449764 | liver tissue | disease state: HCV + cirrhosis without HCC | tissue: liver | GSM449764 |
| HCV+cirrhosis without HCC 25 | GSM449765 | liver tissue | disease state: HCV + cirrhosis without HCC | tissue: liver | GSM449765 |
| HCV+cirrhosis without HCC 26 | GSM449766 | liver tissue | disease state: HCV + cirrhosis without HCC | tissue: liver | GSM449766 |
| HCV+cirrhosis without HCC 27 | GSM449767 | liver tissue | disease state: HCV + cirrhosis without HCC | tissue: liver | GSM449767 |
| HCV+cirrhosis without HCC 28 | GSM449768 | liver tissue | disease state: HCV + cirrhosis without HCC | tissue: liver | GSM449768 |
| HCV+cirrhosis without HCC 29 | GSM449769 | liver tissue | disease state: HCV + cirrhosis without HCC | tissue: liver | GSM449769 |
| HCV+cirrhosis without HCC 30 | GSM449770 | liver tissue | disease state: HCV + cirrhosis without HCC | tissue: liver | GSM449770 |
| HCV+cirrhosis with HCC 11 | GSM449771 | liver tissue | disease state: HCV + cirrhosis with HCC | tissue: liver | GSM449771 |
| HCV+cirrhosis with HCC 12 | GSM449772 | liver tissue | disease state: HCV + cirrhosis with HCC | tissue: liver | GSM449772 |
| HCV+cirrhosis without HCC 31 | GSM449773 | liver tissue | disease state: HCV + cirrhosis without HCC | tissue: liver | GSM449773 |
| HCV+cirrhosis without HCC 32 | GSM449774 | liver tissue | disease state: HCV + cirrhosis without HCC | tissue: liver | GSM449774 |
| HCV+cirrhosis without HCC 33 | GSM449775 | liver tissue | disease state: HCV + cirrhosis without HCC | tissue: liver | GSM449775 |
| HCV+cirrhosis with HCC 13 | GSM449776 | liver tissue | disease state: HCV + cirrhosis with HCC | tissue: liver | GSM449776 |
| HCV+cirrhosis with HCC 14 | GSM449777 | liver tissue | disease state: HCV + cirrhosis with HCC | tissue: liver | GSM449777 |
| HCV+cirrhosis without HCC 34 | GSM449778 | liver tissue | disease state: HCV + cirrhosis without HCC | tissue: liver | GSM449778 |
| HCV+cirrhosis with HCC 15 | GSM449779 | liver tissue | disease state: HCV + cirrhosis with HCC | tissue: liver | GSM449779 |
| HCV+cirrhosis without HCC 35 | GSM449780 | liver tissue | disease state: HCV + cirrhosis without HCC | tissue: liver | GSM449780 |
| HCV+cirrhosis without HCC 36 | GSM449781 | liver tissue | disease state: HCV + cirrhosis without HCC | tissue: liver | GSM449781 |
| HCV+cirrhosis without HCC 37 | GSM449782 | liver tissue | disease state: HCV + cirrhosis without HCC | tissue: liver | GSM449782 |
| HCV+cirrhosis without HCC 38 | GSM449783 | liver tissue | disease state: HCV + cirrhosis without HCC | tissue: liver | GSM449783 |
| HCV+cirrhosis without HCC 39 | GSM449784 | liver tissue | disease state: HCV + cirrhosis without HCC | tissue: liver | GSM449784 |
| HCV+cirrhosis without HCC 40 | GSM449785 | liver tissue | disease state: HCV + cirrhosis without HCC | tissue: liver | GSM449785 |
| HCV+cirrhosis without HCC 41 | GSM449786 | liver tissue | disease state: HCV + cirrhosis without HCC | tissue: liver | GSM449786 |
| HCV+cirrhosis without HCC 42 | GSM449787 | liver tissue | disease state: HCV + cirrhosis without HCC | tissue: liver | GSM449787 |
| HCV+cirrhosis without HCC 43 | GSM449788 | liver tissue | disease state: HCV + cirrhosis without HCC | tissue: liver | GSM449788 |
| HCV+cirrhosis without HCC 44 | GSM449789 | liver tissue | disease state: HCV + cirrhosis without HCC | tissue: liver | GSM449789 |
| HCV+cirrhosis without HCC 45 | GSM449790 | liver tissue | disease state: HCV + cirrhosis without HCC | tissue: liver | GSM449790 |
| HCV+cirrhosis without HCC 46 | GSM449791 | liver tissue | disease state: HCV + cirrhosis without HCC | tissue: liver | GSM449791 |
| HCV+cirrhosis without HCC 47 | GSM449792 | liver tissue | disease state: HCV + cirrhosis without HCC | tissue: liver | GSM449792 |
| HCV+cirrhosis with HCC 16 | GSM449793 | liver tissue | disease state: HCV + cirrhosis with HCC | tissue: liver | GSM449793 |

**Table S1 (GSE63046)**

| **!Sample_title** | **!Sample_geo_accession** | **!Sample_source_name_ch1** | **!Sample_characteristics_ch1** | **!Sample_characteristics_ch1** | **!Sample_characteristics_ch1** | **!Sample_characteristics_ch1** | **!Sample_characteristics_ch1** | **ID_REF** |
| --- | --- | --- | --- | --- | --- | --- | --- | --- |
| 1001N | GSM1538840 | Unaffected liver cells tissue adjacent to tumor | type of liver tissue: Unaffected tissue adjacent to tumor | cirrhosis: yes | hbv/hcv infection: yes | gender: M | age: 44 | GSM1538840 |
| 1001T | GSM1538841 | Hepatocellular carcinoma liver cells | type of liver tissue: Hepatocellular carcinoma tumor | cirrhosis: yes | hbv/hcv infection: yes | gender: M | age: 44 | GSM1538841 |
| 1009N | GSM1538842 | Unaffected liver cells tissue adjacent to tumor | type of liver tissue: Unaffected tissue adjacent to tumor | cirrhosis: no | hbv/hcv infection: yes | gender: M | age: 52 | GSM1538842 |
| 1009T | GSM1538843 | Hepatocellular carcinoma liver cells | type of liver tissue: Hepatocellular carcinoma tumor | cirrhosis: no | hbv/hcv infection: yes | gender: M | age: 51 | GSM1538843 |
| 1010N | GSM1538844 | Unaffected liver cells tissue adjacent to tumor | type of liver tissue: Unaffected tissue adjacent to tumor | cirrhosis: no | hbv/hcv infection: yes | gender: M | age: 75 | GSM1538844 |
| 1010T | GSM1538845 | Hepatocellular carcinoma liver cells | type of liver tissue: Hepatocellular carcinoma tumor | cirrhosis: no | hbv/hcv infection: yes | gender: M | age: 75 | GSM1538845 |
| 1012N | GSM1538846 | Unaffected liver cells tissue adjacent to tumor | type of liver tissue: Unaffected tissue adjacent to tumor | cirrhosis: yes | hbv/hcv infection: no | gender: M | age: 65 | GSM1538846 |
| 1012T | GSM1538847 | Hepatocellular carcinoma liver cells | type of liver tissue: Hepatocellular carcinoma tumor | cirrhosis: yes | hbv/hcv infection: no | gender: M | age: 65 | GSM1538847 |
| 1013N | GSM1538848 | Unaffected liver cells tissue adjacent to tumor | type of liver tissue: Unaffected tissue adjacent to tumor | cirrhosis: yes | hbv/hcv infection: yes | gender: M | age: 58 | GSM1538848 |
| 1013T | GSM1538849 | Hepatocellular carcinoma liver cells | type of liver tissue: Hepatocellular carcinoma tumor | cirrhosis: yes | hbv/hcv infection: yes | gender: M | age: 58 | GSM1538849 |
| 1014N | GSM1538850 | Unaffected liver cells tissue adjacent to tumor | type of liver tissue: Unaffected tissue adjacent to tumor | cirrhosis: yes | hbv/hcv infection: yes | gender: F | age: 60 | GSM1538850 |
| 1014T | GSM1538851 | Hepatocellular carcinoma liver cells | type of liver tissue: Hepatocellular carcinoma tumor | cirrhosis: yes | hbv/hcv infection: yes | gender: F | age: 60 | GSM1538851 |
| 1015N | GSM1538852 | Unaffected liver cells tissue adjacent to tumor | type of liver tissue: Unaffected tissue adjacent to tumor | cirrhosis: no | hbv/hcv infection: yes | gender: M | age: 77 | GSM1538852 |
| 1015T | GSM1538853 | Hepatocellular carcinoma liver cells | type of liver tissue: Hepatocellular carcinoma tumor | cirrhosis: no | hbv/hcv infection: yes | gender: M | age: 77 | GSM1538853 |
| 1016N | GSM1538854 | Unaffected liver cells tissue adjacent to tumor | type of liver tissue: Unaffected tissue adjacent to tumor | cirrhosis: no | hbv/hcv infection: no | gender: M | age: 72 | GSM1538854 |
| 1016T | GSM1538855 | Hepatocellular carcinoma liver cells | type of liver tissue: Hepatocellular carcinoma tumor | cirrhosis: no | hbv/hcv infection: no | gender: M | age: 72 | GSM1538855 |
| 1018N | GSM1538856 | Unaffected liver cells tissue adjacent to tumor | type of liver tissue: Unaffected tissue adjacent to tumor | cirrhosis: yes | hbv/hcv infection: yes | gender: M | age: 55 | GSM1538856 |
| 1018T | GSM1538857 | Hepatocellular carcinoma liver cells | type of liver tissue: Hepatocellular carcinoma tumor | cirrhosis: yes | hbv/hcv infection: yes | gender: M | age: 55 | GSM1538857 |
| 1022N | GSM1538858 | Unaffected liver cells tissue adjacent to tumor | type of liver tissue: Unaffected tissue adjacent to tumor | cirrhosis: yes | hbv/hcv infection: yes | gender: M | age: 58 | GSM1538858 |
| 1022T | GSM1538859 | Hepatocellular carcinoma liver cells | type of liver tissue: Hepatocellular carcinoma tumor | cirrhosis: yes | hbv/hcv infection: yes | gender: M | age: 58 | GSM1538859 |
| 1023N | GSM1538860 | Unaffected liver cells tissue adjacent to tumor | type of liver tissue: Unaffected tissue adjacent to tumor | cirrhosis: no | hbv/hcv infection: yes | gender: M | age: 61 | GSM1538860 |
| 1023T | GSM1538861 | Hepatocellular carcinoma liver cells | type of liver tissue: Hepatocellular carcinoma tumor | cirrhosis: no | hbv/hcv infection: yes | gender: M | age: 61 | GSM1538861 |
| 1025N | GSM1538862 | Unaffected liver cells tissue adjacent to tumor | type of liver tissue: Unaffected tissue adjacent to tumor | cirrhosis: no | hbv/hcv infection: no | gender: M | age: 65 | GSM1538862 |
| 1025T | GSM1538863 | Hepatocellular carcinoma liver cells | type of liver tissue: Hepatocellular carcinoma tumor | cirrhosis: no | hbv/hcv infection: no | gender: M | age: 65 | GSM1538863 |
| 1032N | GSM1538864 | Unaffected liver cells tissue adjacent to tumor | type of liver tissue: Unaffected tissue adjacent to tumor | cirrhosis: yes | hbv/hcv infection: no | gender: M | age: 66 | GSM1538864 |
| 1032T | GSM1538865 | Hepatocellular carcinoma liver cells | type of liver tissue: Hepatocellular carcinoma tumor | cirrhosis: yes | hbv/hcv infection: no | gender: M | age: 66 | GSM1538865 |
| 1034N | GSM1538866 | Unaffected liver cells tissue adjacent to tumor | type of liver tissue: Unaffected tissue adjacent to tumor | cirrhosis: yes | hbv/hcv infection: yes | gender: F | age: 60 | GSM1538866 |
| 1034T | GSM1538867 | Hepatocellular carcinoma liver cells | type of liver tissue: Hepatocellular carcinoma tumor | cirrhosis: yes | hbv/hcv infection: yes | gender: F | age: 60 | GSM1538867 |
| 1037N | GSM1538868 | Unaffected liver cells tissue adjacent to tumor | type of liver tissue: Unaffected tissue adjacent to tumor | cirrhosis: no | hbv/hcv infection: no | gender: M | age: 65 | GSM1538868 |
| 1037T | GSM1538869 | Hepatocellular carcinoma liver cells | type of liver tissue: Hepatocellular carcinoma tumor | cirrhosis: no | hbv/hcv infection: no | gender: M | age: 65 | GSM1538869 |
| 1038N | GSM1538870 | Unaffected liver cells tissue adjacent to tumor | type of liver tissue: Unaffected tissue adjacent to tumor | cirrhosis: yes | hbv/hcv infection: yes | gender: F | age: 31 | GSM1538870 |
| 1038T | GSM1538871 | Hepatocellular carcinoma liver cells | type of liver tissue: Hepatocellular carcinoma tumor | cirrhosis: yes | hbv/hcv infection: yes | gender: F | age: 31 | GSM1538871 |
| 1039N | GSM1538872 | Unaffected liver cells tissue adjacent to tumor | type of liver tissue: Unaffected tissue adjacent to tumor | cirrhosis: yes | hbv/hcv infection: yes | gender: M | age: 67 | GSM1538872 |
| 1039T | GSM1538873 | Hepatocellular carcinoma liver cells | type of liver tissue: Hepatocellular carcinoma tumor | cirrhosis: yes | hbv/hcv infection: yes | gender: M | age: 67 | GSM1538873 |
| 1041N | GSM1538874 | Unaffected liver cells tissue adjacent to tumor | type of liver tissue: Unaffected tissue adjacent to tumor | cirrhosis: yes | hbv/hcv infection: no | gender: M | age: 65 | GSM1538874 |
| 1041T | GSM1538875 | Hepatocellular carcinoma liver cells | type of liver tissue: Hepatocellular carcinoma tumor | cirrhosis: yes | hbv/hcv infection: no | gender: M | age: 65 | GSM1538875 |
| 1086N | GSM1538876 | Unaffected liver cells tissue adjacent to tumor | type of liver tissue: Unaffected tissue adjacent to tumor | cirrhosis: yes | hbv/hcv infection: yes | gender: M | age: 55 | GSM1538876 |
| 1086T | GSM1538877 | Hepatocellular carcinoma liver cells | type of liver tissue: Hepatocellular carcinoma tumor | cirrhosis: yes | hbv/hcv infection: yes | gender: M | age: 55 | GSM1538877 |
| 1096N | GSM1538878 | Unaffected liver cells tissue adjacent to tumor | type of liver tissue: Unaffected tissue adjacent to tumor | cirrhosis: yes | hbv/hcv infection: yes | gender: F | age: 62 | GSM1538878 |
| 1096T | GSM1538879 | Hepatocellular carcinoma liver cells | type of liver tissue: Hepatocellular carcinoma tumor | cirrhosis: yes | hbv/hcv infection: yes | gender: F | age: 62 | GSM1538879 |
| 1097N | GSM1538880 | Unaffected liver cells tissue adjacent to tumor | type of liver tissue: Unaffected tissue adjacent to tumor | cirrhosis: yes | hbv/hcv infection: no | gender: M | age: 45 | GSM1538880 |
| 1097T | GSM1538881 | Hepatocellular carcinoma liver cells | type of liver tissue: Hepatocellular carcinoma tumor | cirrhosis: yes | hbv/hcv infection: no | gender: M | age: 45 | GSM1538881 |
| 1099N | GSM1538882 | Unaffected liver cells tissue adjacent to tumor | type of liver tissue: Unaffected tissue adjacent to tumor | cirrhosis: no | hbv/hcv infection: no | gender: M | age: 74 | GSM1538882 |
| 1099T | GSM1538883 | Hepatocellular carcinoma liver cells | type of liver tissue: Hepatocellular carcinoma tumor | cirrhosis: no | hbv/hcv infection: no | gender: M | age: 74 | GSM1538883 |
| 1114N | GSM1538884 | Unaffected liver cells tissue adjacent to tumor | type of liver tissue: Unaffected tissue adjacent to tumor | cirrhosis: yes | hbv/hcv infection: yes | gender: M | age: 56 | GSM1538884 |
| 1114T | GSM1538885 | Hepatocellular carcinoma liver cells | type of liver tissue: Hepatocellular carcinoma tumor | cirrhosis: yes | hbv/hcv infection: yes | gender: M | age: 56 | GSM1538885 |
| 1143N | GSM1538886 | Unaffected liver cells tissue adjacent to tumor | type of liver tissue: Unaffected tissue adjacent to tumor | cirrhosis: no | hbv/hcv infection: no | gender: F | age: 74 | GSM1538886 |
| 1143T | GSM1538887 | Hepatocellular carcinoma liver cells | type of liver tissue: Hepatocellular carcinoma tumor | cirrhosis: no | hbv/hcv infection: no | gender: F | age: 74 | GSM1538887 |

**Table S2. Baseline Characteristics for lncRNA EGOT and SERHL (N = 377)**

|  | **EGOT** | | | **SERHL** | | |
| --- | --- | --- | --- | --- | --- | --- |
|  | **High expression**  **(n = 189, 50.1%)** | **Low expression**  **(n = 188, 49.9%)** | ***P* value** | **High expression**  **(n = 245, 99.4%)** | **Low expression**  **(n = 245, 99.4%)** | ***P***  **value** |
| **Demographics** |  |  |  |  |  |  |
| Age at event, years | 61.2 ± 12.2 | 57.7 ± 14.5 | 0.011 | 59.2 ± 13.9 | 59.7 ± 13.2 | 0.692 |
| Male | 122 (64.6%) | 133 (70.7%) | 0.199 | 125 (66.1%) | 130 (69.1%) | 0.532 |
| Height, cm | 166.8 ± 12.4 | 167.9 ± 8.7 | 0.335 | 168.4±9.2 | 166.4±11.8 | 0.090 |
| Weight, kg | 71.5 ± 18.6 | 74.3 ± 20.1 | 0.181 | 72.9±19.3 | 72.9±19.5 | 0.998 |
| **Race** |  |  | 0.133 |  |  | 0.020 |
| White | 99 (52.4%) | 88 (46.8%) |  | 110 (58.2%) | 77 (41.0%) |  |
| African American | 11 (5.8%) | 6 (3.2%) |  | 7 (3.7%) | 10 (5.3%) |  |
| American Indian /Alaska native | 0 (0.0%) | 2 (1.1%) |  | 1 (0.5%) | 1 (0.5%) |  |
| Asian | 76 (40.2%) | 85 (45.2%) |  | 66 (34.9%) | 95 (50.5%) |  |
| Others | 3 (1.6%) | 7 (3.7%) |  | 5 (2.6%) | 5 (2.7%) |  |
| **Pathologic stage** |  |  | 0.512 |  |  | 0.077 |
| Stage Ⅰ | 98 (51.9%) | 101 (53.7%) |  | 90 (47.6%) | 109 (58.0%) |  |
| Stage Ⅱ | 46 (24.3%) | 41 (21.8%) |  | 50 (26.5%) | 37 (19.7%) |  |
| Stage Ⅲ | 44 (23.3%) | 42 (22.3%) |  | 48 (25.4%) | 38 (20.2%) |  |
| Stage Ⅳ | 1 (0.5%) | 4 (2.1%) |  | 1 (0.5%) | 4 (2.1%) |  |
| **Child–Pugh score** |  |  | 0.018 |  |  | 0.370 |
| Class A | 183 (96.8%) | 172 (91.5%) |  | 176 (93.1%) | 179 (95.2%) |  |
| Class B | 5 (2.6%) | 16 (8.5%) |  | 13 (6.9%) | 8 (4.3%) |  |
| Class C | 1 (0.5%) | 0 (0.0%) |  | 0 (0.0%) | 1 (0.5%) |  |
| **Neoplasm histologic grade** |  |  | 0.244 |  |  | 0.233 |
| G1 | 26 (13.8%) | 34 (18.1%) |  | 30 (15.9%) | 30 (16.0%) |  |
| G2 | 100 (52.9%) | 80 (42.6%) |  | 90 (47.6%) | 90 (47.9%) |  |
| G3 | 57 (30.2%) | 67 (35.6%) |  | 66 (34.9%) | 58 (30.9%) |  |
| G4 | 6 (3.2%) | 7 (3.7%) |  | 3 (1.6%) | 10 (5.3%) |  |
| **Ishak fibrosis score** |  |  | 0.202 |  |  | 0.863 |
| 0 - No fibrosis | 127 (67.2%) | 108 (57.4%) |  | 120 (63.5%) | 115 (61.2%) |  |
| 1, 2 - Portal fibrosis | 16 (8.5%) | 15 (8.0%) |  | 16 (8.5%) | 15 (8.0%) |  |
| 3, 4 - Fibrous septa | 10 (5.3%) | 20 (10.6%) |  | 14 (7.4%) | 16 (8.5%) |  |
| 5 - Nodular formation / incomplete cirrhosis | 4 (2.1%) | 5 (2.7%) |  | 3 (1.6%) | 6 (3.2%) |  |
| 6 - Established cirrhosis | 32 (16.9%) | 40 (21.3%) |  | 36 (19.0%) | 36 (19.1%) |  |
| **Vascular invasion** |  |  | 0.945 |  |  | 0.972 |
| Macro | 9 (4.8%) | 8 (4.3%) |  | 9 (4.8%) | 8 (4.3%) |  |
| Micro | 46 (24.3%) | 48 (25.5%) |  | 47 (24.9%) | 47 (25.0%) |  |
| None | 134 (70.9%) | 132 (70.2%) |  | 133 (70.4%) | 133 (70.7%) |  |
| **Residual tumor grade** |  |  | 0.292 |  |  | 0.619 |
| Rx | 17 (9.0%) | 12 (6.4%) |  | 17 (9.0%) | 12 (6.4%) |  |
| R0 | 161 (85.2%) | 169 (89.9%) |  | 163 (86.2%) | 167 (88.8%) |  |
| R1 | 11 (5.8%) | 6 (3.2%) |  | 9 (4.8%) | 8 (4.3%) |  |
| R2 | 0 (0.0%) | 1 (0.5%) |  | 0 (0.0%) | 1 (0.5%) |  |
| **Radiation therapy** | 2 (1.1%) | 7 (3.7%) | 0.175 | 5 (2.6%) | 4 (2.1%) | 1.000 |
| **Neoadjuvant** **therapy** | 1 (0.5%) | 1 (0.5%) | 1.000 | 2 (1.1%) | 0 (0.0%) | 0.481 |
| **Embolization therapy** | 15 (7.9%) | 14 (7.4%) | 0.858 | 18 (9.5%) | 11 (5.9%) | 0.181 |
| **Other malignant tumors** | 23 (12.2%) | 14 (7.4%) | 0.123 | 17 (9.0%) | 20 (10.6%) | 0.592 |
| **Family history of cancer** | 61 (32.3%) | 53 (28.2%) | 0.388 | 59 (31.2%) | 55 (29.3%) | 0.678 |
| **Risk factors** |  |  |  |  |  |  |
| Hepatitis B | 40 (21.2%) | 58 (30.9%) | 0.032 | 33 (17.5%) | 65 (34.6%) | < 0.001 |
| Hepatitis C | 25 (13.2%) | 25 (13.3%) | 0.984 | 29 (15.3%) | 21 (11.2%) | 0.232 |
| Alcohol consumption | 43 (22.8%) | 25 (13.3%) | 0.017 | 45 (23.8%) | 23 (12.2%) | 0.003 |
| Smoking | 7 (3.7%) | 9 (4.8%) | 0.602 | 5 (2.6%) | 11 (5.9%) | 0.123 |
| Hepatic cirrhosis | 2 (1.1%) | 4 (2.1%) | 0.676 | 5 (2.6%) | 1 (0.5%) | 0.219 |
| Non-alcoholic fatty liver disease | 6 (3.2%) | 8 (4.3%) | 0.579 | 8 (4.2%) | 6 (3.2%) | 0.593 |
| Hemochromatosis | 5 (2.6%) | 1 (0.5%) | 0.219 | 3 (1.6%) | 3 (1.6%) | 1.000 |
| Hepatic encephalopathy | 1 (0.5%) | 0 (0.0%) | 1.000 | 0 (0.0%) | 1 (0.5%) | 0.499 |
| Diabetes mellitus | 1 (0.5%) | 0 (0.0%) | 1.000 | 1 (0.5%) | 0 (0.0%) | 1.000 |
| Autoimmune hepatitis | 1 (0.5%) | 0 (0.0%) | 1.000 | 0 (0.0%) | 1 (0.5%) | 0.499 |
| Primary biliary cholangitis | 1 (0.5%) | 0 (0.0%) | 1.000 | 0 (0.0%) | 1 (0.5%) | 0.499 |
| α-1 antitrypsin deficiency | 0 (0.0%) | 1 (0.5%) | 0.499 | 1 (0.5%) | 0 (0.0%) | 1.000 |
| Sarcoidosis | 0 (0.0%) | 1 (0.5%) | 0.499 | 1 (0.5%) | 0 (0.0%) | 1.000 |
| Worker at steel factory | 0 (0.0%) | 1 (0.5%) | 0.499 | 1 (0.5%) | 0 (0.0%) | 1.000 |
| **Biomarkers** |  |  |  |  |  |  |
| AFP, ng/mL | 19.0 (4.5-205.5) | 11.0 (4.0-498.0) | 0.707 | 23.5 (5.0-402.8) | 9.0 (3.0-207.3) | 0.040 |
| Serum albumin, g/L | 4.0 (3.5-4.4) | 4.0 (3.4-4.3) | 0.485 | 4.0 (3.5-4.3) | 4.0 (3.5-4.3) | 0.877 |
| Total bilirubin, mg/dL | 1.2 (1.2-1.3) | 1.2 (1.0-1.2) | 0.022 | 1.2 (1.0-1.3) | 1.2 (1.2-1.2) | 0.280 |
| Creatinine, mg/dL | 0.9 (0.7-1.2) | 0.9 (0.8-1.1) | 0.305 | 0.9 (0.7-1.1) | 0.9 (0.7-1.2) | 0.503 |
| Prothrombin time, s | 1.1 (1.0-9.2) | 1.1 (1.0-9.3) | 0.108 | 1.1 (1.0-9.9) | 1.1 (1.0-8.8) | 0.397 |
| Platelet count, K/uL | 221.0 (166.0-311.0) | 200.0 (144.0-289.0) | 0.045 | 226.5 (160.5-317.8) | 195.0 (154.5-271.5) | 0.033 |

Data are expressed as median (interquartile range), n (%), or mean (standard deviation). AFP = alpha-fetoprotein.

**Table S3. Univariate and multivariate Cox proportional hazards regression analysis of cirrhotic HCC.**

|  | **Univariable** | | **Multivariable*** | | |
| --- | --- | --- | --- | --- | --- |
|  | **HR (95% CI)** | ***P* value** | **HR (95% CI)** | ***P* value** | **VIF^†^** |
| Age | 1.01 (0.99-1.03) | 0.070 |  |  |  |
| Male | 0.82 (0.58-1.17) | 0.276 |  |  |  |
| Height | 0.99 (0.98-1.02) | 0.799 |  |  |  |
| Weight | 0.99 (0.98-1.00) | 0.238 |  |  |  |
| Race |  |  |  |  |  |
| White | 1 [Reference] | NA |  |  |  |
| African American | 1.20 (0.52-2.77) | 0.666 |  |  |  |
| American Indian /Alaska native | 0.00 (0.00-Inf) | 0.995 |  |  |  |
| Asian | 0.76 (0.52-1.10) | 0.148 |  |  |  |
| Others | 1.57 (0.63-3.89) | 0.332 |  |  |  |
| Pathologic stage |  |  |  |  | 1.110 |
| Stage Ⅰ | 1 [Reference] | NA | 1 [Reference] | NA |  |
| Stage Ⅱ | 1.16 (0.73-1.85) | 0.527 | 0.93 (0.58-1.50) | 0.766 |  |
| Stage Ⅲ | 2.22 (1.50-3.29) | < 0.001 | 1.59 (1.05-2.43) | 0.029 |  |
| Stage Ⅳ | 4.79 (1.49-15.35) | 0.008 | 4.80 (1.49-15.45) | 0.009 |  |
| Child–Pugh score |  |  |  |  |  |
| Class A | 1 [Reference] | NA |  |  |  |
| Class B | 1.11 (0.54-2.28) | 0.778 |  |  |  |
| Class C | 1.54 (0.21-11.07) | 0.668 |  |  |  |
| Neoplasm histologic grade |  |  |  |  |  |
| G1 | 1 [Reference] | NA |  |  |  |
| G2 | 1.06 (0.65-1.72) | 0.825 |  |  |  |
| G3 | 1.08 (0.65-1.81) | 0.765 |  |  |  |
| G4 | 1.35 (0.51-3.57) | 0.551 |  |  |  |
| Ishak fibrosis score |  |  |  |  |  |
| 0 - No fibrosis | 1 [Reference] | NA |  |  |  |
| 1, 2 - Portal fibrosis | 0.64 (0.32-1.27) | 0.204 |  |  |  |
| 3, 4 - Fibrous septa | 0.43 (0.19-0.99) | 0.046 |  |  |  |
| 5 - Nodular formation/ incomplete cirrhosis | 0.52 (0.13-2.12) | 0.362 |  |  |  |
| 6 - Established cirrhosis | 0.53 (0.32-0.88) | 0.014 |  |  |  |
| Vascular invasion |  |  |  |  |  |
| None | 1 [Reference] | NA |  |  |  |
| Micro | 0.92 (0.61-1.41) | 0.713 |  |  |  |
| Macro | 1.57 (0.76-3.24) | 0.223 |  |  |  |
| Residual tumor grade |  |  |  |  |  |
| R0 | 1 [Reference] | NA |  |  |  |
| R1 | 1.45 (0.71-2.97) | 0.312 |  |  |  |
| R2 | 10.64 (1.45-77.86) | 0.020 |  |  |  |
| Rx | 3.11 (1.64-5.88) | < 0.001 |  |  |  |
| Radiation therapy | 0.87 (0.28-2.73) | 0.808 |  |  |  |
| Neoadjuvant therapy | 0.00 (0.00-Inf) | 0.994 |  |  |  |
| Embolization therapy | 0.80 (0.43-1.48) | 0.470 |  |  |  |
| Other malignant tumors | 1.10 (0.62-1.95) | 0.753 |  |  |  |
| Family history of cancer | 1.22 (0.85-1.73) | 0.277 |  |  |  |
| Risk factors |  |  |  |  |  |
| Hepatitis B | 0.35 (0.21-0.58) | < 0.001 |  |  |  |
| Hepatitis C | 1.12 (0.67-1.87) | 0.664 |  |  |  |
| Alcohol consumption | 1.46 (0.96-2.20) | 0.075 |  |  |  |
| Smoking | 0.79 (0.37-1.71) | 0.553 |  |  |  |
| Hepatic cirrhosis | 2.02 (0.50-8.18) | 0.326 |  |  |  |
| Non-alcoholic fatty liver disease | 0.89 (0.33-2.40) | 0.812 |  |  |  |
| Hemochromatosis | 0.48 (0.07-3.43) | 0.463 |  |  |  |
| Hepatic encephalopathy | 0.00 (0.00-Inf) | 0.995 |  |  |  |
| Diabetes mellitus | 3.74 (0.52-26.93) | 0.190 |  |  |  |
| Autoimmune hepatitis | 0.00 (0.00-Inf) | 0.995 |  |  |  |
| Primary biliary cholangitis | 0.00 (0.00-Inf) | 1.000 |  |  |  |
| α-1 antitrypsin deficiency | 7.44 (1.03-54.00) | 0.047 |  |  |  |
| Sarcoidosis | 0.00 (0.00-Inf) | 0.995 |  |  |  |
| Worker at steel factory | 0.00 (0.00-Inf) | 0.994 |  |  |  |
| Biomarkers |  |  |  |  |  |
| AFP (> =25 vs. < 25) | 2.09 (1.44-3.04) | < 0.001 | 1.73 (1.18-2.55) | 0.005 | 1.097 |
| Albumin (> =35 vs. < 35) | 3.07 (2.10-4.52) | < 0.001 | 2.45 (1.61-3.72) | < 0.001 | 1.158 |
| Total bilirubin, mg/dL | 0.96 (0.84-1.10) | 0.556 |  |  |  |
| Creatinine, mg/dL | 1.00 (0.99-1.02) | 0.778 |  |  |  |
| Prothrombin time, s | 1.02 (0.98-1.06) | 0.362 |  |  |  |
| Platelet count, K/uL | 1.00 (1.00-1.00) | 0.679 |  |  |  |

HR (hazard ratio) refers to total mortality risk relative to reference. AFP = alpha-fetoprotein.

*C statistics = 0.664 (95% CI 0.613 - 0.715), *P* < 0.001 (Likelihood ratio test); *P* < 0.001 (Wald test); *P* < 0.001 (Score log-rank test).

^†^VIF (Variance Inflation Factor) assesses multi-collinearity of independent variables in regression model (VIF ≥ 10 suggests multicollinearity).

**Table S4. Primers for qRT-PCR analysis**

|  | **Sense** | **Anti-sense** |
| --- | --- | --- |
| LncRNA EGOT | AAGGCAGAGGTGGGTTTGG | TGTGAGTAGGGAGGTTAGGATGAA |
| LncRNA SERHL | TCTTGTTTTCTTGCGGTTCTATTT | ATGGGCACTGGAGGGTCTG |
| XYLT2 | AGGTGGTACGGGCAGTAAC | GCTCCCTGTATCTCCGTGT |
| ARNT | CTGCCAACCCCGAAATGACAT | CGCCGCTTAATAGCCCTCTG |
| CHST3 | CTCGGAGCAGTTCGAGAAGTG | CGCCAGTTTGTAGCCGAAGA |
| LPCAT1 | ACATCCCGATCTGGGGAACT | GGCCACTTTCCGTTGGACT |
| BCL2L1 | GAGCTGGTGGTTGACTTTCTC | TCCATCTCCGATTCAGTCCCT |
| SYK | CATGGAAAAATCTCTCGGGAAGA | GTCGATGCGATAGTGCAGCA |
| miR-32-5p | TATTGCACATTACTAAGTTGCA | |
| miR-193b-3p | AACTGGCCCTCAAAGTCCCGCT | |
| miR-1269a | CTGGACTGAGCCGTGCTACTGG | |

qRT-PCR = quantitative real-time polymerase chain reaction.

**Table S5. HCC-specific differentially expressed lncRNAs from GSE17967**

| **lncRNA** | **style** | **Fold Change** | ***p*-value** | **FDR** |
| --- | --- | --- | --- | --- |
| COX7A2P2 | up | 1.429749 | 0.039857 | 0.452642 |
| LOC105370914 | up | 1.723018 | 0.048138 | 0.452642 |
| ENST00000621900.1 | up | 1.692572 | 0.04059 | 0.452642 |
| NONHSAT001301.2 | up | 1.849061 | 0.00741 | 0.29373 |
| NONHSAT010763.2 | up | 1.676756 | 0.047549 | 0.452642 |
| NONHSAT023390.2 | up | 1.579341 | 0.014453 | 0.329895 |
| NONHSAT070712.2 | up | 1.95962 | 0.038621 | 0.452642 |
| NONHSAT076682.2 | up | 1.78181 | 0.022474 | 0.410666 |
| NONHSAT123438.2 | up | 1.880433 | 0.01689 | 0.372433 |
| NONHSAT129571.2 | up | 1.944414 | 0.020722 | 0.410666 |
| NONHSAT130696.2 | up | 1.384365 | 0.035991 | 0.452642 |
| NONHSAT135135.2 | up | 2.094441 | 0.002735 | 0.211453 |
| NONHSAT145215.2 | up | 1.894025 | 0.014987 | 0.329895 |
| NONHSAT165295.1 | up | 1.575119 | 0.030699 | 0.452642 |
| NONHSAT171169.1 | up | 1.823535 | 0.034806 | 0.452642 |
| NONHSAT171432.1 | up | 1.600521 | 0.008435 | 0.29373 |
| NONHSAT175076.1 | up | 1.809133 | 0.01035 | 0.329895 |
| NONHSAT193357.1 | up | 1.711486 | 0.0287035 | 0.431654 |
| NR_003945.1 | up | 1.373122 | 0.033204 | 0.452642 |
| NR_026891.1 | up | 1.597825 | 0.03531 | 0.452642 |
| NR_051972.1 | up | 1.477361 | 0.038301 | 0.452642 |
| NR_134476.1 | up | 1.667927 | 0.044817 | 0.452642 |
| ASMTL-AS1 | down | -1.990937 | 0.02263 | 0.29373 |
| BTF3P11 | down | -2.662763 | 0.005575 | 0.173336 |
| EGOT | down | -2.298693 | 0.003774 | 0.145085 |
| LOC105376196 | down | -1.636733 | 0.031305 | 0.29373 |
| MAPKAPK5-AS1 | down | -1.973787 | 0.015167 | 0.243912 |
| MLLT10P1 | down | -1.744897 | 0.039115 | 0.329895 |
| NACAP1 | down | -1.654506 | 0.002282 | 0.121232 |
| PKD1P6 | down | -1.691174 | 0.001499 | 0.097436 |
| PMCHL1 | down | -2.026707 | 0.011046 | 0.243912 |
| PYY2 | down | -1.805021 | 0.026673 | 0.29373 |
| SERHL | down | -2.001781 | 0.030939 | 0.29373 |
| TMEM92-AS1 | down | -1.936807 | 0.009716 | 0.211453 |
| TNXA | down | -1.586614333 | 0.00510767 | 0.173336 |
| ENST00000395400.4 | down | -1.756897 | 0.011834 | 0.243912 |
| ENST00000414544.1 | down | -2.004658 | 0.007155 | 0.211453 |
| ENST00000420452.5 | down | -1.82672 | 0.01424 | 0.243912 |
| ENST00000435356.2 | down | -1.707345 | 0.035687 | 0.29373 |
| ENST00000497872.3 | down | -1.636207 | 0.032755 | 0.29373 |
| ENST00000562082.1 | down | -1.897708 | 0.029371 | 0.29373 |
| NONHSAT002975.2 | down | -1.581362 | 0.01408 | 0.243912 |
| NONHSAT004151.2 | down | -2.031608 | 0.027346 | 0.29373 |
| NONHSAT039751.2 | down | -2.255892 | 0.008301 | 0.211453 |
| NONHSAT041143.2 | down | -1.762247 | 0.009314 | 0.211453 |
| NONHSAT047228.2 | down | -1.239697 | 0.047125 | 0.329895 |
| NONHSAT047922.2 | down | -1.946878 | 0.014301 | 0.243912 |
| NONHSAT054874.2 | down | -1.687206 | 0.003994 | 0.173336 |
| NONHSAT060287.2 | down | -2.49378 | 0.004874 | 0.173336 |
| NONHSAT083938.2 | down | -1.277853 | 0.049991 | 0.372433 |
| NONHSAT094826.2 | down | -1.632347 | 0.021598 | 0.29373 |
| NONHSAT119971.2 | down | -1.561112 | 0.04426 | 0.329895 |
| NONHSAT121535.2 | down | -1.928509 | 0.003112 | 0.145085 |
| NONHSAT126168.2 | down | -2.113188 | 0.039668 | 0.329895 |
| NONHSAT137305.2 | down | -1.666447 | 0.025431 | 0.29373 |
| NONHSAT145653.2 | down | -1.924705 | 0.019025 | 0.243912 |
| NONHSAT157153.1 | down | -1.774541 | 0.042035 | 0.329895 |
| NONHSAT185078.1 | down | -1.616797 | 0.022764 | 0.2525915 |
| NONHSAT192084.1 | down | -1.629373 | 0.020945 | 0.29373 |
| NONHSAT206981.1 | down | -1.680323 | 0.03582 | 0.29373 |
| NONHSAT212252.1 | down | -1.464886 | 0.035103 | 0.29373 |
| NONHSAT220262.1 | down | -1.758844 | 0.034559 | 0.29373 |
| NONHSAT220650.1 | down | -1.901109 | 0.025014 | 0.29373 |
| NR_001284.2 | down | -2.320005 | 0.001117 | 0.087917 |
| NR_001554.2 | down | -2.09489 | 0.004604 | 0.173336 |
| NR_001559.2 | down | -2.010268 | 0.022618 | 0.29373 |
| NR_002174.2 | down | -1.395484 | 0.018905 | 0.243912 |
| NR_027259.1 | down | -2.36088 | 0.01169 | 0.243912 |
| NR_027434.2 | down | -1.909868 | 0.035234 | 0.29373 |
| NR_037932.1 | down | -1.257764 | 0.041134 | 0.329895 |
| NR_135506.1 | down | -1.267772 | 0.0459 | 0.329895 |
| XR_250850.2 | down | -2.46187 | 0.001006 | 0.087917 |
| XR_918449.1 | down | -1.336758 | 0.039347 | 0.329895 |
| XR_934561.1 | down | -1.870827 | 0.002002 | 0.121232 |

**Table S6. HCC-specific differentially expressed mRNAs from GSE17967**

| **Gene Symbol** | **Gene ID** | **style** | **Fold Change** | ***p*-value** | **FDR** |
| --- | --- | --- | --- | --- | --- |
| AAK1 | 22848 | up | 1.702046 | 0.044746 | 0.452642 |
| ABHD2 | 11057 | up | 1.646796 | 0.012191 | 0.329895 |
| ACSL6 | 23305 | up | 2.082483 | 0.00905 | 0.29373 |
| ACSS3 | 79611 | up | 1.542154 | 0.000825 | 0.145085 |
| ACTN3 | 89 | up | 1.597912 | 0.044737 | 0.452642 |
| ACVR2B | 93 | up | 1.576859 | 0.019138 | 0.372433 |
| ADAMTS13 | 11093 | up | 1.771245 | 0.012231 | 0.329895 |
| ADCY2 | 108 | up | 2.029898 | 0.013313 | 0.329895 |
| ADH1C | 126 | up | 1.640631 | 0.009701 | 0.29373 |
| ADH6 | 130 | up | 1.636083 | 0.007411 | 0.29373 |
| ADK | 132 | up | 1.661471 | 0.001171 | 0.173336 |
| AFM | 173 | up | 1.572879 | 0.004348 | 0.243912 |
| AK4 | 205 | up | 1.580709 | 0.001994 | 0.173336 |
| ALDH3A2 | 224 | up | 1.621318 | 0.001358 | 0.173336 |
| ANKRD7 | 56311 | up | 2.062879 | 0.035979 | 0.452642 |
| AP3D1 | 8943 | up | 1.736651 | 0.037373 | 0.452642 |
| APC | 324 | up | 1.950943 | 0.014152 | 0.329895 |
| AQP4 | 361 | up | 1.864974 | 0.018837 | 0.372433 |
| ARHGAP11A | 9824 | up | 1.948963 | 0.034926 | 0.452642 |
| ARHGEF12 | 23365 | up | 1.824605 | 0.048372 | 0.452642 |
| ASPH | 444 | up | 1.503473 | 0.008903 | 0.29373 |
| ASPSCR1 | 79058 | up | 1.810924 | 0.036002 | 0.452642 |
| ATAD2 | 29028 | up | 1.624771 | 0.004011 | 0.243912 |
| ATP13A3 | 79572 | up | 2.0324 | 0.003243 | 0.211453 |
| ATRX | 546 | up | 1.5576445 | 0.017165 | 0.352198 |
| B4GALT1 | 2683 | up | 1.7525655 | 0.028983 | 0.431654 |
| B9D1 | 27077 | up | 1.526088 | 0.03346 | 0.452642 |
| BAG5 | 9529 | up | 1.552098 | 0.002304 | 0.211453 |
| BCLAF1 | 9774 | up | 1.506447 | 0.010771 | 0.329895 |
| BTN1A1 | 696 | up | 2.143725 | 0.008561 | 0.29373 |
| CA14 | 23632 | up | 1.770995 | 0.038765 | 0.452642 |
| CARD14 | 79092 | up | 1.593444 | 0.043569 | 0.452642 |
| CASP10 | 843 | up | 1.8364935 | 0.012551 | 0.3330815 |
| CASP2 | 835 | up | 2.466752 | 0.001685 | 0.173336 |
| CAV3 | 859 | up | 1.9273 | 0.01597 | 0.329895 |
| CCDC177 | 56936 | up | 1.952898 | 0.021554 | 0.410666 |
| CCDC186 | 55088 | up | 1.556731 | 0.023566 | 0.410666 |
| CCDC88A | 55704 | up | 1.671075 | 0.036451 | 0.452642 |
| CCND1 | 595 | up | 1.523262 | 0.020139 | 0.372433 |
| CD164 | 8763 | up | 1.595781 | 0.000791 | 0.145085 |
| CD3G | 917 | up | 1.622968 | 0.042327 | 0.452642 |
| CD84 | 8832 | up | 1.802435 | 0.028945 | 0.452642 |
| CDC42EP2 | 10435 | up | 1.731821 | 0.034159 | 0.452642 |
| CDC6 | 990 | up | 1.684715 | 0.019847 | 0.372433 |
| CDCA4 | 55038 | up | 1.527119 | 0.043184 | 0.452642 |
| CDK6 | 1021 | up | 1.895829 | 0.022083 | 0.410666 |
| CEACAM3 | 1084 | up | 1.627756 | 0.04029 | 0.452642 |
| CELA3A | 10136 | up | 2.057341 | 0.021397 | 0.410666 |
| CENPA | 1058 | up | 2.153055 | 0.004042 | 0.243912 |
| CENPN | 55839 | up | 2.094924 | 0.00305 | 0.211453 |
| CHD4 | 1108 | up | 2.127432 | 0.004841 | 0.243912 |
| CHI3L2 | 1117 | up | 1.598959 | 0.043821 | 0.452642 |
| CHRM4 | 1132 | up | 2.062572 | 0.03522 | 0.452642 |
| CHRNA3 | 1136 | up | 1.8058445 | 0.0248 | 0.3912685 |
| CINP | 51550 | up | 1.548272 | 0.032604 | 0.452642 |
| CKS2 | 1164 | up | 1.624275 | 0.001036 | 0.145085 |
| CLN8 | 2055 | up | 2.427665 | 0.003794 | 0.243912 |
| COA7 | 65260 | up | 1.761633 | 0.009146 | 0.29373 |
| COBLL1 | 22837 | up | 1.744606 | 0.019547 | 0.372433 |
| CPA4 | 51200 | up | 1.557646 | 0.042206 | 0.452642 |
| CPT1A | 1374 | up | 1.642035 | 0.02598 | 0.410666 |
| CRAT | 1384 | up | 1.517347 | 0.002933 | 0.211453 |
| CRB1 | 23418 | up | 2.160692 | 0.010182 | 0.29373 |
| CREB3L1 | 90993 | up | 1.762868 | 0.015751 | 0.329895 |
| CYP11A1 | 1583 | up | 1.77649 | 0.049601 | 0.478182 |
| CYP1B1 | 1545 | up | 2.052515 | 0.022227 | 0.410666 |
| CYP26A1 | 1592 | up | 2.150211 | 0.027894 | 0.410666 |
| CYP3A4 | 1576 | up | 1.701688 | 0.040328 | 0.452642 |
| DAPK1 | 1612 | up | 1.794861 | 0.024823 | 0.410666 |
| DAZ1 | 1617 | up | 1.845787 | 0.031094 | 0.452642 |
| DBF4B | 80174 | up | 1.649169 | 0.042437 | 0.452642 |
| DBT | 1629 | up | 1.813299 | 0.003542 | 0.211453 |
| DCAF13 | 25879 | up | 1.809241 | 0.043049 | 0.452642 |
| DCAF17 | 80067 | up | 2.25498 | 0.003173 | 0.211453 |
| DDX17 | 10521 | up | 1.780363 | 0.034617 | 0.434419 |
| DEPDC1 | 55635 | up | 1.888883 | 0.029519 | 0.452642 |
| DGCR2 | 9993 | up | 1.934682 | 0.005946 | 0.243912 |
| DIO1 | 1733 | up | 1.567959 | 0.009909 | 0.29373 |
| DIO2 | 1734 | up | 1.610962 | 0.04287 | 0.452642 |
| DNAJA4 | 55466 | up | 1.625594 | 0.016286 | 0.372433 |
| DNMT3A | 1788 | up | 1.855778 | 0.032069 | 0.452642 |
| DPP4 | 1803 | up | 2.100487333 | 0.000471 | 0.0951783 |
| DTL | 51514 | up | 1.521589 | 0.030565 | 0.452642 |
| DTNA | 1837 | up | 1.650911 | 0.025413 | 0.4125375 |
| DYRK1A | 1859 | up | 1.828877 | 0.016231 | 0.372433 |
| EDEM3 | 80267 | up | 1.527603 | 0.002191 | 0.173336 |
| EEA1 | 8411 | up | 1.63789 | 0.032596 | 0.452642 |
| EED | 8726 | up | 1.96215 | 0.030187 | 0.452642 |
| EHHADH | 1962 | up | 1.614437 | 0.005111 | 0.243912 |
| EIF2S3 | 1968 | up | 1.762682 | 0.00808 | 0.29373 |
| EIF4G1 | 1981 | up | 1.526458 | 0.005448 | 0.243912 |
| EIF5A2 | 56648 | up | 2.006953 | 0.010247 | 0.29373 |
| ELOVL2 | 54898 | up | 1.79069 | 0.004914 | 0.243912 |
| EML2 | 24139 | up | 1.902167 | 0.017994 | 0.372433 |
| ENC1 | 8507 | up | 1.926482 | 0.015936 | 0.329895 |
| ENPP1 | 5167 | up | 1.543708 | 0.005771 | 0.243912 |
| EPB41L5 | 57669 | up | 1.65758 | 0.049972 | 0.478182 |
| EPRS | 2058 | up | 1.741093 | 0.039902 | 0.452642 |
| ERBB4 | 2066 | up | 2.11879 | 0.025589 | 0.410666 |
| ERCC4 | 2072 | up | 2.05041 | 0.011943 | 0.329895 |
| ESPL1 | 9700 | up | 1.882248 | 0.017093 | 0.372433 |
| ETV6 | 2120 | up | 1.749256 | 0.025034 | 0.410666 |
| EYA4 | 2070 | up | 1.947269 | 0.049095 | 0.452642 |
| F13B | 2165 | up | 1.52771 | 0.005893 | 0.243912 |
| F5 | 2153 | up | 1.673634 | 0.001635 | 0.173336 |
| FAM169A | 26049 | up | 1.928812 | 0.001891 | 0.173336 |
| FBXO4 | 26272 | up | 1.822594 | 0.046518 | 0.452642 |
| FLRT3 | 23767 | up | 1.50861 | 0.009342 | 0.29373 |
| FPR2 | 2358 | up | 1.924537 | 0.020176 | 0.372433 |
| FRK | 2444 | up | 1.61258 | 0.01522 | 0.329895 |
| G3BP1 | 10146 | up | 1.59025 | 0.001879 | 0.173336 |
| GABBR2 | 9568 | up | 2.015418 | 0.036152 | 0.452642 |
| GABRA4 | 2557 | up | 1.728885 | 0.045567 | 0.452642 |
| GABRD | 2563 | up | 2.022277 | 0.009061 | 0.29373 |
| GBA3 | 57733 | up | 1.524775 | 0.009409 | 0.29373 |
| GDF9 | 2661 | up | 1.838208 | 0.024845 | 0.410666 |
| GINS4 | 84296 | up | 1.755287 | 0.040906 | 0.452642 |
| GLYAT | 10249 | up | 1.831952 | 0.005975 | 0.243912 |
| GP2 | 2813 | up | 1.726607 | 0.04587 | 0.452642 |
| GPR75 | 10936 | up | 1.848061 | 0.029816 | 0.452642 |
| GRM8 | 2918 | up | 1.880721 | 0.040797 | 0.452642 |
| HACD3 | 51495 | up | 1.585432 | 0.000567 | 0.145085 |
| HELLS | 3070 | up | 2.189814 | 0.009352 | 0.29373 |
| HFE | 3077 | up | 1.861493333 | 0.02179 | 0.3467007 |
| HGC6.3 | 1E+08 | up | 1.741359 | 0.046511 | 0.452642 |
| HIST1H1D | 3007 | up | 1.534504 | 0.044978 | 0.452642 |
| HIST1H2BO | 8348 | up | 1.855095 | 0.036859 | 0.452642 |
| HLF | 3131 | up | 1.548483 | 0.001723 | 0.173336 |
| HMMR | 3161 | up | 2.023307 | 0.009262 | 0.29373 |
| HOXA11 | 3207 | up | 1.689357 | 0.029429 | 0.452642 |
| IFIT2 | 3433 | up | 1.672859 | 0.016019 | 0.372433 |
| IKZF3 | 22806 | up | 2.223105 | 0.004463 | 0.243912 |
| IKZF5 | 64376 | up | 1.589351 | 0.035662 | 0.452642 |
| IL13RA2 | 3598 | up | 2.705572 | 0.007526 | 0.29373 |
| IL1R2 | 7850 | up | 1.517745 | 0.038517 | 0.452642 |
| IL6ST | 3572 | up | 1.752285 | 0.001137 | 0.1592105 |
| ILF3 | 3609 | up | 1.731672 | 0.042017 | 0.452642 |
| ITPR2 | 3709 | up | 2.478879 | 0.001931 | 0.173336 |
| KCNJ9 | 3765 | up | 2.335611 | 0.002168 | 0.173336 |
| KDELR3 | 11015 | up | 1.749115 | 0.031561 | 0.452642 |
| KIR2DL3 | 3804 | up | 2.329475 | 0.003353 | 0.211453 |
| KLF12 | 11278 | up | 1.639224 | 0.028439 | 0.452642 |
| KLHL28 | 54813 | up | 1.759202 | 0.049338 | 0.478182 |
| KLK1 | 3816 | up | 1.63109 | 0.04223 | 0.452642 |
| KRAS | 3845 | up | 1.583646 | 0.000596 | 0.145085 |
| LGR5 | 8549 | up | 2.207571 | 0.027547 | 0.410666 |
| LHX6 | 26468 | up | 1.567225 | 0.021633 | 0.410666 |
| LMTK2 | 22853 | up | 1.703516 | 0.020757 | 0.410666 |
| LSM14B | 149986 | up | 1.655036 | 0.041753 | 0.452642 |
| LYPLA2 | 11313 | up | 1.552616 | 0.006167 | 0.243912 |
| MAGIX | 79917 | up | 1.671762 | 0.049414 | 0.478182 |
| MAP3K1 | 4214 | up | 1.706275 | 0.030169 | 0.452642 |
| MARCH6 | 10299 | up | 1.714754 | 0.042651 | 0.452642 |
| MARCKS | 4082 | up | 1.592922 | 0.04224 | 0.452642 |
| MEGF9 | 1955 | up | 1.574386 | 0.013249 | 0.329895 |
| MEP1B | 4225 | up | 2.297959 | 0.005329 | 0.243912 |
| MFAP3 | 4238 | up | 1.823988 | 0.027693 | 0.410666 |
| MOB1A | 55233 | up | 1.50438 | 0.015977 | 0.329895 |
| MPZL2 | 10205 | up | 2.190291 | 0.002549 | 0.211453 |
| MRPL19 | 9801 | up | 1.506169 | 0.001175 | 0.173336 |
| MSMO1 | 6307 | up | 1.504551 | 0.0033 | 0.211453 |
| MT3 | 4504 | up | 1.91095 | 0.032852 | 0.452642 |
| MTAP | 4507 | up | 1.717559 | 0.047823 | 0.452642 |
| MYO1B | 4430 | up | 1.549172 | 0.045747 | 0.452642 |
| NAT8B | 51471 | up | 1.681141 | 0.043222 | 0.452642 |
| NEU3 | 10825 | up | 1.763312 | 0.012614 | 0.329895 |
| NGLY1 | 55768 | up | 1.982798 | 0.012814 | 0.329895 |
| NPY2R | 4887 | up | 1.804086 | 0.048779 | 0.452642 |
| NRIP1 | 8204 | up | 1.511072 | 0.004401 | 0.243912 |
| NTRK2 | 4915 | up | 1.746038 | 0.020741 | 0.410666 |
| NUCKS1 | 64710 | up | 1.588932 | 0.006981 | 0.29373 |
| NUP50 | 10762 | up | 1.609021 | 0.001767 | 0.173336 |
| NXPH3 | 11248 | up | 1.648286 | 0.02933 | 0.452642 |
| OASL | 8638 | up | 1.5346425 | 0.038243 | 0.452642 |
| OTUB2 | 78990 | up | 1.905047 | 0.013249 | 0.329895 |
| OTUD4 | 54726 | up | 1.584763 | 0.04472 | 0.452642 |
| OXR1 | 55074 | up | 1.509054 | 0.002231 | 0.173336 |
| PACRG | 135138 | up | 1.930481 | 0.010044 | 0.29373 |
| PANK3 | 79646 | up | 1.82577 | 0.013736 | 0.329895 |
| PEG10 | 23089 | up | 2.532378 | 0.006647 | 0.2010115 |
| PGC | 5225 | up | 1.659208 | 0.031469 | 0.452642 |
| PGRMC1 | 10857 | up | 1.520156 | 0.011574 | 0.329895 |
| PHACTR1 | 221692 | up | 2.140501 | 0.009678 | 0.29373 |
| PI4K2A | 55361 | up | 1.542528 | 0.043574 | 0.452642 |
| PIK3C3 | 5289 | up | 1.742316 | 0.02614 | 0.410666 |
| PIK3CG | 5294 | up | 1.767708 | 0.025653 | 0.410666 |
| POLE2 | 5427 | up | 1.800442 | 0.005109 | 0.243912 |
| POLQ | 10721 | up | 2.212766 | 0.01335 | 0.329895 |
| PPFIA1 | 8500 | up | 1.607569 | 0.019105 | 0.372433 |
| PRAMEF1 | 65121 | up | 1.629903 | 0.039767 | 0.452642 |
| PRKAA2 | 5563 | up | 1.670431 | 0.026644 | 0.410666 |
| PRRC1 | 133619 | up | 1.531991 | 0.004426 | 0.243912 |
| PSEN2 | 5664 | up | 1.529913 | 0.003946 | 0.243912 |
| PSME4 | 23198 | up | 1.89098 | 0.000084 | 0.040071 |
| PTPN11 | 5781 | up | 1.53388 | 0.004168 | 0.2276825 |
| R3HDM1 | 23518 | up | 1.739474 | 0.021257 | 0.410666 |
| RABGAP1L | 9910 | up | 1.579423 | 0.024645 | 0.410666 |
| RAD23B | 5887 | up | 1.662054 | 0.008052 | 0.29373 |
| RASAL2 | 9462 | up | 1.681853 | 0.031752 | 0.452642 |
| RCAN1 | 1827 | up | 1.8515 | 0.028984 | 0.452642 |
| RFX7 | 64864 | up | 1.90468 | 0.005669 | 0.243912 |
| RHOBTB2 | 23221 | up | 1.853712 | 0.027277 | 0.410666 |
| RIOK3 | 8780 | up | 1.518895 | 0.003703 | 0.243912 |
| RNASEL | 6041 | up | 1.559629 | 0.024939 | 0.410666 |
| RORA | 6095 | up | 1.566671 | 0.011036 | 0.329895 |
| RTEL1 | 51750 | up | 1.768233 | 0.016782 | 0.372433 |
| SCAMP1 | 9522 | up | 1.5636425 | 0.016969 | 0.348277 |
| SCD | 6319 | up | 1.518955 | 0.041371 | 0.452642 |
| SCGB1D1 | 10648 | up | 1.928329 | 0.018728 | 0.372433 |
| SCLY | 51540 | up | 1.890933 | 0.021447 | 0.410666 |
| SCN5A | 6331 | up | 1.634994 | 0.032323 | 0.452642 |
| SCYL2 | 55681 | up | 1.663869 | 0.000818 | 0.145085 |
| SEMA6D | 80031 | up | 1.721098 | 0.040835 | 0.452642 |
| SIKE1 | 80143 | up | 1.730806 | 0.003895 | 0.243912 |
| SIRT5 | 23408 | up | 1.619932 | 0.04198 | 0.452642 |
| SLC16A1 | 6566 | up | 1.515019 | 0.030526 | 0.452642 |
| SLC22A17 | 51310 | up | 1.62477 | 0.035961 | 0.452642 |
| SLC24A1 | 9187 | up | 2.096117 | 0.008167 | 0.29373 |
| SLC28A3 | 64078 | up | 1.755566 | 0.018471 | 0.372433 |
| SLC2A5 | 6518 | up | 1.557326 | 0.046401 | 0.452642 |
| SLC35A3 | 23443 | up | 1.512025 | 0.000735 | 0.145085 |
| SLC45A2 | 51151 | up | 1.873734 | 0.033588 | 0.452642 |
| SLC4A4 | 8671 | up | 1.631785 | 0.024212 | 0.410666 |
| SLC7A8 | 23428 | up | 1.721722 | 0.009436 | 0.29373 |
| SLCO1B3 | 28234 | up | 2.163173 | 0.00102 | 0.145085 |
| SMAD5 | 4090 | up | 1.718426 | 0.007788 | 0.2869035 |
| SMC4 | 10051 | up | 1.777795 | 0.004354 | 0.243912 |
| SMG1 | 23049 | up | 1.673497 | 0.02779 | 0.410666 |
| SOCS1 | 8651 | up | 1.638268 | 0.021319 | 0.410666 |
| SORT1 | 6272 | up | 2.609371 | 0.001096 | 0.145085 |
| SOS2 | 6655 | up | 1.793995 | 0.014702 | 0.329895 |
| SPC25 | 57405 | up | 2.068315 | 0.020272 | 0.372433 |
| SPEN | 23013 | up | 1.517852 | 0.020958 | 0.410666 |
| SPTAN1 | 6709 | up | 1.616662 | 0.036488 | 0.452642 |
| SRPK3 | 26576 | up | 1.779849 | 0.017789 | 0.372433 |
| SSX2IP | 117178 | up | 1.56625925 | 0.007697 | 0.282202 |
| ST3GAL6 | 10402 | up | 1.621484 | 0.004718 | 0.243912 |
| STAT2 | 6773 | up | 1.607286 | 0.012915 | 0.329895 |
| STK24 | 8428 | up | 1.981639 | 0.020631 | 0.410666 |
| STRN | 6801 | up | 1.802254 | 0.032106 | 0.452642 |
| TAP2 | 6891 | up | 1.882586 | 0.018026 | 0.372433 |
| TAPT1 | 202018 | up | 2.181148 | 0.018475 | 0.372433 |
| TBL1Y | 90665 | up | 1.811562 | 0.026067 | 0.410666 |
| TCP10 | 6953 | up | 1.839037 | 0.017859 | 0.372433 |
| TEAD1 | 7003 | up | 1.607676 | 0.019456 | 0.372433 |
| TFR2 | 7036 | up | 1.693313 | 0.03126 | 0.452642 |
| TGFBR1 | 7046 | up | 2.179421 | 0.016046 | 0.372433 |
| THAP9 | 79725 | up | 1.792514 | 0.046663 | 0.452642 |
| TLE1 | 7088 | up | 1.646919 | 0.027192 | 0.410666 |
| TMEM106B | 54664 | up | 1.572317 | 0.000499 | 0.145085 |
| TMEM70 | 54968 | up | 1.610456 | 0.038445 | 0.452642 |
| TMPO | 7112 | up | 1.500537 | 0.00538 | 0.243912 |
| TNFRSF11A | 8792 | up | 1.816062 | 0.040276 | 0.452642 |
| TNFSF8 | 944 | up | 1.919247 | 0.00965 | 0.29373 |
| TOP1 | 7150 | up | 1.517489 | 0.009821 | 0.29373 |
| TOP2A | 7153 | up | 2.349853 | 0.003399 | 0.211453 |
| TRA2A | 29896 | up | 1.614634 | 0.039553 | 0.452642 |
| TRIM31 | 11074 | up | 1.681177 | 0.046791 | 0.452642 |
| TSFM | 10102 | up | 1.547231 | 0.021794 | 0.3915495 |
| TSR3 | 115939 | up | 2.163092 | 0.019582 | 0.372433 |
| TUBAL3 | 79861 | up | 2.426701 | 0.007836 | 0.29373 |
| UBE2W | 55284 | up | 1.544807 | 0.001465 | 0.173336 |
| UFL1 | 23376 | up | 1.527684 | 0.004621 | 0.243912 |
| UGT2A3 | 79799 | up | 1.711117 | 0.000387 | 0.145085 |
| UGT2B15 | 7366 | up | 1.51693 | 0.026654 | 0.410666 |
| UGT2B28 | 54490 | up | 1.597681 | 0.001278 | 0.173336 |
| USP12 | 219333 | up | 1.730735 | 0.04092 | 0.452642 |
| USP53 | 54532 | up | 1.727214 | 0.017067 | 0.372433 |
| UTY | 7404 | up | 1.613319 | 0.015129 | 0.329895 |
| VCPIP1 | 80124 | up | 1.710323 | 0.040613 | 0.452642 |
| WDR18 | 57418 | up | 2.20953 | 0.007865 | 0.29373 |
| WDR59 | 79726 | up | 1.929511 | 0.024034 | 0.410666 |
| WNK1 | 65125 | up | 1.571357 | 0.023774 | 0.410666 |
| XAF1 | 54739 | up | 1.749413 | 0.001776 | 0.173336 |
| YOD1 | 55432 | up | 1.70041 | 0.028156 | 0.410666 |
| ZBTB33 | 10009 | up | 1.777393 | 0.027504 | 0.410666 |
| ZBTB43 | 23099 | up | 1.791594 | 0.004825 | 0.243912 |
| ZEB1 | 6935 | up | 1.593121 | 0.002309 | 0.211453 |
| ZFY | 7544 | up | 1.774366 | 0.044816 | 0.452642 |
| ZG16 | 653808 | up | 1.903818 | 0.038004 | 0.452642 |
| ZKSCAN5 | 23660 | up | 1.671082 | 0.035794 | 0.452642 |
| ZMYM6 | 9204 | up | 1.839447 | 0.00571 | 0.243912 |
| ZMYND10 | 51364 | up | 1.876197 | 0.0435 | 0.452642 |
| ZNF124 | 7678 | up | 1.500846 | 0.014941 | 0.329895 |
| ZNF3 | 7551 | up | 2.41652 | 0.003647 | 0.211453 |
| ZNF345 | 25850 | up | 1.746578 | 0.03472 | 0.452642 |
| ZNF536 | 9745 | up | 2.129822 | 0.009206 | 0.29373 |
| ZNF674 | 641339 | up | 2.186602 | 0.036667 | 0.452642 |
| ZNF721 | 170960 | up | 1.570847 | 0.014955 | 0.329895 |
| ZWILCH | 55055 | up | 1.645275 | 0.033557 | 0.452642 |
| ZXDA | 7789 | up | 1.667847 | 0.023279 | 0.410666 |
| ABCB8 | 11194 | down | -1.987261 | 0.007907 | 0.211453 |
| ABCC3 | 8714 | down | -1.845319 | 0.024458 | 0.29373 |
| ACAP1 | 9744 | down | -1.637746 | 0.048003 | 0.329895 |
| ACE | 1636 | down | -1.782202 | 0.040007 | 0.329895 |
| ADAMTS1 | 9510 | down | -1.536287 | 0.017012 | 0.243912 |
| ADAMTSL2 | 9719 | down | -2.192177 | 0.012183 | 0.243912 |
| ADCY1 | 107 | down | -1.628105 | 0.03934 | 0.329895 |
| ADCY8 | 114 | down | -1.809019 | 0.02655 | 0.29373 |
| ADCY9 | 115 | down | -2.199566 | 0.003548 | 0.145085 |
| ADGRE5 | 976 | down | -2.347858 | 0.000059 | 0 |
| ADGRG1 | 9289 | down | -1.686529 | 0.011397 | 0.243912 |
| ADPRH | 141 | down | -1.566932 | 0.049126 | 0.372433 |
| ADRA1B | 147 | down | -2.133681 | 0.003199 | 0.145085 |
| ADRA2A | 150 | down | -1.516624 | 0.012986 | 0.243912 |
| AFF3 | 3899 | down | -1.610174 | 0.03605 | 0.329895 |
| AGER | 177 | down | -1.684895 | 0.025683 | 0.29373 |
| AGRN | 375790 | down | -1.533614 | 0.004427 | 0.173336 |
| AHDC1 | 27245 | down | -2.676442 | 0.001729 | 0.097436 |
| AIF1 | 199 | down | -1.583078 | 0.039627 | 0.329895 |
| AKAP8L | 26993 | down | -1.543669 | 0.044246 | 0.329895 |
| ALB | 213 | down | -1.771695 | 0.016345 | 0.243912 |
| ALDH1A2 | 8854 | down | -2.11551 | 0.001783 | 0.097436 |
| ALG12 | 79087 | down | -1.809338 | 0.002011 | 0.121232 |
| ALOX5 | 240 | down | -1.536747 | 0.027797 | 0.29373 |
| ANAPC13 | 25847 | down | -1.891481 | 0.046861 | 0.329895 |
| ANGPTL2 | 23452 | down | -2.054679 | 0.001322 | 0.097436 |
| ANK1 | 286 | down | -1.81831 | 0.005606 | 0.173336 |
| ANK3 | 288 | down | -1.614484 | 0.00262 | 0.145085 |
| ANKMY1 | 51281 | down | -1.683635 | 0.046884 | 0.329895 |
| ANXA9 | 8416 | down | -1.824527 | 0.007761 | 0.211453 |
| AP1G2 | 8906 | down | -2.620614 | 0.000069 | 0 |
| APBB1 | 322 | down | -1.774056 | 0.011337 | 0.243912 |
| APOE | 348 | down | -1.7228415 | 0.021005 | 0.268821 |
| ARFRP1 | 10139 | down | -1.582174 | 0.01589 | 0.243912 |
| ARHGEF17 | 9828 | down | -1.761576 | 0.021246 | 0.29373 |
| ARHGEF40 | 55701 | down | -1.779605 | 0.000224 | 0.0495 |
| ARID5A | 10865 | down | -1.809118 | 0.018628 | 0.243912 |
| ARNT | 405 | down | -1.69797 | 0.028116 | 0.29373 |
| ARPC4 | 10093 | down | -2.029501 | 0.013416 | 0.243912 |
| ASAP3 | 55616 | down | -1.771633 | 0.021653 | 0.29373 |
| ASCL2 | 430 | down | -2.116036 | 0.002893 | 0.145085 |
| ASMT | 438 | down | -1.809581 | 0.033004 | 0.29373 |
| ATF5 | 22809 | down | -1.742391 | 0.034351 | 0.29373 |
| ATP2A3 | 489 | down | -2.007242 | 0.023491 | 0.29373 |
| ATP6V1B1 | 525 | down | -1.894318 | 0.02407 | 0.29373 |
| AVIL | 10677 | down | -2.059648 | 0.005183 | 0.173336 |
| AXIN1 | 8312 | down | -1.824524 | 0.016632 | 0.243912 |
| AXL | 558 | down | -1.663878 | 0.025142 | 0.29373 |
| B3GALT2 | 8707 | down | -2.0051385 | 0.037204 | 0.3118125 |
| B4GALT6 | 9331 | down | -1.911693 | 0.006323 | 0.173336 |
| BAZ2A | 11176 | down | -1.55925 | 0.019666 | 0.243912 |
| BCAM | 4059 | down | -1.967733 | 0.007852 | 0.211453 |
| BCL2 | 596 | down | -1.874249 | 0.047532 | 0.329895 |
| BCL2L1 | 598 | down | -1.553283 | 0.014536 | 0.233533 |
| BCL3 | 602 | down | -2.0976405 | 0.005275 | 0.1663425 |
| BCL7C | 9274 | down | -2.061889 | 0.000785 | 0.087917 |
| BEST1 | 7439 | down | -1.708032 | 0.047833 | 0.329895 |
| BGN | 633 | down | -1.618269 | 0.003166 | 0.135386 |
| BLMH | 642 | down | -2.645309 | 0.000083 | 0 |
| BMP1 | 649 | down | -1.713898 | 0.026425 | 0.29373 |
| BMP3 | 651 | down | -1.598839 | 0.034621 | 0.29373 |
| BMPR1B | 658 | down | -1.711043 | 0.033991 | 0.29373 |
| BRF2 | 55290 | down | -1.727567 | 0.038329 | 0.329895 |
| BTBD18 | 643376 | down | -1.57404 | 0.049507 | 0.372433 |
| BUB1 | 699 | down | -1.934422 | 0.01556 | 0.243912 |
| BYSL | 705 | down | -1.787432 | 0.033251 | 0.29373 |
| C14orf93 | 60686 | down | -2.07757 | 0.035928 | 0.329895 |
| C1QB | 713 | down | -1.711696 | 0.002645 | 0.145085 |
| C21orf2 | 755 | down | -1.761717 | 0.0427 | 0.329895 |
| C3orf36 | 80111 | down | -1.605162 | 0.031538 | 0.29373 |
| C9orf116 | 138162 | down | -2.160433 | 0.002408 | 0.121232 |
| C9orf16 | 79095 | down | -1.569437 | 0.027923 | 0.29373 |
| CA11 | 770 | down | -1.595013 | 0.010483 | 0.211453 |
| CABIN1 | 23523 | down | -1.568655 | 0.008135 | 0.211453 |
| CACNA1A | 773 | down | -2.13364 | 0.02977 | 0.29373 |
| CACNA1C | 775 | down | -3.118085 | 0.000058 | 0 |
| CACNA1G | 8913 | down | -2.518955 | 0.004609 | 0.173336 |
| CALM1 | 801 | down | -1.517421 | 0.030316 | 0.29373 |
| CALM2 | 805 | down | -2.073619 | 0.000508 | 0.072128 |
| CALR | 811 | down | -1.596088 | 0.00164 | 0.1086065 |
| CAMK2B | 816 | down | -1.6054 | 0.01966 | 0.243912 |
| CAPN10 | 11132 | down | -2.178284 | 0.001383 | 0.097436 |
| CAPN6 | 827 | down | -1.815298 | 0.03714 | 0.329895 |
| CARM1 | 10498 | down | -1.796441 | 0.000439 | 0.072128 |
| CASKIN2 | 57513 | down | -2.1607885 | 0.001887 | 0.0972925 |
| CAV2 | 858 | down | -1.902869 | 0.009565 | 0.211453 |
| CCDC181 | 57821 | down | -1.819532 | 0.010111 | 0.211453 |
| CCDC85B | 11007 | down | -1.603115 | 0.047287 | 0.329895 |
| CCL2 | 6347 | down | -2.474818 | 0.008577 | 0.211453 |
| CCL3 | 6348 | down | -1.753613 | 0.001314 | 0.097436 |
| CCL5 | 6352 | down | -1.568818 | 0.003569 | 0.145085 |
| CCL7 | 6354 | down | -1.714736 | 0.026885 | 0.29373 |
| CCNF | 899 | down | -1.715936 | 0.022526 | 0.29373 |
| CCT8L2 | 150160 | down | -2.100829 | 0.011054 | 0.243912 |
| CD226 | 10666 | down | -1.509724 | 0.023651 | 0.29373 |
| CD300A | 11314 | down | -1.9716 | 0.028963 | 0.29373 |
| CD40 | 958 | down | -1.648777 | 0.038752 | 0.329895 |
| CDC42EP4 | 23580 | down | -1.85823 | 0.003183 | 0.145085 |
| CDH6 | 1004 | down | -1.66987 | 0.044565 | 0.329895 |
| CDHR5 | 53841 | down | -2.22933 | 0.004476 | 0.173336 |
| CDIP1 | 29965 | down | -1.662824 | 0.032716 | 0.29373 |
| CDK10 | 8558 | down | -2.279658 | 0.009625 | 0.211453 |
| CDKN2B | 1030 | down | -2.016321 | 0.016744 | 0.243912 |
| CDY1 | 9085 | down | -2.031184 | 0.025695 | 0.29373 |
| CDYL | 9425 | down | -1.77649 | 0.029864 | 0.29373 |
| CEL | 1056 | down | -2.041828 | 0.00409 | 0.173336 |
| CFB | 629 | down | -1.774136 | 0.044606 | 0.329895 |
| CH25H | 9023 | down | -1.681482 | 0.028332 | 0.29373 |
| CHD5 | 26038 | down | -1.971084 | 0.019491 | 0.243912 |
| CHRD | 8646 | down | -1.73886 | 0.00902 | 0.170674 |
| CHRNE | 1145 | down | -1.707879 | 0.02594 | 0.29373 |
| CHST15 | 51363 | down | -1.847351 | 0.049451 | 0.372433 |
| CHST3 | 9469 | down | -1.528296 | 0.005192 | 0.173336 |
| CLDN10 | 9071 | down | -1.673361 | 0.001375 | 0.097436 |
| CLEC11A | 6320 | down | -1.66646 | 0.027194 | 0.29373 |
| CLIP3 | 25999 | down | -1.723701 | 0.01413 | 0.243912 |
| CNOT3 | 4849 | down | -2.102959 | 0.020163 | 0.29373 |
| COL4A2 | 1284 | down | -1.510865 | 0.01343 | 0.243912 |
| COL6A1 | 1291 | down | -1.543648 | 0.041241 | 0.329895 |
| COL6A2 | 1292 | down | -1.524009 | 0.023692 | 0.29373 |
| COMMD9 | 29099 | down | -1.564964 | 0.001389 | 0.097436 |
| COX7A1 | 1346 | down | -1.516044 | 0.015061 | 0.243912 |
| CPSF1 | 29894 | down | -1.594813 | 0.010637 | 0.211453 |
| CREM | 1390 | down | -1.560509 | 0.047184 | 0.329895 |
| CRIP2 | 1397 | down | -1.716567 | 0.022473 | 0.29373 |
| CRLF1 | 9244 | down | -1.804072 | 0.024324 | 0.29373 |
| CRP | 1401 | down | -2.105648 | 0.022665 | 0.268821 |
| CRX | 1406 | down | -1.790518 | 0.021152 | 0.29373 |
| CRYGC | 1420 | down | -1.670489 | 0.049351 | 0.372433 |
| CSF3 | 1440 | down | -1.949309 | 0.007162 | 0.211453 |
| CSF3R | 1441 | down | -1.711768 | 0.001473 | 0.097436 |
| CTDSPL | 10217 | down | -2.180568 | 0.014366 | 0.1908235 |
| CTTN | 2017 | down | -1.875589 | 0.027056 | 0.29373 |
| CUL3 | 8452 | down | -1.721269 | 0.030246 | 0.29373 |
| CUL7 | 9820 | down | -2.160191 | 0.003057 | 0.145085 |
| CXCL1 | 2919 | down | -1.695421 | 0.022985 | 0.29373 |
| CXCL3 | 2921 | down | -1.530028 | 0.032911 | 0.29373 |
| CYBB | 1536 | down | -1.948888 | 0.02063 | 0.29373 |
| CYP11B2 | 1585 | down | -1.772221 | 0.005191 | 0.173336 |
| CYTL1 | 54360 | down | -1.750695 | 0.039496 | 0.329895 |
| DAPK2 | 23604 | down | -2.411627 | 0.000955 | 0.087917 |
| DCSTAMP | 81501 | down | -1.855669 | 0.047833 | 0.329895 |
| DDAH2 | 23564 | down | -1.640672 | 0.041031 | 0.329895 |
| DDX23 | 9416 | down | -2.27395 | 0.000356 | 0.072128 |
| DDX4 | 54514 | down | -1.678184 | 0.032324 | 0.29373 |
| DEDD | 9191 | down | -1.545304 | 0.015335 | 0.243912 |
| DENND3 | 22898 | down | -1.553601 | 0.028628 | 0.29373 |
| DEPDC5 | 9681 | down | -1.921371 | 0.015873 | 0.243912 |
| DHRS12 | 79758 | down | -2.30817 | 0.00114 | 0.087917 |
| DHX30 | 22907 | down | -1.504058 | 0.00187 | 0.121232 |
| DHX58 | 79132 | down | -1.968612 | 0.021908 | 0.29373 |
| DIAPH3 | 81624 | down | -2.048099 | 0.020782 | 0.29373 |
| DLG4 | 1742 | down | -1.97064 | 0.010734 | 0.211453 |
| DMPK | 1760 | down | -1.825444 | 0.049668 | 0.372433 |
| DMTN | 2039 | down | -1.978545 | 0.0001 | 0 |
| DMWD | 1762 | down | -1.872224 | 0.029261 | 0.29373 |
| DOHH | 83475 | down | -1.630682 | 0.049408 | 0.372433 |
| DPYSL4 | 10570 | down | -1.785457 | 0.031088 | 0.29373 |
| DRD4 | 1815 | down | -1.62752 | 0.014868 | 0.243912 |
| DRP2 | 1821 | down | -1.833531 | 0.015768 | 0.243912 |
| DUSP8 | 1850 | down | -1.847155 | 0.004155 | 0.173336 |
| DXO | 1797 | down | -1.954584 | 0.003492 | 0.145085 |
| DZIP3 | 9666 | down | -1.767464 | 0.03626 | 0.329895 |
| EDDM3B | 64184 | down | -1.877366 | 0.027617 | 0.29373 |
| EFEMP1 | 2202 | down | -1.505803 | 0.018905 | 0.243912 |
| EFNA5 | 1946 | down | -2.692037 | 0.000766 | 0.087917 |
| EGR4 | 1961 | down | -2.163571 | 0.01421 | 0.243912 |
| EHD1 | 10938 | down | -2.115097 | 0.010558 | 0.211453 |
| EHD2 | 30846 | down | -1.629593 | 0.023114 | 0.23749 |
| EHMT2 | 10919 | down | -2.354808 | 0.000607 | 0.072128 |
| ELANE | 1991 | down | -1.734327 | 0.045135 | 0.329895 |
| ELAVL3 | 1995 | down | -1.907482 | 0.031877 | 0.29373 |
| ELF4 | 2000 | down | -1.662222 | 0.041668 | 0.329895 |
| ENG | 2022 | down | -1.504839 | 0.023264 | 0.29373 |
| ENO2 | 2026 | down | -2.014344 | 0.018939 | 0.243912 |
| EPHB3 | 2049 | down | -1.685839 | 0.028474 | 0.29373 |
| EPN3 | 55040 | down | -2.074133 | 0.009403 | 0.211453 |
| EPOR | 2057 | down | -1.997311 | 0.006385 | 0.173336 |
| EPS8L1 | 54869 | down | -1.892723 | 0.008512 | 0.211453 |
| ERVMER34-1 | 1E+08 | down | -1.633963 | 0.028407 | 0.29373 |
| ESRRA | 2101 | down | -1.544964 | 0.0447 | 0.329895 |
| ETV4 | 2118 | down | -1.765058 | 0.024323 | 0.29373 |
| EXOC7 | 23265 | down | -1.775907 | 0.021604 | 0.29373 |
| FABP3 | 2170 | down | -1.789241 | 0.019484 | 0.243912 |
| FAM120C | 54954 | down | -1.65493 | 0.024715 | 0.29373 |
| FAM149B1 | 317662 | down | -1.962654 | 0.020663 | 0.29373 |
| FAM168B | 130074 | down | -1.527231 | 0.008021 | 0.211453 |
| FAM89B | 23625 | down | -1.547829 | 0.035539 | 0.29373 |
| FASN | 2194 | down | -2.107995 | 0.015974 | 0.243912 |
| FBXL15 | 79176 | down | -1.859431 | 0.0196 | 0.243912 |
| FBXO24 | 26261 | down | -1.590461 | 0.043117 | 0.329895 |
| FES | 2242 | down | -1.914443 | 0.000669 | 0.087917 |
| FGF23 | 8074 | down | -1.746023 | 0.037176 | 0.329895 |
| FGFR2 | 2263 | down | -1.500525 | 0.007368 | 0.211453 |
| FHL3 | 2275 | down | -1.756131 | 0.015473 | 0.243912 |
| FKBP8 | 23770 | down | -1.8289865 | 0.017368 | 0.233533 |
| FLOT2 | 2319 | down | -1.591485 | 0.004162 | 0.173336 |
| FMNL1 | 752 | down | -1.957435 | 0.002757 | 0.145085 |
| FNDC4 | 64838 | down | -1.691161 | 0.002257 | 0.121232 |
| FOLR3 | 2352 | down | -1.695268 | 0.032324 | 0.29373 |
| FOSB | 2354 | down | -2.510219 | 0.018806 | 0.243912 |
| FOXD4 | 2298 | down | -1.707839 | 0.012994 | 0.243912 |
| FOXE1 | 2304 | down | -1.749635 | 0.017014 | 0.243912 |
| FOXM1 | 2305 | down | -1.722356 | 0.02762 | 0.29373 |
| FOXO4 | 4303 | down | -1.964286 | 0.005861 | 0.173336 |
| FRRS1L | 23732 | down | -1.885128 | 0.037207 | 0.329895 |
| FSCN1 | 6624 | down | -1.756431 | 0.023942 | 0.29373 |
| FURIN | 5045 | down | -1.660975 | 0.000843 | 0.087917 |
| FUT6 | 2528 | down | -1.642208 | 0.045805 | 0.329895 |
| FXYD2 | 486 | down | -1.525854 | 0.004444 | 0.173336 |
| FZD1 | 8321 | down | -2.062839 | 0.002055 | 0.121232 |
| G6PD | 2539 | down | -1.798851 | 0.039243 | 0.329895 |
| GAD1 | 2571 | down | -1.590862 | 0.023788 | 0.29373 |
| GALK1 | 2584 | down | -2.326947 | 0.003777 | 0.145085 |
| GAS1 | 2619 | down | -1.585797 | 0.021497 | 0.268821 |
| GAS8 | 2622 | down | -1.711767 | 0.014097 | 0.243912 |
| GATA1 | 2623 | down | -1.597397 | 0.031335 | 0.29373 |
| GDF15 | 9518 | down | -1.809736 | 0.014537 | 0.243912 |
| GEM | 2669 | down | -1.548152 | 0.028583 | 0.29373 |
| GGT5 | 2687 | down | -3.257706 | 0.000077 | 0 |
| GH2 | 2689 | down | -1.628795 | 0.036114 | 0.329895 |
| GID4 | 79018 | down | -2.55797 | 0.001467 | 0.097436 |
| GKN1 | 56287 | down | -2.137401 | 0.006393 | 0.173336 |
| GNA11 | 2767 | down | -1.640024 | 0.00199 | 0.121232 |
| GNAS | 2778 | down | -1.841117 | 0.015913 | 0.243912 |
| GNB2 | 2783 | down | -1.533107 | 0.000623 | 0.072128 |
| GNGT1 | 2792 | down | -2.233224 | 0.010787 | 0.211453 |
| GPC4 | 2239 | down | -1.74723 | 0.002257 | 0.121232 |
| GPR135 | 64582 | down | -1.959681 | 0.009021 | 0.211453 |
| GPR161 | 23432 | down | -1.639341 | 0.042331 | 0.329895 |
| GPR31 | 2853 | down | -1.738905 | 0.012335 | 0.243912 |
| GPR4 | 2828 | down | -1.67344 | 0.041254 | 0.329895 |
| GPSM2 | 29899 | down | -1.928479 | 0.027891 | 0.29373 |
| GRIA2 | 2891 | down | -1.661472 | 0.037311 | 0.329895 |
| GRM1 | 2911 | down | -1.953897 | 0.03128 | 0.29373 |
| GUCY2C | 2984 | down | -1.969866 | 0.021837 | 0.29373 |
| GULP1 | 51454 | down | -2.099016 | 0.016826 | 0.243912 |
| HAND2 | 9464 | down | -2.324761 | 0.003385 | 0.145085 |
| HAP1 | 9001 | down | -1.889377 | 0.028389 | 0.29373 |
| HBZ | 3050 | down | -2.285662 | 0.013308 | 0.243912 |
| HCFC1 | 3054 | down | -1.728017 | 0.049326 | 0.372433 |
| HCFC1R1 | 54985 | down | -1.5128695 | 0.011058 | 0.208624 |
| HCRTR1 | 3061 | down | -1.617173 | 0.047702 | 0.329895 |
| HECTD3 | 79654 | down | -1.513644 | 0.00072 | 0.087917 |
| HERC2 | 8924 | down | -1.93554 | 0.016894 | 0.243912 |
| HIC1 | 3090 | down | -1.683677 | 0.047368 | 0.329895 |
| HIP1R | 9026 | down | -1.804561 | 0.029613 | 0.29373 |
| HK1 | 3098 | down | -1.53263 | 0.024376 | 0.29373 |
| HK3 | 3101 | down | -1.587253 | 0.044511 | 0.329895 |
| HLA-DPA1 | 3113 | down | -1.564235 | 0.008729 | 0.211453 |
| HRASLS2 | 54979 | down | -1.764593 | 0.009633 | 0.211453 |
| HRC | 3270 | down | -1.90553 | 0.005978 | 0.173336 |
| HSD17B3 | 3293 | down | -2.016156 | 0.010466 | 0.211453 |
| HSF1 | 3297 | down | -1.733026 | 0.03172 | 0.29373 |
| HSF4 | 3299 | down | -1.581673 | 0.033139 | 0.29373 |
| HSPB2 | 3316 | down | -1.758015 | 0.03757 | 0.329895 |
| HSPG2 | 3339 | down | -2.245346 | 0.004075 | 0.173336 |
| HTR5A | 3361 | down | -1.713421 | 0.046279 | 0.329895 |
| HTR7 | 3363 | down | -1.898337 | 0.016488 | 0.243912 |
| IDH3G | 3421 | down | -1.918346 | 0.010835 | 0.243912 |
| IFT122 | 55764 | down | -3.086743 | 0.000417 | 0.072128 |
| IGF2BP3 | 10643 | down | -2.356929 | 0.00801 | 0.211453 |
| IGH | 3492 | down | -2.203039 | 0.030091 | 0.29373 |
| IGSF9B | 22997 | down | -1.599131 | 0.026886 | 0.29373 |
| IL1RAPL1 | 11141 | down | -1.566633 | 0.010177 | 0.211453 |
| IL20RA | 53832 | down | -1.722884 | 0.020074 | 0.243912 |
| IL26 | 55801 | down | -2.03431 | 0.030272 | 0.29373 |
| INO80B | 83444 | down | -1.952079 | 0.023074 | 0.29373 |
| IP6K1 | 9807 | down | -1.741083 | 0.039865 | 0.329895 |
| IRAK1 | 3654 | down | -1.587452 | 0.020154 | 0.29373 |
| ITGA7 | 3679 | down | -2.027732 | 0.009198 | 0.211453 |
| ITGB1BP2 | 26548 | down | -1.805724 | 0.041956 | 0.329895 |
| ITPK1 | 3705 | down | -1.583039 | 0.045767 | 0.329895 |
| ITPR3 | 3710 | down | -2.038317 | 0.019113 | 0.243912 |
| JMJD4 | 65094 | down | -1.599938 | 0.049022 | 0.329895 |
| JMJD6 | 23210 | down | -1.756165 | 0.036653 | 0.329895 |
| JRK | 8629 | down | -1.85571 | 0.019441 | 0.243912 |
| JUNB | 3726 | down | -1.797358 | 0.000595 | 0.072128 |
| KAZN | 23254 | down | -1.766516 | 0.043924 | 0.329895 |
| KCNA2 | 3737 | down | -2.242273 | 0.007458 | 0.211453 |
| KCNAB2 | 8514 | down | -1.854121 | 0.005768 | 0.173336 |
| KCNJ4 | 3761 | down | -1.906576 | 0.015799 | 0.243912 |
| KCNN3 | 3782 | down | -1.767457 | 0.039959 | 0.329895 |
| KDSR | 2531 | down | -1.89845 | 0.048228 | 0.329895 |
| KERA | 11081 | down | -2.19093 | 0.029103 | 0.29373 |
| KIF21B | 23046 | down | -1.621974 | 0.039225 | 0.329895 |
| KIFC3 | 3801 | down | -2.12249 | 0.014451 | 0.243912 |
| KIR2DL1 | 3802 | down | -1.755505 | 0.044134 | 0.329895 |
| KIR3DL1 | 3811 | down | -1.835512 | 0.007141 | 0.211453 |
| KLHL22 | 84861 | down | -1.687588 | 0.032651 | 0.29373 |
| KLK10 | 5655 | down | -1.838476 | 0.019583 | 0.243912 |
| KLK14 | 43847 | down | -1.763279 | 0.011622 | 0.243912 |
| KMT2B | 9757 | down | -1.977449 | 0.003726 | 0.145085 |
| KRT13 | 3860 | down | -1.902982 | 0.048864 | 0.329895 |
| KRTAP9-9 | 81870 | down | -2.353561 | 0.009257 | 0.211453 |
| L3MBTL1 | 26013 | down | -2.0720375 | 0.01088 | 0.2276825 |
| LAMP1 | 3916 | down | -1.60898 | 0.018117 | 0.243912 |
| LAT | 27040 | down | -1.607206 | 0.034892 | 0.29373 |
| LDB3 | 11155 | down | -1.76788 | 0.029761 | 0.29373 |
| LDHC | 3948 | down | -1.842006 | 0.044152 | 0.329895 |
| LENEP | 55891 | down | -1.606742 | 0.023308 | 0.29373 |
| LGALS7 | 3963 | down | -1.536179 | 0.044539 | 0.329895 |
| LHX5 | 64211 | down | -1.59083 | 0.023463 | 0.29373 |
| LILRA3 | 11026 | down | -1.557471 | 0.036516 | 0.329895 |
| LILRB1 | 10859 | down | -1.788171 | 0.031716 | 0.29373 |
| LILRB5 | 10990 | down | -1.606807 | 0.045148 | 0.329895 |
| LIME1 | 54923 | down | -1.501621 | 0.011866 | 0.243912 |
| LIMK1 | 3984 | down | -2.003084 | 0.007737 | 0.211453 |
| LMOD1 | 25802 | down | -1.991188 | 0.016449 | 0.243912 |
| LOC100506571 | 1.01E+08 | down | -1.510831 | 0.032505 | 0.29373 |
| LPCAT1 | 79888 | down | -2.782525 | 0.000451 | 0.072128 |
| LRP1 | 4035 | down | -1.614697 | 0.02109 | 0.29373 |
| LRP2BP | 55805 | down | -1.654602 | 0.046742 | 0.329895 |
| LRRC32 | 2615 | down | -1.751247 | 0.003505 | 0.145085 |
| LRRC75B | 388886 | down | -1.733137 | 0.027274 | 0.29373 |
| LSP1 | 4046 | down | -1.585957 | 0.010494 | 0.211453 |
| LST1 | 7940 | down | -1.5093 | 0.048095 | 0.329895 |
| LTB4R | 1241 | down | -1.924492 | 0.011108 | 0.243912 |
| LTB4R2 | 56413 | down | -2.125849 | 0.001364 | 0.097436 |
| LTBP4 | 8425 | down | -2.680191 | 0.000086 | 0 |
| LTC4S | 4056 | down | -2.168898 | 0.006202 | 0.173336 |
| LXN | 56925 | down | -1.773213 | 0.010002 | 0.211453 |
| LZTS1 | 11178 | down | -1.529407 | 0.014126 | 0.243912 |
| MAGEA11 | 4110 | down | -1.968477 | 0.033554 | 0.29373 |
| MAGEC3 | 139081 | down | -2.759994 | 0.000329 | 0.072128 |
| MAGI2 | 9863 | down | -1.862768 | 0.004514 | 0.173336 |
| MAN1A2 | 10905 | down | -1.938991 | 0.011359 | 0.243912 |
| MAP3K6 | 9064 | down | -1.894555 | 0.017036 | 0.243912 |
| MAP4 | 4134 | down | -1.529727 | 0.005436 | 0.173336 |
| MAP4K1 | 11184 | down | -2.0315695 | 0.008564 | 0.208624 |
| MAP4K2 | 5871 | down | -1.822911 | 0.014709 | 0.243912 |
| MAPK12 | 6300 | down | -1.732205 | 0.010328 | 0.211453 |
| MAPK14 | 1432 | down | -1.527283 | 0.007813 | 0.211453 |
| MAPK1IP1L | 93487 | down | -2.910882 | 0.001097 | 0.087917 |
| MAPK8IP2 | 23542 | down | -1.806688 | 0.003892 | 0.145085 |
| MAPK8IP3 | 23162 | down | -2.151252 | 0.001619 | 0.097436 |
| MAPRE3 | 22924 | down | -2.238437 | 0.003723 | 0.145085 |
| MARK2 | 2011 | down | -1.5527 | 0.040588 | 0.329895 |
| MAST4 | 375449 | down | -1.715408 | 0.019458 | 0.2525915 |
| MBD1 | 4152 | down | -1.779723 | 0.014937 | 0.243912 |
| MCF2L | 23263 | down | -1.816884 | 0.012098 | 0.243912 |
| MDC1 | 9656 | down | -2.208984 | 0.004352 | 0.173336 |
| MEF2C | 4208 | down | -1.500066 | 0.034826 | 0.29373 |
| MEX3D | 399664 | down | -1.620439 | 0.025869 | 0.29373 |
| MGAT3 | 4248 | down | -1.662217 | 0.003562 | 0.145085 |
| MICALL1 | 85377 | down | -1.636762 | 0.049354 | 0.372433 |
| MLLT1 | 4298 | down | -1.646691 | 0.033247 | 0.29373 |
| MLXIPL | 51085 | down | -2.121837 | 0.00099 | 0.087917 |
| MMP13 | 4322 | down | -1.945462 | 0.008052 | 0.211453 |
| MMP15 | 4324 | down | -2.941255 | 0.000105 | 0 |
| MPL | 4352 | down | -1.916868 | 0.043982 | 0.329895 |
| MPP6 | 51678 | down | -1.550547 | 0.049712 | 0.372433 |
| MPPED2 | 744 | down | -1.9253 | 0.003088 | 0.145085 |
| MRC2 | 9902 | down | -1.533106 | 0.041927 | 0.329895 |
| MRPS2 | 51116 | down | -1.607101 | 0.004625 | 0.173336 |
| MSC | 9242 | down | -1.728134 | 0.007445 | 0.211453 |
| MSH5 | 4439 | down | -2.21119 | 0.007747 | 0.211453 |
| MTMR11 | 10903 | down | -1.504983 | 0.026028 | 0.29373 |
| MTSS1L | 92154 | down | -1.546627 | 0.045538 | 0.329895 |
| MUC6 | 4588 | down | -1.671771 | 0.001877 | 0.121232 |
| MUC8 | 1E+08 | down | -1.955871 | 0.024398 | 0.29373 |
| MUM1 | 84939 | down | -1.51401 | 0.005263 | 0.173336 |
| MVK | 4598 | down | -1.587292 | 0.049336 | 0.372433 |
| MVP | 9961 | down | -1.704477 | 0.001709 | 0.097436 |
| MXD3 | 83463 | down | -1.568265 | 0.049607 | 0.372433 |
| MYB | 4602 | down | -1.722731 | 0.046168 | 0.329895 |
| MYH11 | 4629 | down | -1.885822 | 0.016548 | 0.243912 |
| MYO5A | 4644 | down | -1.623997 | 0.038784 | 0.329895 |
| MYO9B | 4650 | down | -1.708713 | 0.015506 | 0.243912 |
| MYOZ2 | 51778 | down | -1.740669 | 0.027129 | 0.29373 |
| MYT1L | 23040 | down | -2.369988 | 0.004266 | 0.173336 |
| MZF1 | 7593 | down | -1.707311 | 0.014865 | 0.243912 |
| N4BP1 | 9683 | down | -1.741064 | 0.048545 | 0.329895 |
| NAA60 | 79903 | down | -1.657884 | 0.00127 | 0.097436 |
| NAV3 | 89795 | down | -1.757734 | 0.032174 | 0.29373 |
| NCF1 | 653361 | down | -1.593967 | 0.048151 | 0.329895 |
| NCLN | 56926 | down | -1.650529 | 0.02691 | 0.29373 |
| NDOR1 | 27158 | down | -1.633714 | 0.037736 | 0.329895 |
| NDRG4 | 65009 | down | -2.806267 | 0.000142 | 0 |
| NDST1 | 3340 | down | -1.965093 | 0.016604 | 0.243912 |
| NEFL | 4747 | down | -1.781054 | 0.049061 | 0.372433 |
| NELFE | 7936 | down | -1.584306 | 0.000888 | 0.087917 |
| NEUROG3 | 50674 | down | -1.992002 | 0.034817 | 0.29373 |
| NFATC1 | 4772 | down | -1.63001 | 0.018917 | 0.243912 |
| NFKB2 | 4791 | down | -1.753627 | 0.026548 | 0.29373 |
| NGFR | 4804 | down | -1.907655 | 0.018546 | 0.243912 |
| NIN | 51199 | down | -1.737908 | 0.038737 | 0.329895 |
| NOS1 | 4842 | down | -2.31255 | 0.003629 | 0.145085 |
| NPAS1 | 4861 | down | -2.281163 | 0.006518 | 0.173336 |
| NPAS2 | 4862 | down | -2.263292 | 0.001559 | 0.097436 |
| NPC1L1 | 29881 | down | -1.709462 | 0.012165 | 0.243912 |
| NPEPPS | 9520 | down | -1.553062 | 0.016578 | 0.243912 |
| NPIPA1 | 9284 | down | -2.958304 | 0.000046 | 0 |
| NPR1 | 4881 | down | -1.50569 | 0.035141 | 0.29373 |
| NPR2 | 4882 | down | -1.732407 | 0.015683 | 0.243912 |
| NR4A1 | 3164 | down | -1.987391 | 0.004448 | 0.173336 |
| NRF1 | 4899 | down | -1.663312 | 0.005932 | 0.173336 |
| NRIP2 | 83714 | down | -1.898929 | 0.02151 | 0.29373 |
| NRL | 4901 | down | -1.939583 | 0.014501 | 0.243912 |
| NRXN2 | 9379 | down | -1.83418 | 0.028337 | 0.29373 |
| NUDC | 10726 | down | -1.94063 | 0.036315 | 0.329895 |
| NUP93 | 9688 | down | -1.789872 | 0.022129 | 0.29373 |
| OGFR | 11054 | down | -2.042252 | 0.007621 | 0.211453 |
| OLFM1 | 10439 | down | -1.883744 | 0.029654 | 0.29373 |
| OPCML | 4978 | down | -1.884118 | 0.033989 | 0.29373 |
| OTOF | 9381 | down | -1.562956 | 0.045891 | 0.329895 |
| OTUB1 | 55611 | down | -1.986829 | 0.010092 | 0.211453 |
| OXLD1 | 339229 | down | -2.161986 | 0.000877 | 0.087917 |
| PACSIN3 | 29763 | down | -2.669468 | 0.000875 | 0.087917 |
| PAGR1 | 79447 | down | -1.682908 | 0.035999 | 0.329895 |
| PAH | 5053 | down | -1.875237 | 0.03304 | 0.29373 |
| PAK2 | 5062 | down | -1.62585 | 0.047369 | 0.329895 |
| PAX7 | 5081 | down | -1.959856 | 0.007008 | 0.173336 |
| PAX8 | 7849 | down | -2.162511 | 0.012099 | 0.243912 |
| PBX1 | 5087 | down | -2.159813 | 0.011312 | 0.243912 |
| PCDHB3 | 56132 | down | -1.556764 | 0.039912 | 0.329895 |
| PCSK7 | 9159 | down | -1.991353 | 0.000603 | 0.072128 |
| PDE9A | 5152 | down | -1.683995 | 0.025986 | 0.29373 |
| PDIA2 | 64714 | down | -1.547806 | 0.037557 | 0.329895 |
| PDLIM3 | 27295 | down | -1.613927 | 0.021402 | 0.29373 |
| PDZK1IP1 | 10158 | down | -1.789727 | 0.049221 | 0.372433 |
| PELP1 | 27043 | down | -1.866868 | 0.048322 | 0.329895 |
| PEX10 | 5192 | down | -2.088149 | 0.005038 | 0.173336 |
| PFKFB2 | 5208 | down | -1.924622 | 0.032994 | 0.29373 |
| PGAM2 | 5224 | down | -1.975435 | 0.010099 | 0.211453 |
| PHLPP1 | 23239 | down | -2.014982 | 0.017942 | 0.243912 |
| PITPNM3 | 83394 | down | -1.563825 | 0.023816 | 0.29373 |
| PITX2 | 5308 | down | -1.973407 | 0.009737 | 0.211453 |
| PKN1 | 5585 | down | -1.613972 | 0.014723 | 0.243912 |
| PKNOX1 | 5316 | down | -1.759233 | 0.010984 | 0.243912 |
| PLCH2 | 9651 | down | -1.73213 | 0.042743 | 0.329895 |
| PLEC | 5339 | down | -1.585399 | 0.038267 | 0.329895 |
| PLEKHA4 | 57664 | down | -1.577545 | 0.014004 | 0.243912 |
| PLEKHM2 | 23207 | down | -1.741552 | 0.01234 | 0.243912 |
| PLPPR2 | 64748 | down | -1.58494 | 0.032063 | 0.29373 |
| PLSCR3 | 57048 | down | -1.722143 | 0.005924 | 0.173336 |
| PLXNA1 | 5361 | down | -1.849133 | 0.032634 | 0.29373 |
| PLXNB1 | 5364 | down | -1.797537 | 0.019542 | 0.243912 |
| PLXNB3 | 5365 | down | -1.857218 | 0.011943 | 0.243912 |
| PLXND1 | 23129 | down | -1.50886 | 0.010193 | 0.211453 |
| PML | 5371 | down | -2.425836 | 0.008732 | 0.211453 |
| PNPLA2 | 57104 | down | -2.268289 | 0.000544 | 0.072128 |
| POLD4 | 57804 | down | -1.567604 | 0.004103 | 0.173336 |
| POLG | 5428 | down | -1.840008 | 0.004076 | 0.173336 |
| POLM | 27434 | down | -2.288725 | 0.001904 | 0.121232 |
| PPIL2 | 23759 | down | -1.9776605 | 0.017384 | 0.233533 |
| PPME1 | 51400 | down | -1.540062 | 0.003666 | 0.145085 |
| PPP1R12B | 4660 | down | -1.515513 | 0.029969 | 0.29373 |
| PPP1R37 | 284352 | down | -1.782937 | 0.023275 | 0.29373 |
| PPP2R5A | 5525 | down | -1.998984 | 0.015034 | 0.243912 |
| PPP5C | 5536 | down | -1.657356 | 0.03502 | 0.29373 |
| PRAF2 | 11230 | down | -1.726667 | 0.020572 | 0.29373 |
| PRKCG | 5582 | down | -1.622251 | 0.039955 | 0.329895 |
| PRKCSH | 5589 | down | -1.655213 | 0.000678 | 0.087917 |
| PRKG1 | 5592 | down | -1.860552 | 0.009986 | 0.211453 |
| PROM1 | 8842 | down | -1.607914 | 0.006016 | 0.173336 |
| PRPF31 | 26121 | down | -1.957122 | 0.007665 | 0.211453 |
| PRR14 | 78994 | down | -1.754731 | 0.002258 | 0.121232 |
| PRRC2B | 84726 | down | -1.641295 | 0.000778 | 0.087917 |
| PRRX2 | 51450 | down | -1.554036 | 0.039213 | 0.329895 |
| PRTN3 | 5657 | down | -1.920987 | 0.037951 | 0.329895 |
| PRX | 57716 | down | -2.596739 | 0.001714 | 0.097436 |
| PSG9 | 5678 | down | -1.627566 | 0.030024 | 0.29373 |
| PTOV1 | 53635 | down | -1.638488 | 0.002551 | 0.145085 |
| PTPN21 | 11099 | down | -1.766958 | 0.034782 | 0.29373 |
| PTPRN2 | 5799 | down | -2.252388 | 0.006722 | 0.173336 |
| PYGO1 | 26108 | down | -1.778181 | 0.040257 | 0.329895 |
| RAB33A | 9363 | down | -1.576336 | 0.016347 | 0.243912 |
| RAC2 | 5880 | down | -1.702177 | 0.045797 | 0.329895 |
| RAD51 | 5888 | down | -2.142395 | 0.007018 | 0.173336 |
| RAD54L2 | 23132 | down | -1.909149 | 0.009747 | 0.211453 |
| RAG2 | 5897 | down | -3.298022 | 0.001262 | 0.097436 |
| RAI1 | 10743 | down | -1.818809 | 0.043269 | 0.329895 |
| RANBP1 | 5902 | down | -1.850685 | 0.011827 | 0.243912 |
| RANBP3 | 8498 | down | -1.902616 | 0.025284 | 0.2255635 |
| RARG | 5916 | down | -1.528493 | 0.023782 | 0.29373 |
| RASSF1 | 11186 | down | -2.090019 | 0.012238 | 0.243912 |
| RBM10 | 8241 | down | -2.38366067 | 0.00052 | 0.0586113 |
| RBM19 | 9904 | down | -1.917491 | 0.013893 | 0.243912 |
| RBP3 | 5949 | down | -1.825615 | 0.028021 | 0.29373 |
| RBPMS | 11030 | down | -1.572462 | 0.000191 | 0.0495 |
| RERE | 473 | down | -1.530502 | 0.000766 | 0.087917 |
| RERGL | 79785 | down | -2.072764 | 0.017723 | 0.243912 |
| RFX4 | 5992 | down | -2.385022 | 0.004063 | 0.173336 |
| RGS4 | 5999 | down | -1.582974 | 0.039524 | 0.329895 |
| RGS6 | 9628 | down | -1.542781 | 0.037327 | 0.329895 |
| RIF1 | 55183 | down | -1.950552 | 0.035454 | 0.29373 |
| RNASE1 | 6035 | down | -1.591851 | 0.004597 | 0.173336 |
| RNF220 | 55182 | down | -1.69075 | 0.001191 | 0.087917 |
| ROR2 | 4920 | down | -2.386365 | 0.002708 | 0.145085 |
| RPL36 | 25873 | down | -1.553825 | 0.000721 | 0.087917 |
| RPS19 | 6223 | down | -1.810161 | 0.006587 | 0.173336 |
| RPS21 | 6227 | down | -1.653734 | 0.001461 | 0.097436 |
| RRP7A | 27341 | down | -1.565599 | 0.008233 | 0.211453 |
| RS1 | 6247 | down | -1.854489 | 0.026645 | 0.29373 |
| RSPH14 | 27156 | down | -1.899682 | 0.017171 | 0.243912 |
| RUSC2 | 9853 | down | -1.752816 | 0.033752 | 0.29373 |
| RYR1 | 6261 | down | -1.785824 | 0.045745 | 0.329895 |
| S100A13 | 6284 | down | -1.569445 | 0.000526 | 0.072128 |
| S100A6 | 6277 | down | -1.632336 | 0.007272 | 0.211453 |
| S100A8 | 6279 | down | -2.2769935 | 0.00653 | 0.1923945 |
| S100A9 | 6280 | down | -2.048178 | 0.004263 | 0.173336 |
| S100P | 6286 | down | -2.692725 | 0.015354 | 0.243912 |
| SAC3D1 | 29901 | down | -1.850235 | 0.02153 | 0.29373 |
| SAFB2 | 9667 | down | -2.251147 | 0.00641 | 0.173336 |
| SAGE1 | 55511 | down | -2.062848 | 0.008001 | 0.211453 |
| SBF1 | 6305 | down | -2.397126 | 0.006513 | 0.173336 |
| SBNO2 | 22904 | down | -4.690766 | 0.000053 | 0 |
| SCEL | 8796 | down | -1.876928 | 0.044427 | 0.329895 |
| SCGB1A1 | 7356 | down | -1.704425 | 0.044742 | 0.329895 |
| SCNN1A | 6337 | down | -1.681599 | 0.034492 | 0.29373 |
| SDHB | 6390 | down | -1.909398 | 0.012625 | 0.243912 |
| SEC14L3 | 266629 | down | -1.771161 | 0.014227 | 0.243912 |
| SEMA3B | 7869 | down | -2.051216 | 0.020952 | 0.29373 |
| SENP3 | 26168 | down | -1.875916 | 0.005548 | 0.173336 |
| SEPT8 | 23176 | down | -1.700044 | 0.043269 | 0.329895 |
| SEPT9 | 10801 | down | -1.953534 | 0.045295 | 0.329895 |
| SERPINH1 | 871 | down | -1.716111 | 0.030859 | 0.29373 |
| SF3A2 | 8175 | down | -2.198293 | 0.016226 | 0.2194075 |
| SFRP5 | 6425 | down | -1.631382 | 0.004727 | 0.173336 |
| SFXN3 | 81855 | down | -1.6912665 | 0.003005 | 0.122732 |
| SGCA | 6442 | down | -2.457049 | 0.004354 | 0.173336 |
| SGSM2 | 9905 | down | -1.961739 | 0.016706 | 0.243912 |
| SHANK2 | 22941 | down | -1.745508 | 0.013737 | 0.243912 |
| SIGLEC8 | 27181 | down | -1.826681 | 0.029506 | 0.2869035 |
| SIGLEC9 | 27180 | down | -1.73158 | 0.017793 | 0.243912 |
| SIPA1 | 6494 | down | -1.669889 | 0.026961 | 0.29373 |
| SIX5 | 147912 | down | -2.16611 | 0.00832 | 0.211453 |
| SLC25A21 | 89874 | down | -2.587868 | 0.001938 | 0.121232 |
| SLC26A1 | 10861 | down | -1.674174 | 0.011113 | 0.243912 |
| SLC26A3 | 1811 | down | -1.948258 | 0.029301 | 0.29373 |
| SLC37A1 | 54020 | down | -1.902279 | 0.009932 | 0.211453 |
| SLC48A1 | 55652 | down | -1.64013 | 0.025827 | 0.29373 |
| SLC6A6 | 6533 | down | -1.718682 | 0.007581 | 0.211453 |
| SLC6A8 | 6535 | down | -3.170098 | 0.000287 | 0.0495 |
| SLC7A4 | 6545 | down | -1.735096 | 0.027203 | 0.29373 |
| SLCO1A2 | 6579 | down | -1.609627 | 0.036445 | 0.329895 |
| SMAD6 | 4091 | down | -1.591391 | 0.046823 | 0.329895 |
| SMG5 | 23381 | down | -1.930387 | 0.003712 | 0.145085 |
| SMG6 | 23293 | down | -1.928388 | 0.01677 | 0.243912 |
| SMOX | 54498 | down | -1.9595105 | 0.015767 | 0.195583 |
| SMYD5 | 10322 | down | -2.628069 | 0.000443 | 0.072128 |
| SOCS3 | 9021 | down | -1.991098 | 0.001512 | 0.097436 |
| SOD3 | 6649 | down | -1.619703 | 0.004124 | 0.173336 |
| SOX4 | 6659 | down | -1.77012 | 0.013356 | 0.2276825 |
| SPAG8 | 26206 | down | -2.255072 | 0.006514 | 0.173336 |
| SPC24 | 147841 | down | -1.773673 | 0.007282 | 0.211453 |
| SPDEF | 25803 | down | -1.664003 | 0.046841 | 0.329895 |
| SPIB | 6689 | down | -1.602839 | 0.049926 | 0.372433 |
| SPINK4 | 27290 | down | -2.07002 | 0.008109 | 0.211453 |
| SRC | 6714 | down | -1.901311 | 0.016017 | 0.243912 |
| SRF | 6722 | down | -1.531356 | 0.001452 | 0.097436 |
| SRY | 6736 | down | -1.793979 | 0.038206 | 0.329895 |
| ST18 | 9705 | down | -2.723329 | 0.002349 | 0.121232 |
| ST3GAL2 | 6483 | down | -1.93726 | 0.025558 | 0.29373 |
| STAR | 6770 | down | -2.096399 | 0.003018 | 0.145085 |
| STK10 | 6793 | down | -1.943268 | 0.001729 | 0.097436 |
| STK38 | 11329 | down | -1.617284 | 0.018167 | 0.243912 |
| STXBP6 | 29091 | down | -1.762944 | 0.024055 | 0.29373 |
| SULT1C2 | 6819 | down | -1.596594 | 0.044728 | 0.329895 |
| SUPT6H | 6830 | down | -2.327847 | 0.000482 | 0.072128 |
| SURF2 | 6835 | down | -2.43358 | 0.000791 | 0.087917 |
| SYDE1 | 85360 | down | -1.950071 | 0.012298 | 0.243912 |
| SYK | 6850 | down | -1.508266 | 0.043094 | 0.329895 |
| SYMPK | 8189 | down | -2.988555 | 0.000214 | 0.0495 |
| SYT2 | 127833 | down | -1.572374 | 0.049045 | 0.372433 |
| TAGLN | 6876 | down | -1.553416 | 0.007285 | 0.211453 |
| TAS2R8 | 50836 | down | -1.644476 | 0.034999 | 0.29373 |
| TBCD | 6904 | down | -1.837285 | 0.039917 | 0.329895 |
| TBL3 | 10607 | down | -1.581485 | 0.029495 | 0.29373 |
| TBX2 | 6909 | down | -1.774922 | 0.019356 | 0.2525915 |
| TCF15 | 6939 | down | -1.810673 | 0.015234 | 0.243912 |
| TCHH | 7062 | down | -1.644917 | 0.041142 | 0.329895 |
| TEF | 7008 | down | -2.129571 | 0.017368 | 0.243912 |
| TELO2 | 9894 | down | -1.724763 | 0.012298 | 0.243912 |
| TERT | 7015 | down | -1.533705 | 0.037441 | 0.329895 |
| TFAP2C | 7022 | down | -1.651335 | 0.041572 | 0.329895 |
| TFDP3 | 51270 | down | -1.69355 | 0.023451 | 0.29373 |
| TFEB | 7942 | down | -1.81153 | 0.009386 | 0.211453 |
| TGFB1I1 | 7041 | down | -1.769713 | 0.019274 | 0.243912 |
| TGM1 | 7051 | down | -1.825604 | 0.007858 | 0.211453 |
| TGM3 | 7053 | down | -1.578677 | 0.041893 | 0.329895 |
| TGM4 | 7047 | down | -1.686691 | 0.038414 | 0.329895 |
| TIMP2 | 7077 | down | -2.016183 | 0.003746 | 0.145085 |
| TLE2 | 7089 | down | -1.594998 | 0.01003 | 0.211453 |
| TLN2 | 83660 | down | -1.692768 | 0.03439 | 0.29373 |
| TMEM132A | 54972 | down | -1.721841 | 0.042945 | 0.329895 |
| TMEM176A | 55365 | down | -1.667221 | 0.001009 | 0.087917 |
| TMEM262 | 1E+08 | down | -1.575409 | 0.038618 | 0.329895 |
| TNFAIP2 | 7127 | down | -1.679052 | 0.001362 | 0.097436 |
| TNFSF12 | 8742 | down | -2.484112 | 0.000846 | 0.087917 |
| TNK1 | 8711 | down | -1.927608 | 0.018922 | 0.243912 |
| TNK2 | 10188 | down | -2.0083885 | 0.020103 | 0.268821 |
| TNPO2 | 30000 | down | -1.996824 | 0.022013 | 0.29373 |
| TNS1 | 7145 | down | -1.7748985 | 0.013566 | 0.146865 |
| TNS2 | 23371 | down | -1.569192 | 0.000861 | 0.087917 |
| TNXB | 7148 | down | -1.616827 | 0.001761 | 0.097436 |
| TOM1 | 10043 | down | -2.030187 | 0.000835 | 0.087917 |
| TOR4A | 54863 | down | -1.818789 | 0.000366 | 0.072128 |
| TP53I11 | 9537 | down | -1.991086 | 0.004535 | 0.173336 |
| TPM2 | 7169 | down | -1.678511 | 0.034437 | 0.29373 |
| TPPP3 | 51673 | down | -2.042467 | 0.014268 | 0.243912 |
| TPSAB1 | 7177 | down | -1.8714582 | 0.01822 | 0.2469934 |
| TRAF2 | 7186 | down | -1.664963 | 0.028438 | 0.29373 |
| TRDN | 10345 | down | -2.312003 | 0.015586 | 0.243912 |
| TRIM15 | 89870 | down | -1.6713755 | 0.029091 | 0.2869035 |
| TRIM3 | 10612 | down | -1.848372 | 0.009093 | 0.211453 |
| TRIM46 | 80128 | down | -2.093964 | 0.008913 | 0.211453 |
| TRIO | 7204 | down | -1.82347 | 0.021146 | 0.268821 |
| TRIP6 | 7205 | down | -1.730736 | 0.023543 | 0.29373 |
| TRMT1 | 55621 | down | -1.857744 | 0.013238 | 0.243912 |
| TRNAU1AP | 54952 | down | -1.640236 | 0.021779 | 0.29373 |
| TRPC7 | 57113 | down | -1.572897 | 0.046045 | 0.329895 |
| TRPM4 | 54795 | down | -2.539823 | 0.004011 | 0.173336 |
| TSC2 | 7249 | down | -1.948614 | 0.014504 | 0.243912 |
| TSSC4 | 10078 | down | -1.785506 | 0.02361 | 0.29373 |
| TTC12 | 54970 | down | -1.639636 | 0.029476 | 0.29373 |
| TTC21B | 79809 | down | -2.168637 | 0.01446 | 0.243912 |
| TTC22 | 55001 | down | -1.826407 | 0.02313 | 0.29373 |
| TTC38 | 55020 | down | -1.840343 | 0.005155 | 0.173336 |
| TUBB2A | 7280 | down | -1.519043 | 0.031726 | 0.29373 |
| TUBG2 | 27175 | down | -1.67556 | 0.015901 | 0.243912 |
| UBE2M | 9040 | down | -1.704777 | 0.036886 | 0.329895 |
| UCP2 | 7351 | down | -1.550582 | 0.008451 | 0.211453 |
| ULK1 | 8408 | down | -1.596108 | 0.002954 | 0.145085 |
| UNC119 | 9094 | down | -1.951999 | 0.004825 | 0.173336 |
| UNC93B1 | 81622 | down | -1.907414 | 0.020451 | 0.29373 |
| UNKL | 64718 | down | -1.727799 | 0.033825 | 0.29373 |
| USB1 | 79650 | down | -1.71647 | 0.032019 | 0.29373 |
| USP33 | 23032 | down | -1.79533 | 0.007919 | 0.211453 |
| USP5 | 8078 | down | -1.802893 | 0.010519 | 0.211453 |
| VAMP2 | 6844 | down | -1.607326 | 0.000591 | 0.072128 |
| VCX2 | 51480 | down | -1.817145 | 0.0264 | 0.29373 |
| VGLL1 | 51442 | down | -1.884548 | 0.022349 | 0.29373 |
| VIPR2 | 7434 | down | -1.647677 | 0.0201 | 0.243912 |
| VNN3 | 55350 | down | -1.701734 | 0.018265 | 0.243912 |
| VTCN1 | 79679 | down | -1.624131 | 0.021179 | 0.29373 |
| WDR6 | 11180 | down | -1.657239 | 0.041346 | 0.329895 |
| WISP2 | 8839 | down | -2.176858 | 0.003394 | 0.145085 |
| WNT10B | 7480 | down | -2.435099 | 0.001437 | 0.097436 |
| XYLT2 | 64132 | down | -1.505554 | 0.028522 | 0.29373 |
| YBX3 | 8531 | down | -1.576996 | 0.033612 | 0.29373 |
| ZBP1 | 81030 | down | -1.53573 | 0.030938 | 0.29373 |
| ZBTB48 | 3104 | down | -1.971646 | 0.003623 | 0.145085 |
| ZDHHC18 | 84243 | down | -1.866759 | 0.005596 | 0.173336 |
| ZFHX3 | 463 | down | -1.894962 | 0.006321 | 0.173336 |
| ZFP2 | 80108 | down | -1.827555 | 0.019516 | 0.243912 |
| ZFP36 | 7538 | down | -1.548378 | 0.005816 | 0.173336 |
| ZIC4 | 84107 | down | -2.134433 | 0.028205 | 0.29373 |
| ZMIZ2 | 83637 | down | -2.155644 | 0.005706 | 0.173336 |
| ZNF287 | 57336 | down | -2.274982 | 0.000361 | 0.072128 |
| ZNF358 | 140467 | down | -1.712291 | 0.02409 | 0.29373 |
| ZNF444 | 55311 | down | -2.631532 | 0.000424 | 0.072128 |
| ZNF532 | 55205 | down | -1.804819 | 0.017289 | 0.243912 |
| ZNF574 | 64763 | down | -1.772682 | 0.017641 | 0.243912 |
| ZNF576 | 79177 | down | -1.923109 | 0.014452 | 0.243912 |
| ZNF839 | 55778 | down | -1.744664 | 0.005842 | 0.173336 |
| ZNHIT2 | 741 | down | -1.933323 | 0.016816 | 0.243912 |

**Table S7. HCC-specific differentially expressed intersection miRNAs from GSE21362 and GSE63046**

| **miRNA** | **style** | **GSE21362** | | | **GSE63046** | | |
| --- | --- | --- | --- | --- | --- | --- | --- |
|  |  | **Fold Change** | **p-value** | **FDR** | **Fold Change** | **p-value** | **FDR** |
| hsa-miR-32-5p | up | 1.784915 | 0.000838 | 0.004096 | 1.664472 | 0.035826 | 0.346788 |
| hsa-miR-130b-3p | up | 2.11456 | 0 | 0 | 1.69499 | 0.012248 | 0.264844 |
| hsa-miR-193b-3p | up | 1.509536 | 0.000117 | 0.00075 | 2.119039 | 0.002154 | 0.154573 |
| hsa-miR-1268a | up | 1.638099 | 0.000001 | 0.000013 | 1.651615 | 0.015906 | 0.268171 |
| hsa-miR-1269a | up | 1.535071 | 0.001421 | 0.0063 | 18.77979 | 0.000519 | 0.12227 |
| hsa-miR-30a-3p | down | -1.97207 | 0 | 0.000001 | 0.530832 | 0.013149 | 0.264844 |
| hsa-miR-31-5p | down | -1.89909 | 0.00097 | 0.004485 | 0.427578 | 0.006902 | 0.219466 |
| hsa-miR-199a-5p | down | -4.12166 | 0 | 0 | 0.180306 | 0.001591 | 0.154573 |
| hsa-miR-199a-3p | down | -4.37837 | 0 | 0 | 0.184586 | 0.002703 | 0.154573 |
| hsa-miR-139-5p | down | -2.67128 | 0 | 0 | 0.44523 | 0.001136 | 0.12227 |
| hsa-miR-10a-5p | down | -3.01293 | 0 | 0 | 0.329571 | 0.008093 | 0.231052 |
| hsa-miR-181a-3p | down | -1.82664 | 0.000017 | 0.000152 | 0.593679 | 0.012435 | 0.264844 |
| hsa-miR-214-3p | down | -2.68834 | 0 | 0 | 0.215997 | 0.006545 | 0.212954 |
| hsa-miR-223-3p | down | -2.1032 | 0 | 0 | 0.445486 | 0.001011 | 0.12227 |
| hsa-miR-200b-3p | down | -5.00039 | 0 | 0 | 0.188731 | 0.000442 | 0.12227 |
| hsa-miR-130a-3p | down | -1.95408 | 0 | 0 | 0.502221 | 0.008344 | 0.232575 |
| hsa-miR-142-5p | down | -2.12629 | 0.00015 | 0.000933 | 0.397949 | 0.003208 | 0.15524 |
| hsa-miR-142-3p | down | -2.29999 | 0.000043 | 0.000346 | 0.489619 | 0.009961 | 0.244474 |
| hsa-miR-125a-5p | down | -1.82893 | 0 | 0.000001 | 0.354097 | 0.003152 | 0.15524 |
| hsa-miR-146a-5p | down | -2.05181 | 0.000004 | 0.000041 | 0.487405 | 0.014296 | 0.265825 |
| hsa-miR-150-5p | down | -3.24792 | 0 | 0 | 0.268358 | 0.003698 | 0.166901 |
| hsa-miR-195-5p | down | -1.79857 | 0 | 0.000003 | 0.476316 | 0.002869 | 0.154573 |
| hsa-miR-200a-3p | down | -6.64048 | 0 | 0 | 0.18644 | 0.000505 | 0.12227 |
| hsa-miR-376c-3p | down | -1.87935 | 0.011566 | 0.033834 | 0.566605 | 0.024084 | 0.311972 |
| hsa-miR-375 | down | -5.02511 | 0 | 0 | 0.208306 | 0.002401 | 0.154573 |
| hsa-miR-342-3p | down | -1.61318 | 0 | 0.000003 | 0.580467 | 0.027669 | 0.333694 |
| hsa-miR-338-3p | down | -1.51238 | 0.011093 | 0.032848 | 0.518216 | 0.031915 | 0.340838 |
| hsa-miR-424-5p | down | -2.86114 | 0 | 0 | 0.385334 | 0.000668 | 0.12227 |
| hsa-miR-429 | down | -3.46582 | 0 | 0.000003 | 0.200558 | 0.000656 | 0.12227 |
| hsa-miR-450a-5p | down | -1.57257 | 0.002495 | 0.009763 | 0.498266 | 0.01033 | 0.24494 |
| hsa-miR-497-5p | down | -1.73917 | 0.000015 | 0.000138 | 0.593678 | 0.031248 | 0.340838 |
| hsa-miR-542-3p | down | -1.81191 | 0.00018 | 0.001104 | 0.523542 | 0.019711 | 0.290265 |
| hsa-miR-139-3p | down | -1.57368 | 0.014836 | 0.040436 | 0.497477 | 0.013441 | 0.264844 |
| hsa-miR-214-5p | down | -2.53414 | 0.000001 | 0.000007 | 0.239509 | 0.00281 | 0.154573 |
| hsa-miR-132-5p | down | -1.56743 | 0.006544 | 0.021316 | 0.649451 | 0.030893 | 0.340838 |
| hsa-miR-145-3p | down | -1.74334 | 0.000397 | 0.00216 | 0.408089 | 0.012405 | 0.264844 |

**Table S8. Long-term outcomes for lncRNA EGOT and SERHL (N = 377)**

|  | **EGOT ^†^** | | | **SERHL^‡^** | | |
| --- | --- | --- | --- | --- | --- | --- |
|  | **High expression** | **Low expression** |  | **High expression** | **Low expression** |  |
|  | **(n = 189, 50.1%)** | **(n = 188, 49.9%)** | ***P* value** | **(n = 245, 99.4%)** | **(n = 245, 99.4%)** | ***P* value** |
| Crude mortality | 78 (41.3%) | 54 (28.7%) | 0.011 | 80 (42.3%) | 52 (27.7%) | 0.003 |
| Unadjusted HR (95% CI) | 1.48 (1.05-2.09) | 1 [Reference] | 0.027 | 1.97 (1.39-2.81) | 1 [Reference] | < 0.001 |
| Adjusted HR (95% CI)* | 1.35 (0.95-1.92) | 1 [Reference] | 0.091 | 1.74 (1.22-2.49) | 1 [Reference] | 0.002 |

*Adjusted for selected statistically different variables by multivariate Cox proportional hazards regression analysis (pathologic stage, AFP and albumin) .

**^†^**C statistics = 0.671 (95% CI 0.618-0.724), P < 0.001 (Likelihood ratio test); P < 0.001 (Wald test); P < 0.001 (Score log-rank test).

**^‡^**C statistics = 0.682 (95% CI 0.633-0.731), P < 0.001 (Likelihood ratio test); P < 0.001 (Wald test); P < 0.001 (Score log-rank test).
